# Supplementary material for: Nuclear m6A reader YTHDC1 promotes muscle stem cell activation/proliferation by regulating mRNA splicing and nuclear export
Source: eLife. 2023 Mar 9;12:e82703. doi: 10.7554/eLife.82703 (PMC10089659; doi:10.7554/eLife.82703)
Supplement: Figure 1—source data 2. [file elife-82703-fig1-data2.zip › Figure 1 source data2/Figure 1E-with all relevant bands labelled.docx]

**Figure 1E-YTHDC1**

**
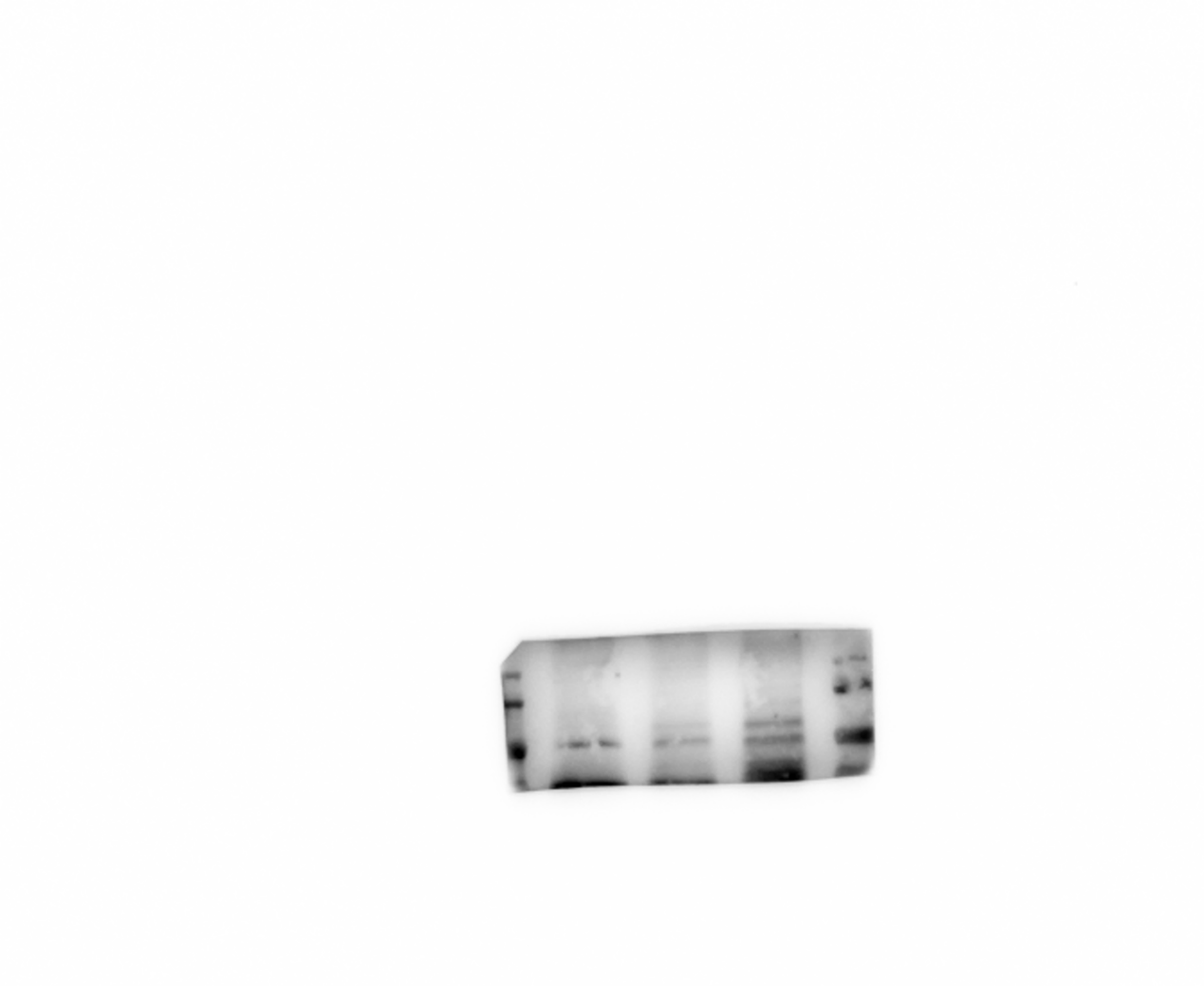
**

**100kDa marker**

**Anti-YTHDC1**

**ASC48**

**ASC24**

**FSIC**

**Figure 1E-Myod**

**
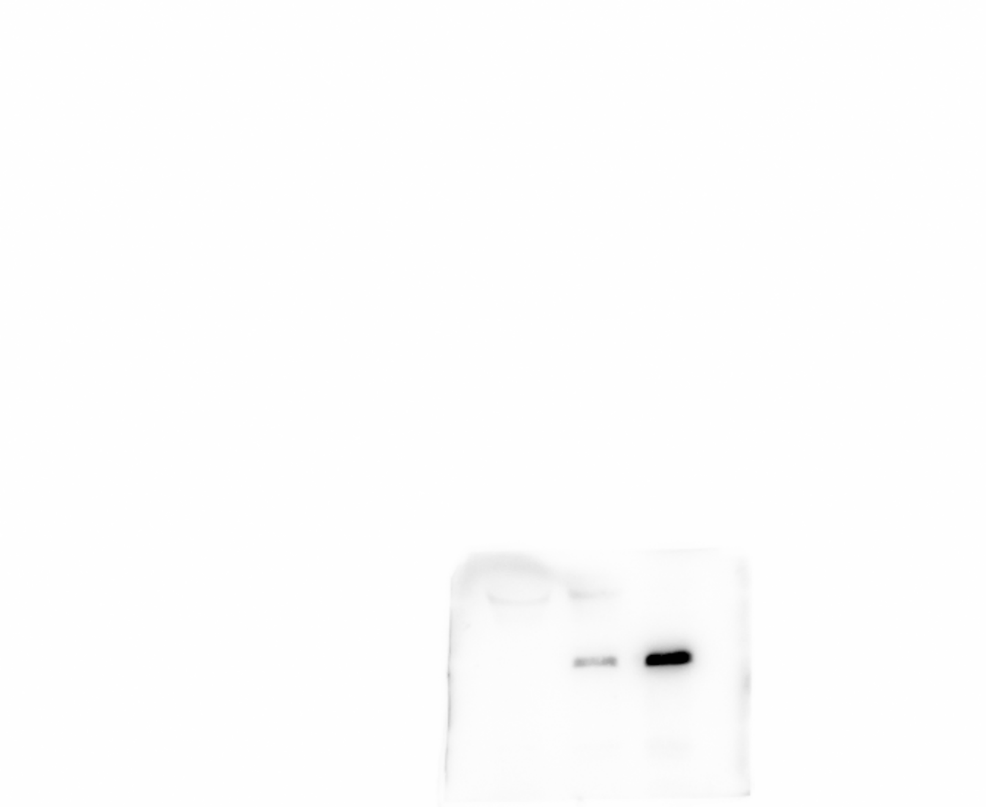
**

**Anti-Myod**

**FSIC**

**ASC24**

**ASC48**

**Figure 1E-Myod merged with marker**

**
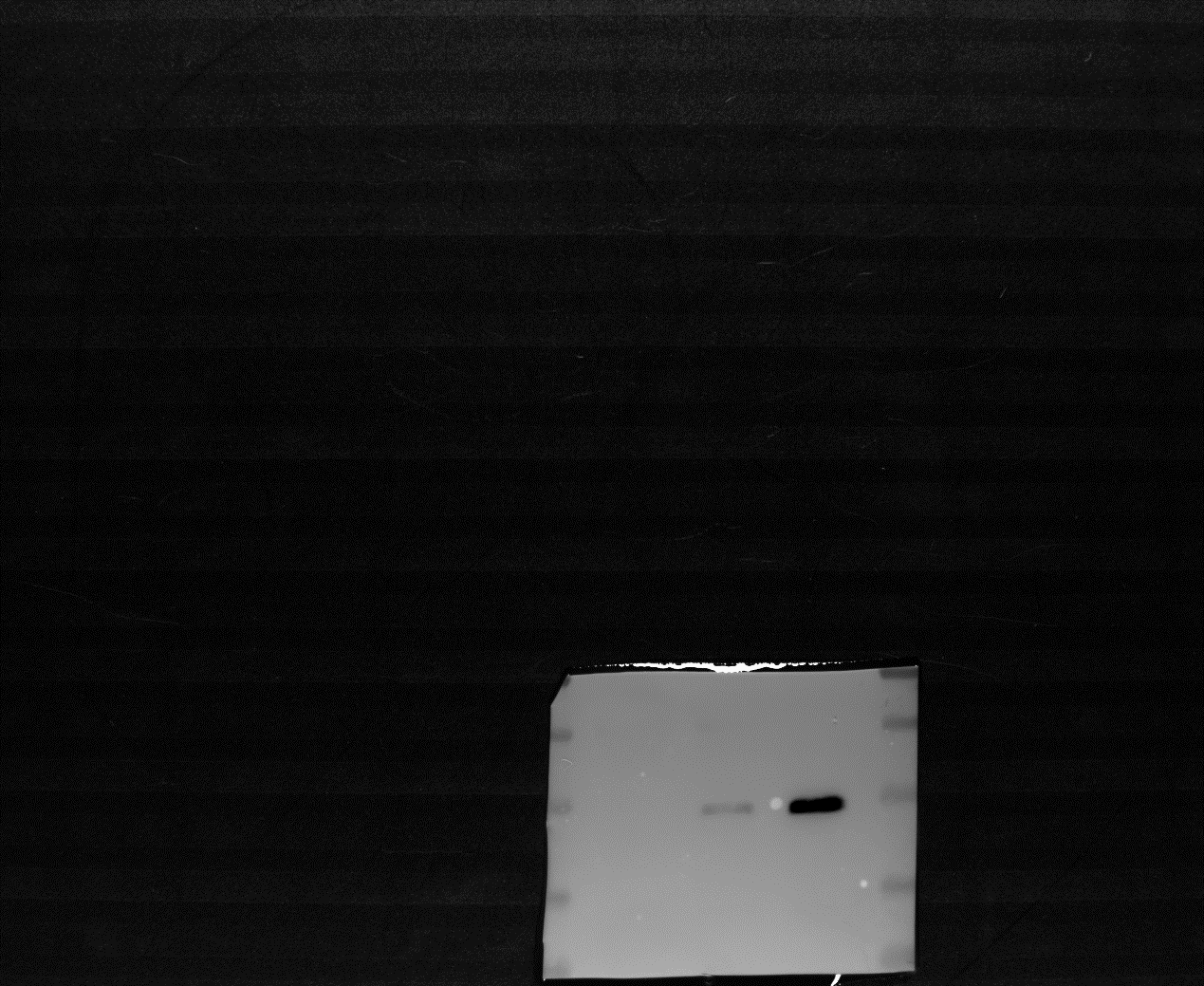
**

**Anti-Myod**

**45kDa marker**

**ASC48**

**ASC24**

**FSIC**

**
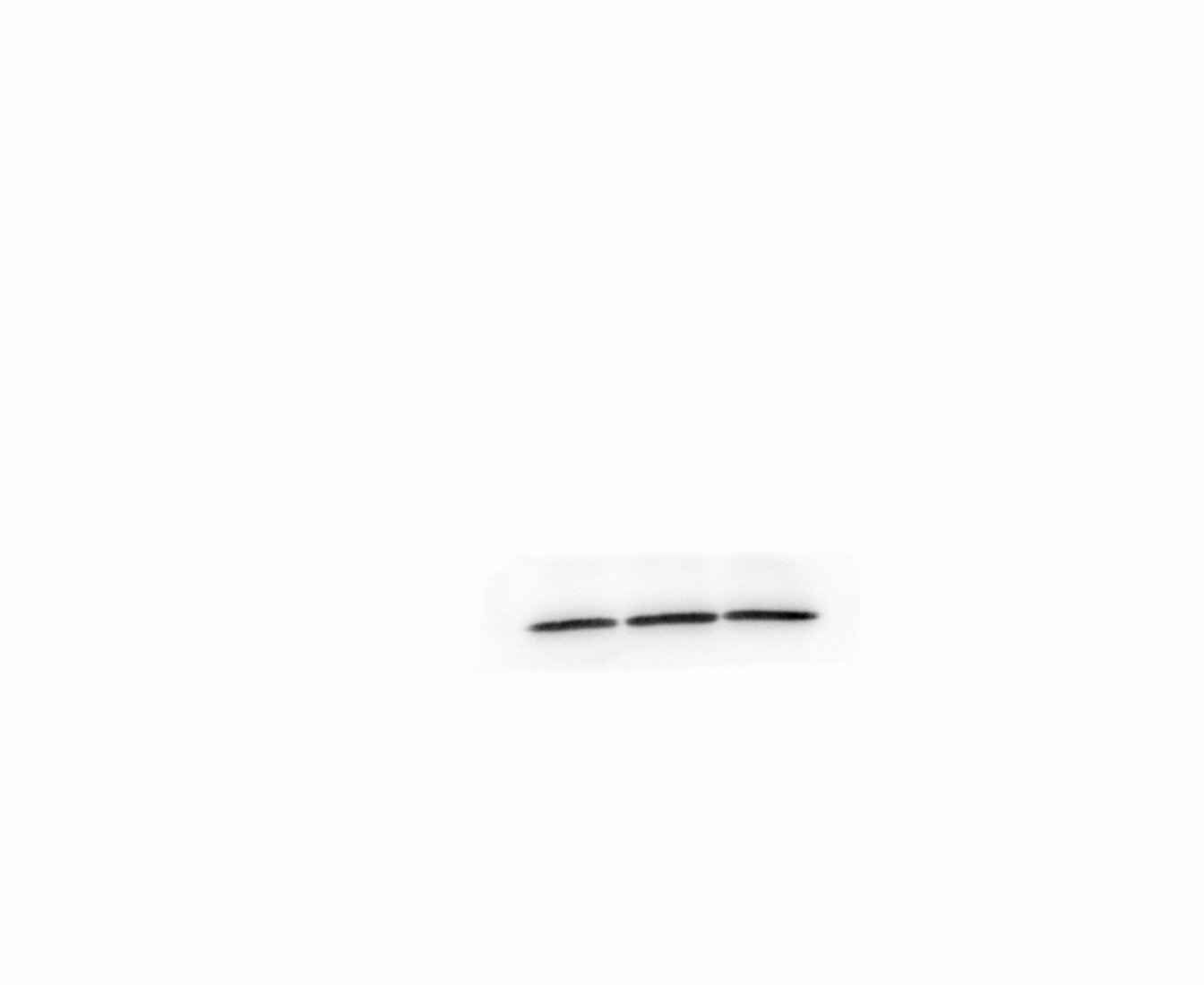
Figure 1E-Histone H3**

**Anti-H3**

**1s exposure**

**ASC48**

**ASC24**

**FSIC**


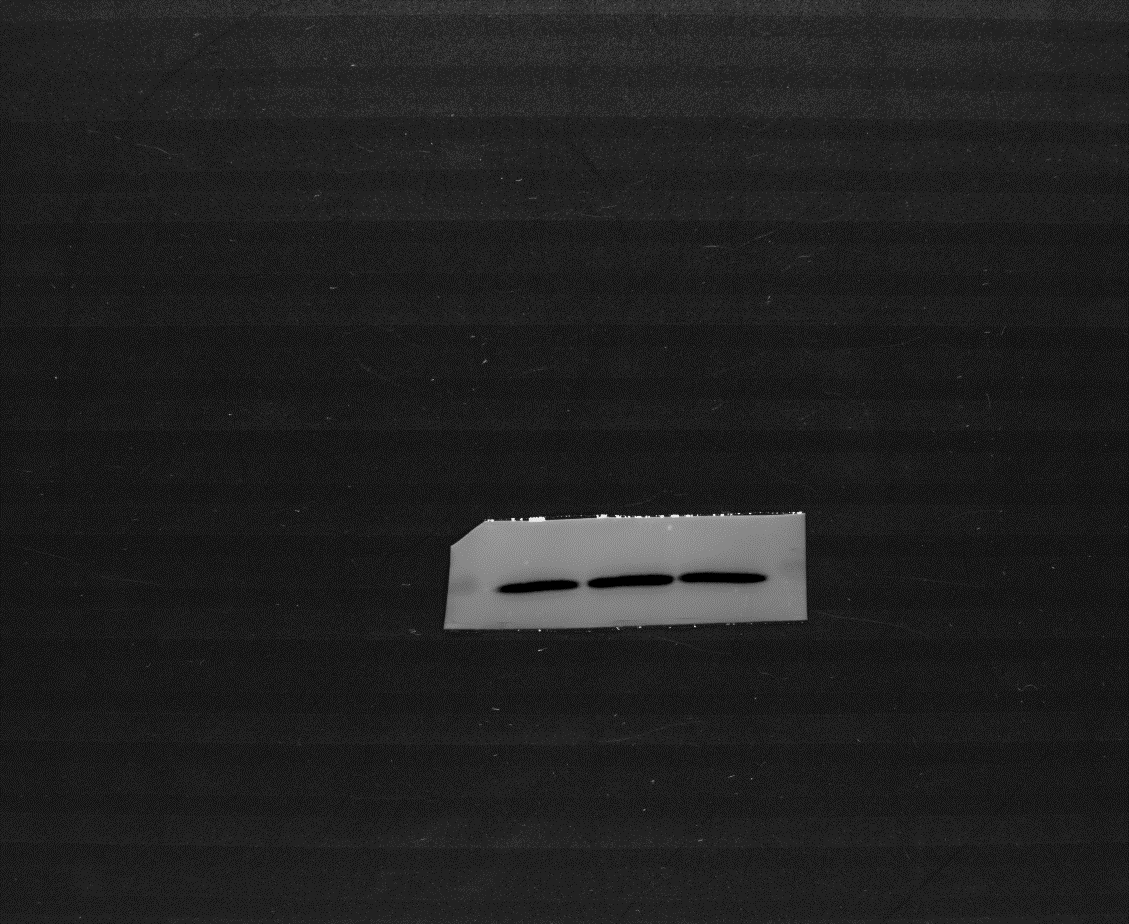


**Anti-H3**

**3s exposure, merged with marker**

**15kDa marker**

**FSIC**

**ASC24**

**ASC48**

**Figure 1E-YTHDF1,2**


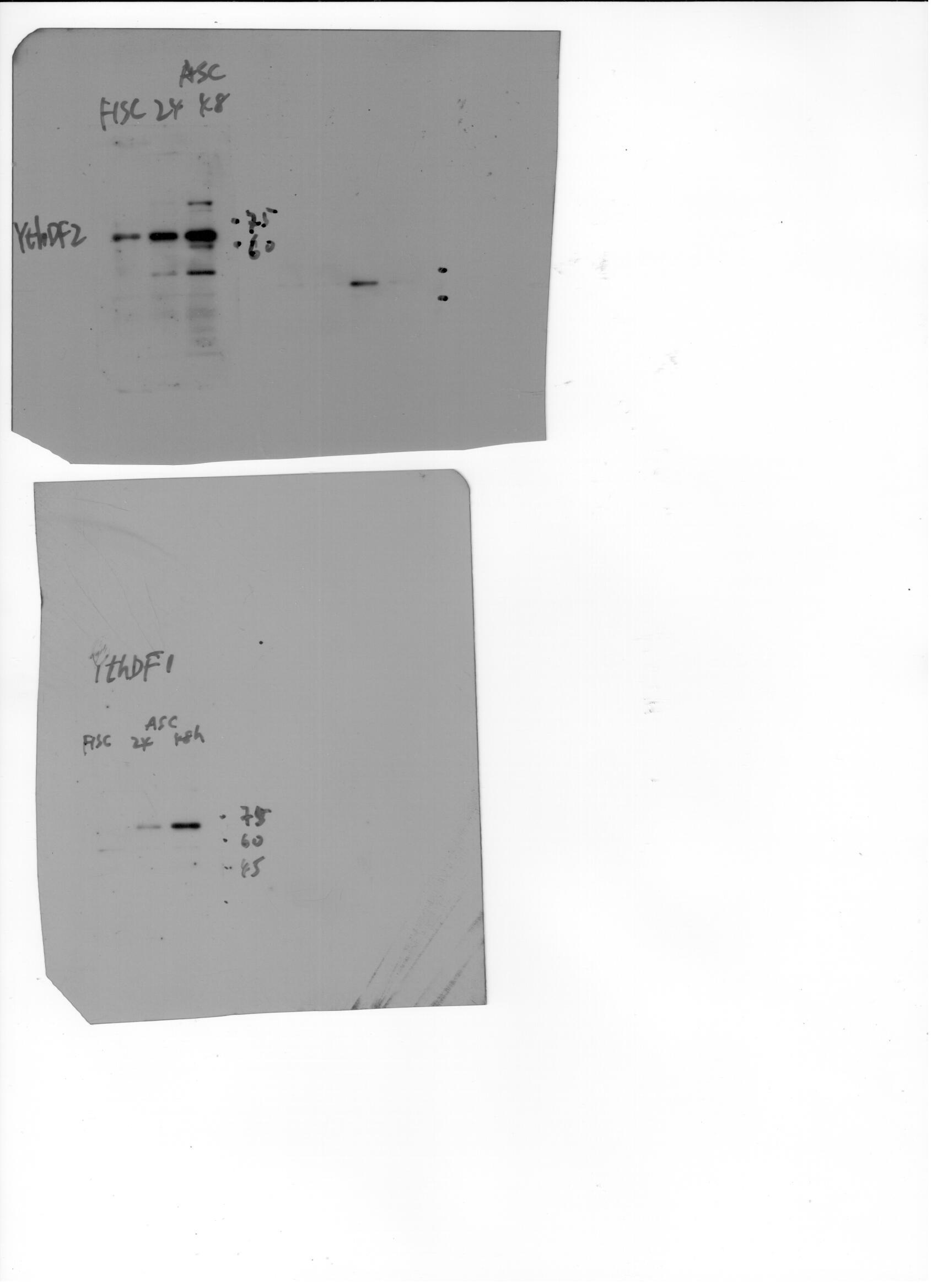


**FSIC**

**ASC24**

**ASC48**

**ASC48**

**FSIC**

**ASC24**

**YTHDF1**

**YTHDF2**


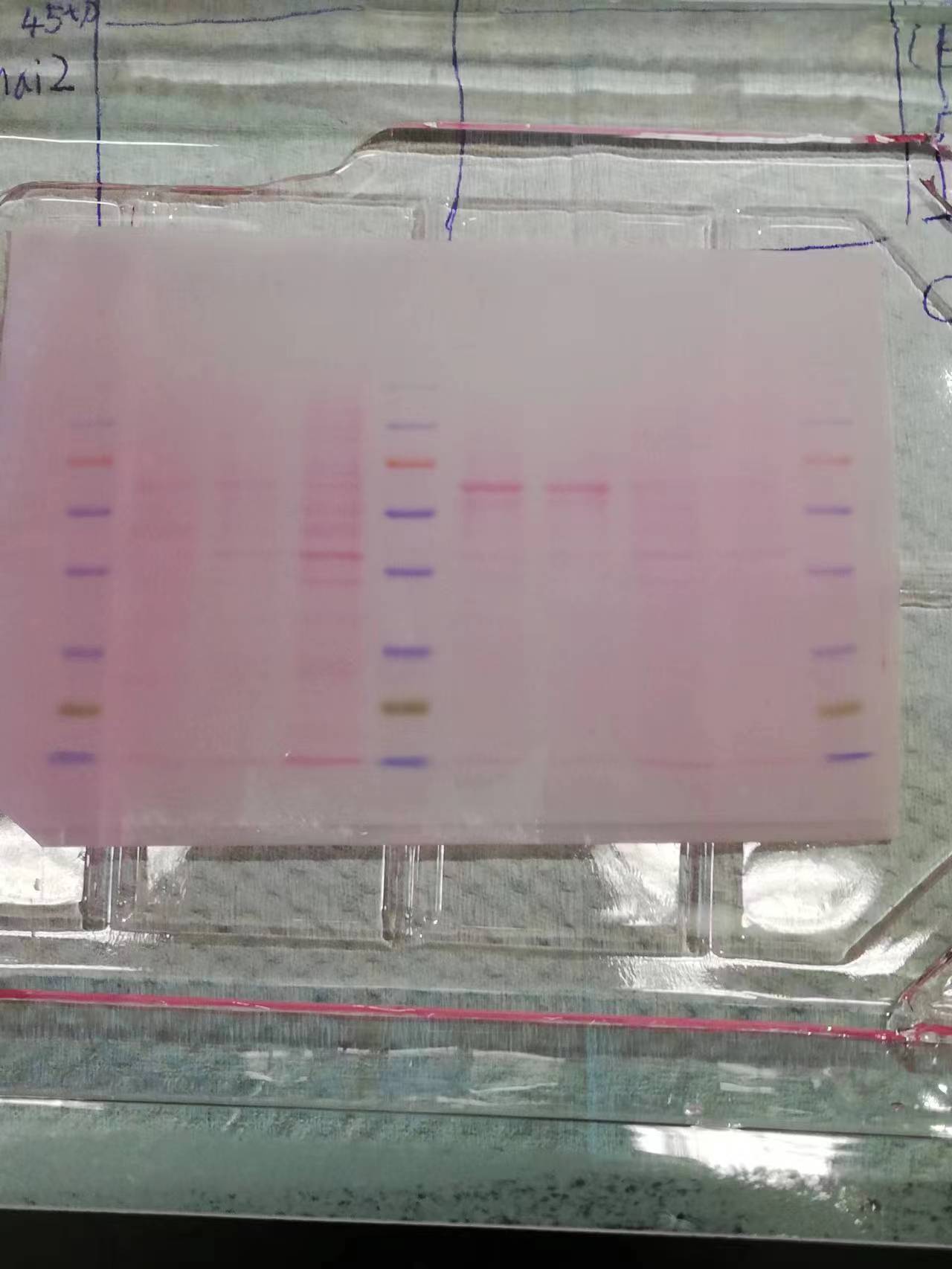


**FSIC**

**ASC24**

**ASC48**

**Loading control for YTHDF1/2, not shown in figure 1E**
