# Supplementary material for: Nuclear m6A reader YTHDC1 promotes muscle stem cell activation/proliferation by regulating mRNA splicing and nuclear export
Source: eLife. 2023 Mar 9;12:e82703. doi: 10.7554/eLife.82703 (PMC10089659; doi:10.7554/eLife.82703)

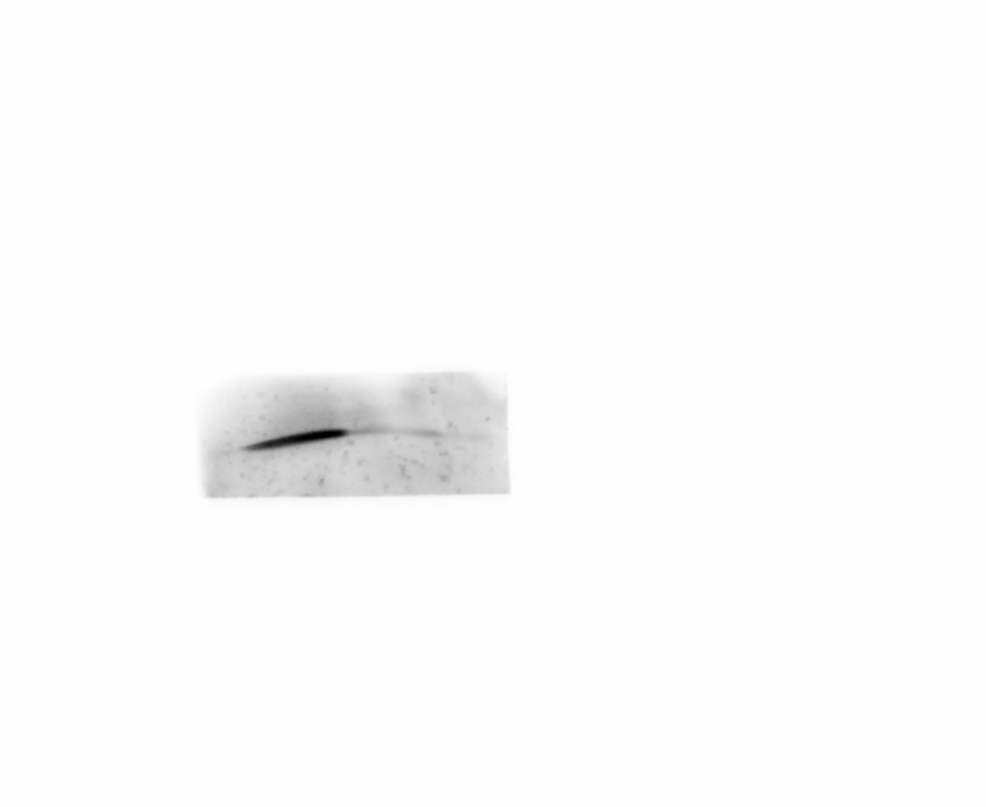
Figure 1G-Histone H3

**Nuc Cyto**


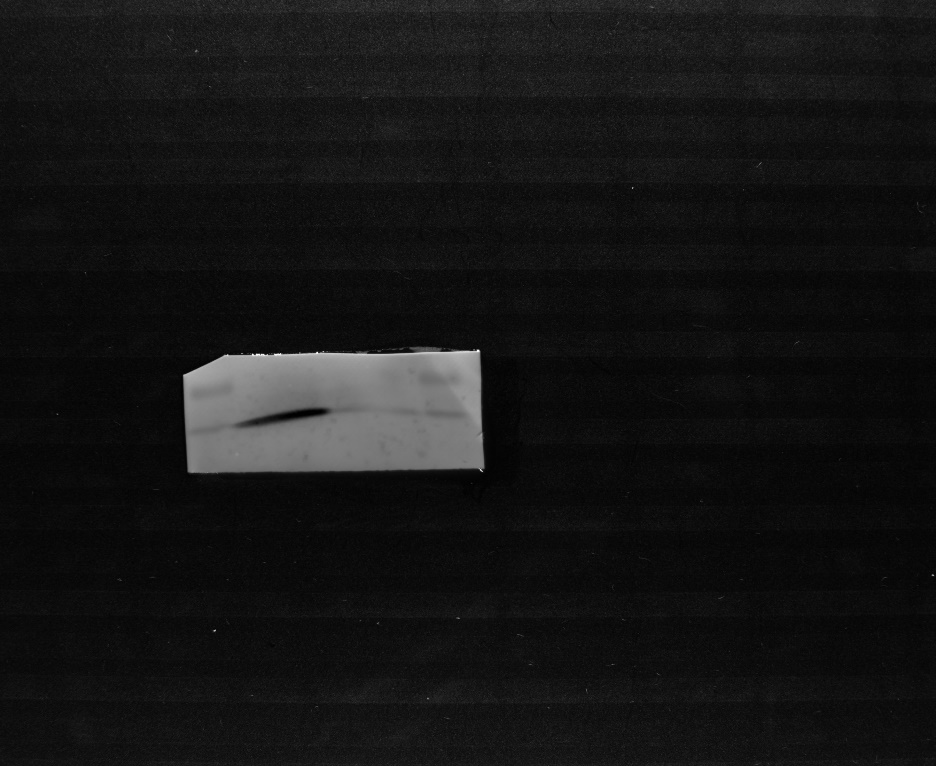


**15kDa marker**

**Histone H3**

**Nuc Cyto**

**Histone H3**


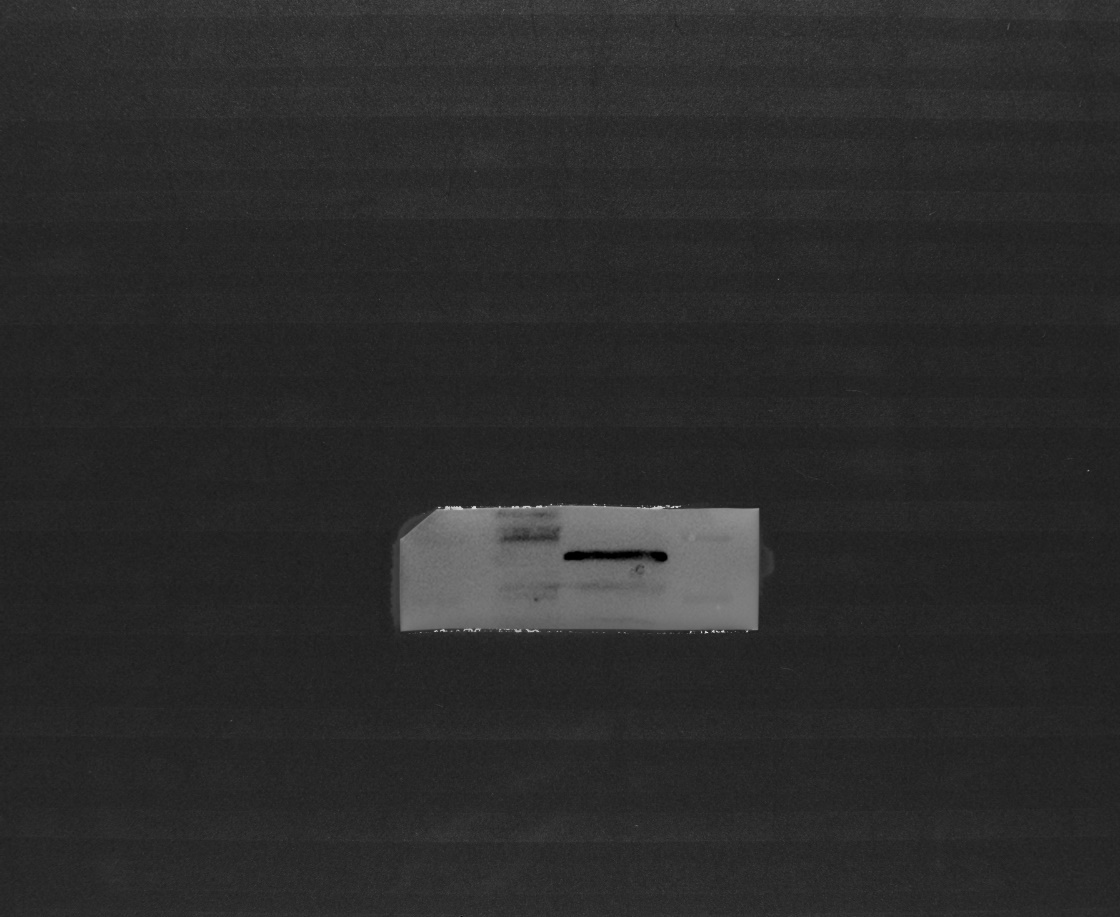
Figure 1G-tublin

**60kDa marker**

**tublin**

**Nuc Cyto**

Figure 1G YTHDC1

**100kDa marker**

**Nuc Cyto**

**YTHDC1**


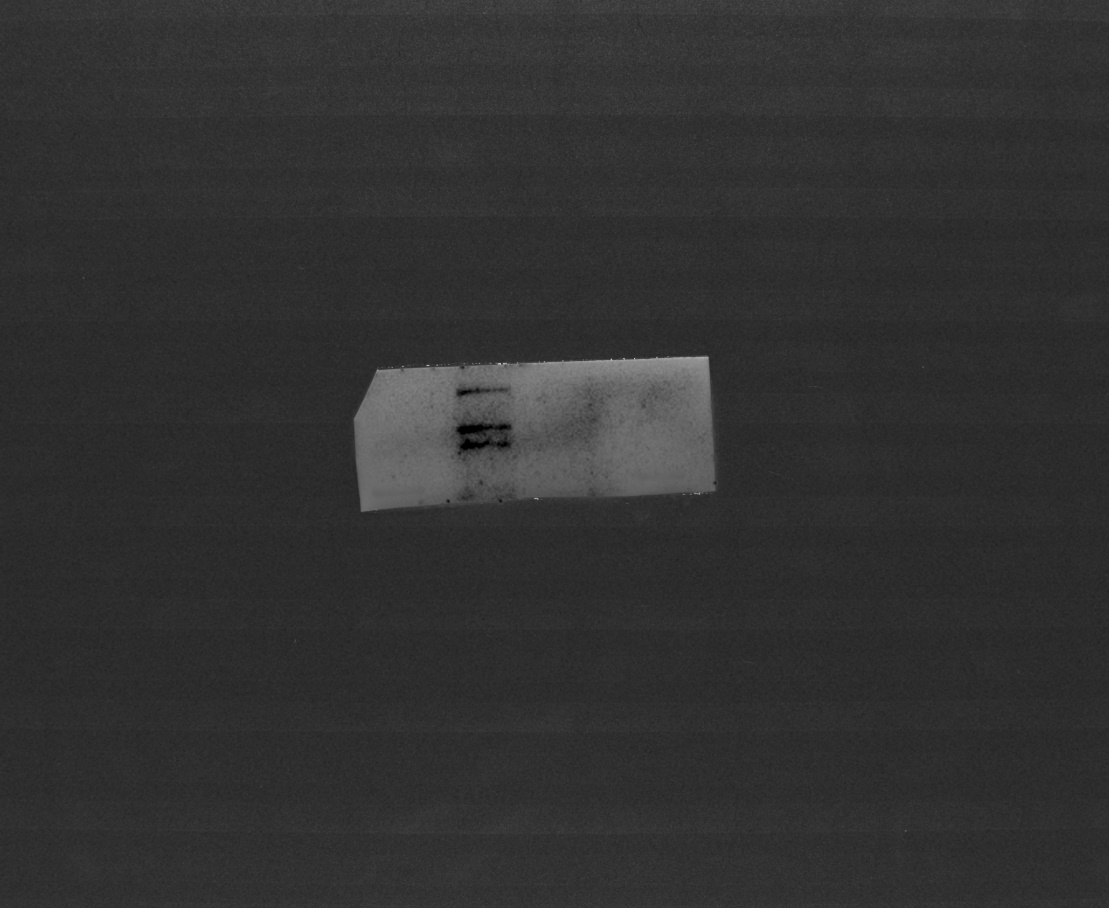

Supplement: Figure 1—source data 2. [file elife-82703-fig1-data2.zip › Figure 1 source data2/Figure 1G-with all relevant bands labelled.docx]
