# Supplementary figures and images for: Nuclear m6A reader YTHDC1 promotes muscle stem cell activation/proliferation by regulating mRNA splicing and nuclear export
Source: eLife. 2023 Mar 9;12:e82703. doi: 10.7554/eLife.82703 (PMC10089659; doi:10.7554/eLife.82703)

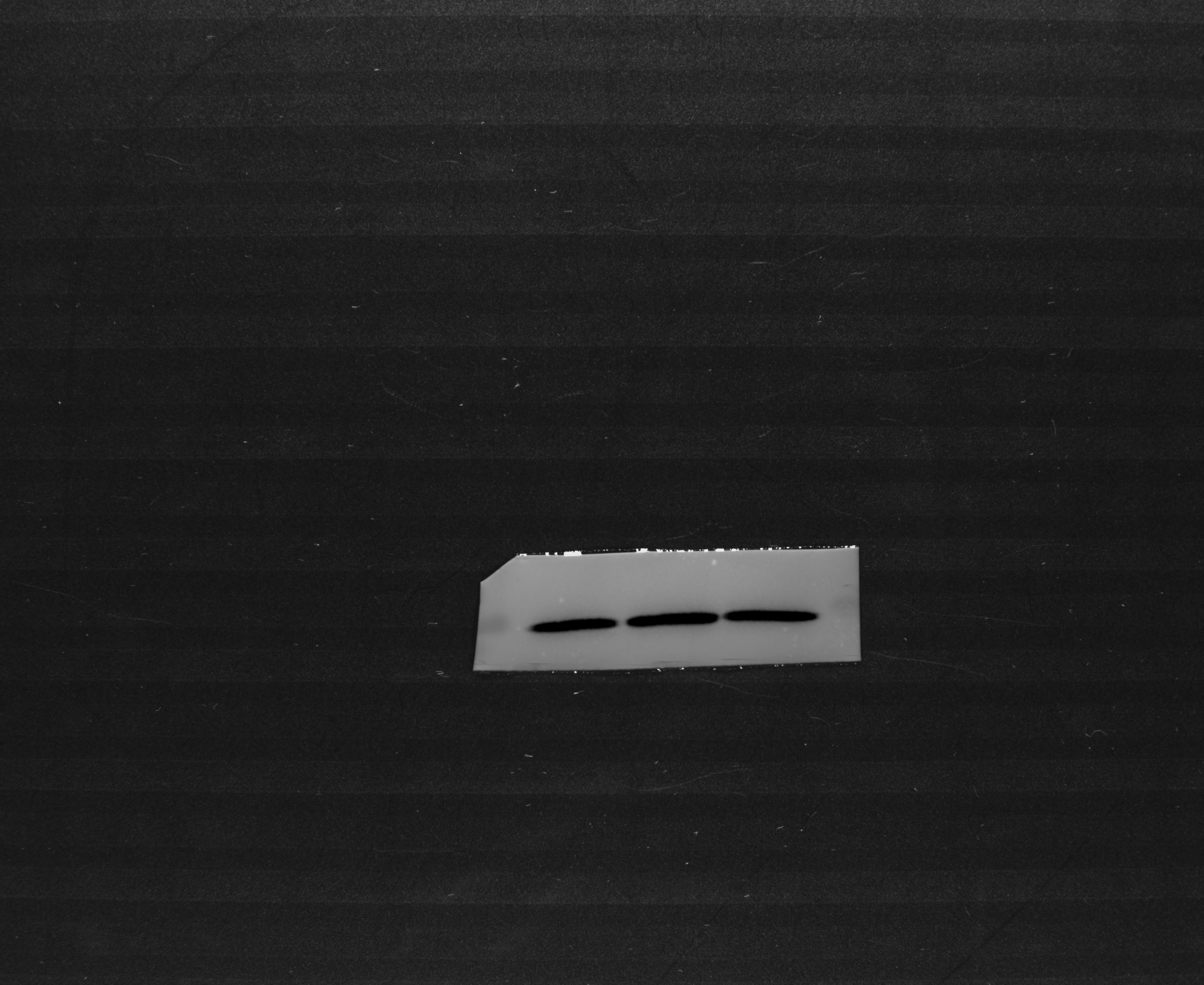

Supplement: Figure 1—source data 2. [file elife-82703-fig1-data2.zip › Figure 1 source data2/Figure 1E-Histone H3-merged with marker.jpg]

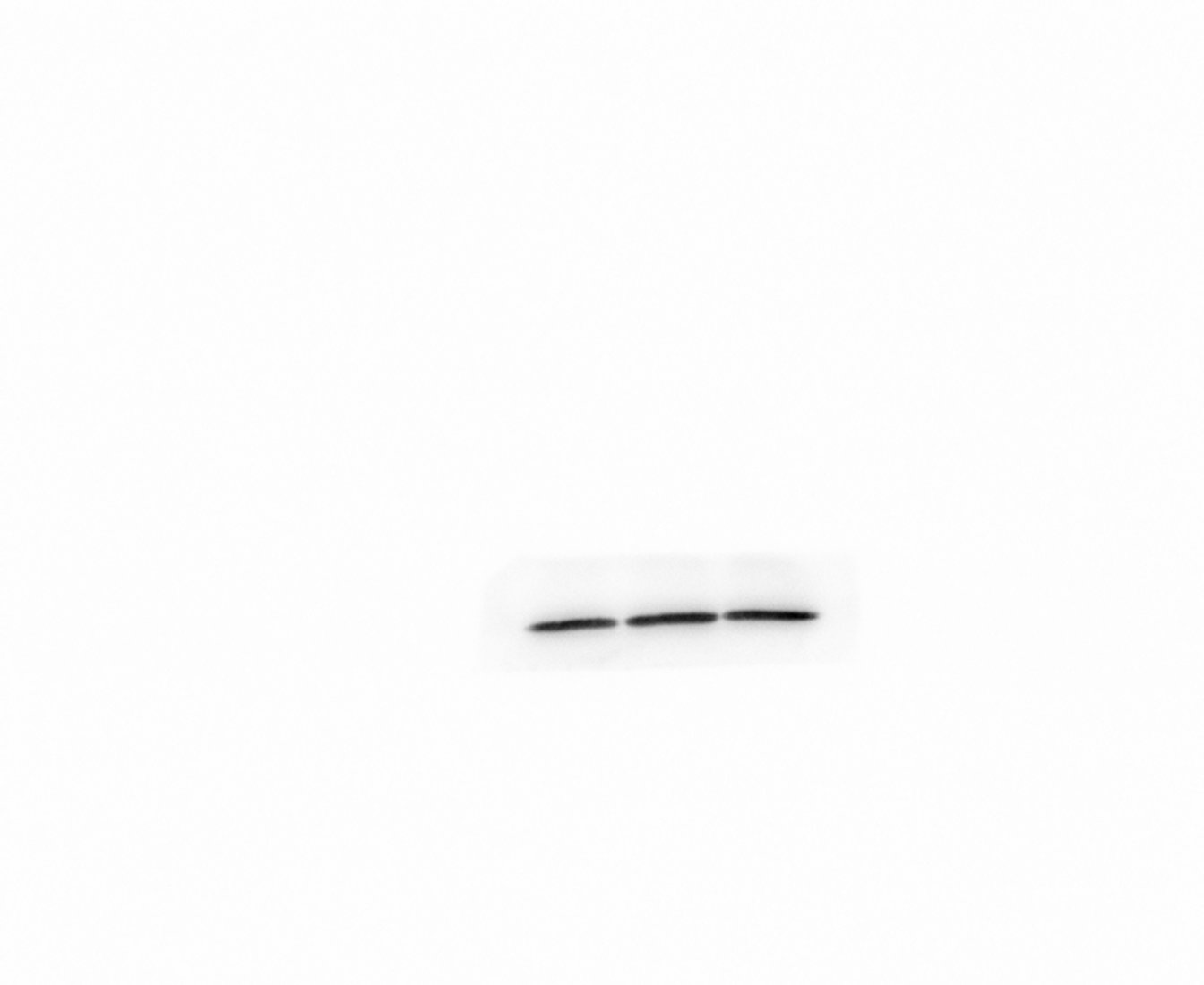

Supplement: Figure 1—source data 2. [file elife-82703-fig1-data2.zip › Figure 1 source data2/Figure 1E-Histone H3-raw-invered.jpg]

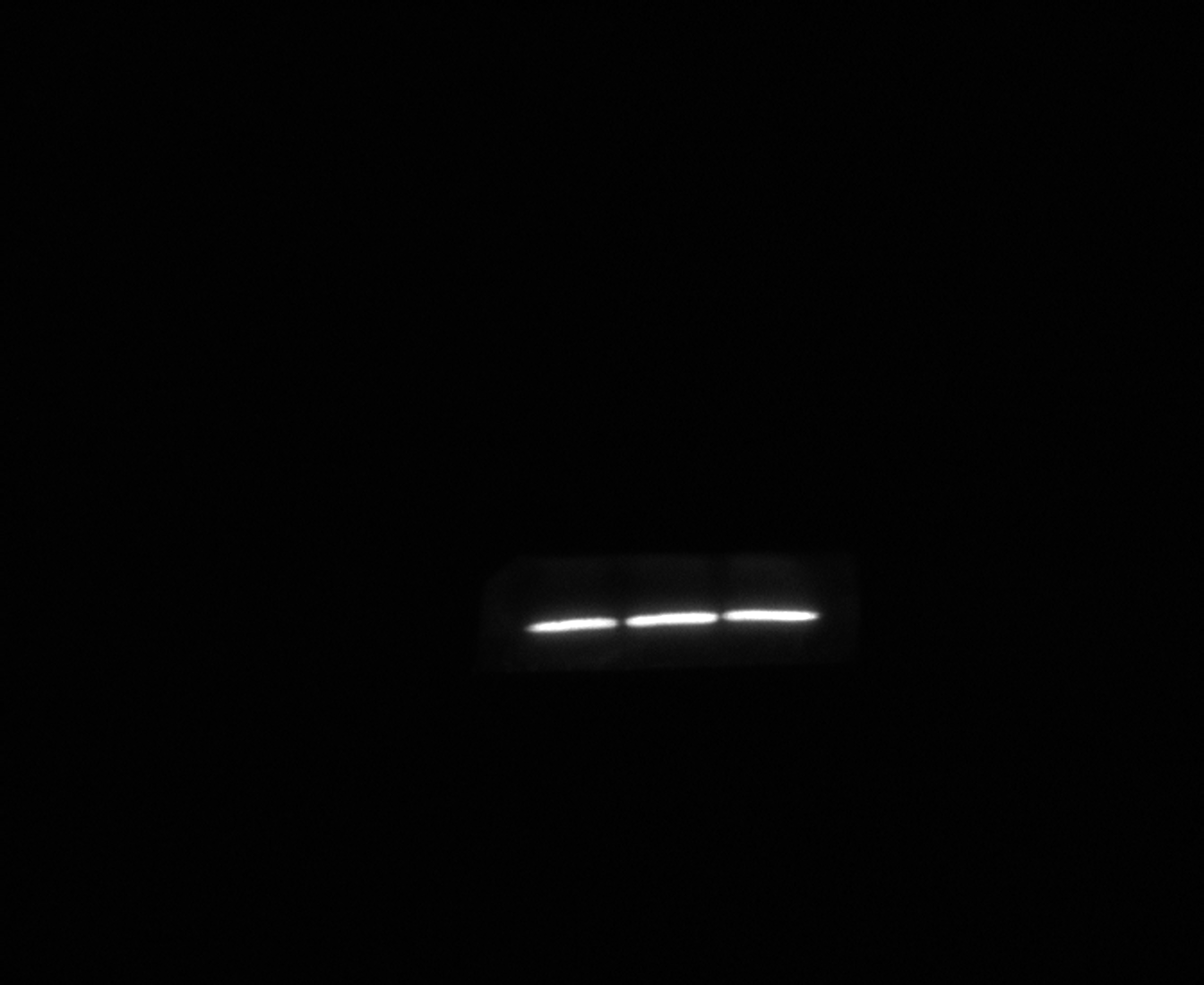

Supplement: Figure 1—source data 2. [file elife-82703-fig1-data2.zip › Figure 1 source data2/Figure 1E-Histone H3-raw.Tif]

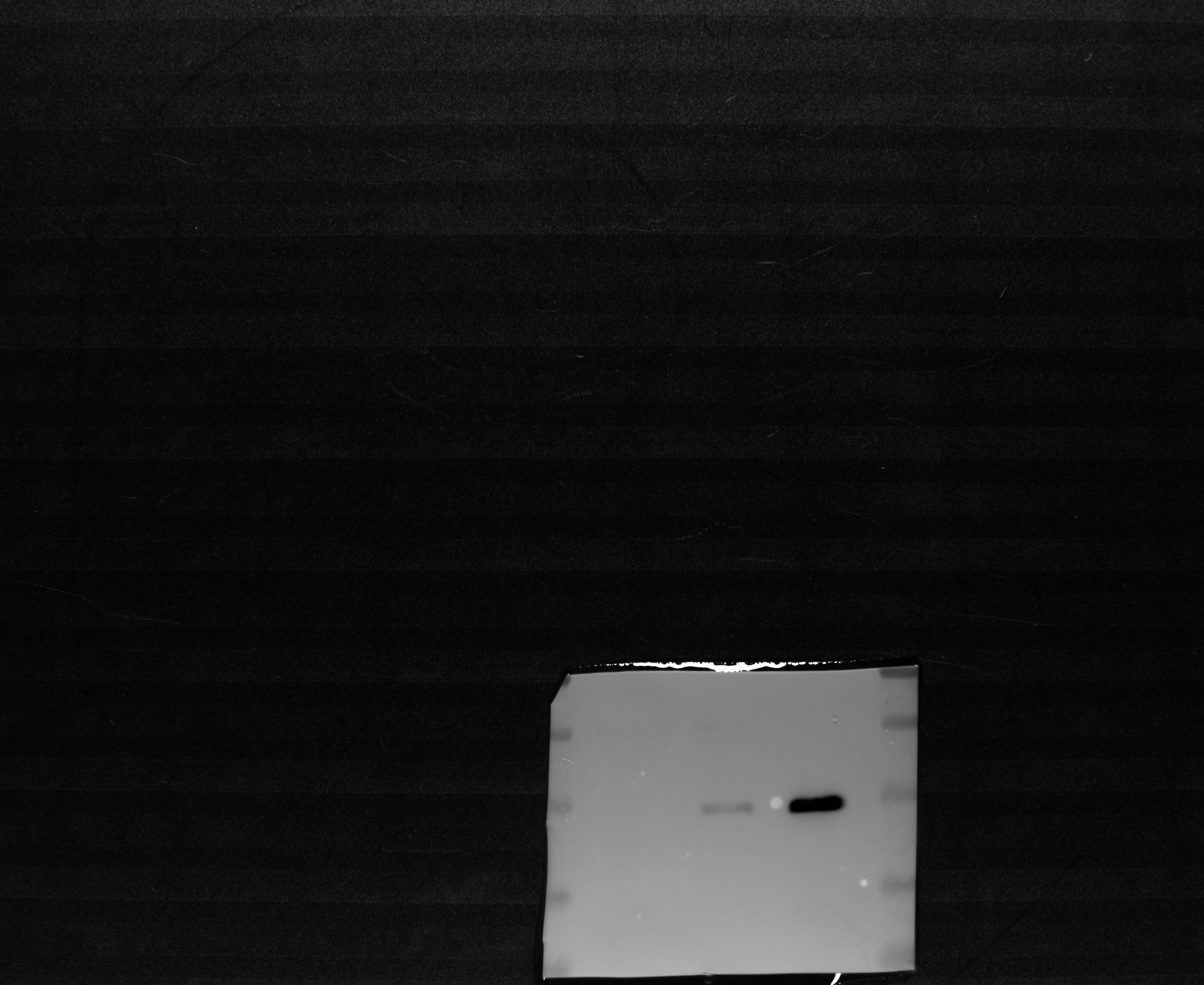

Supplement: Figure 1—source data 2. [file elife-82703-fig1-data2.zip › Figure 1 source data2/Figure 1E-Myod-merged with marker.jpg]

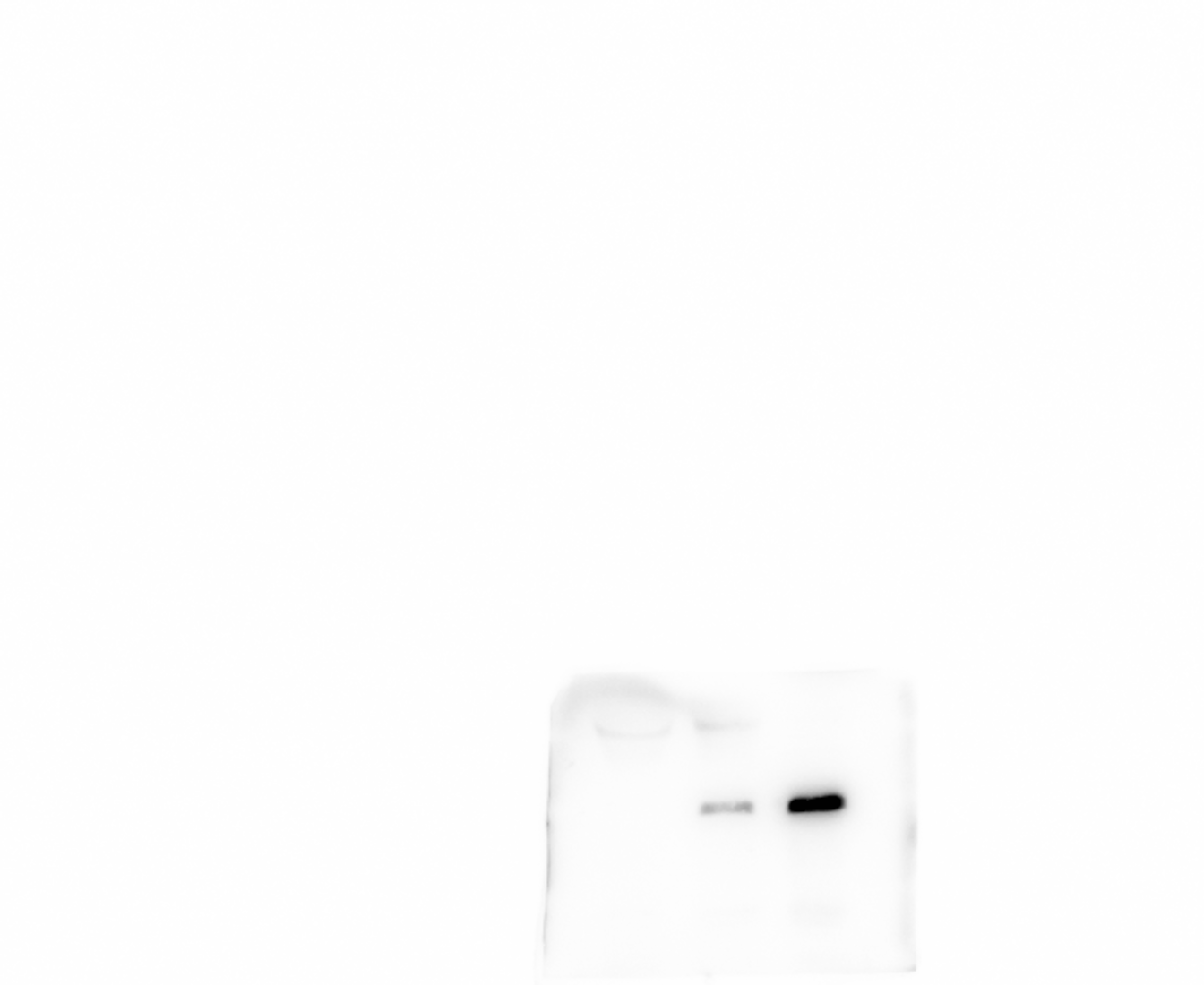

Supplement: Figure 1—source data 2. [file elife-82703-fig1-data2.zip › Figure 1 source data2/Figure 1E-Myod-raw-inverted.Tif]

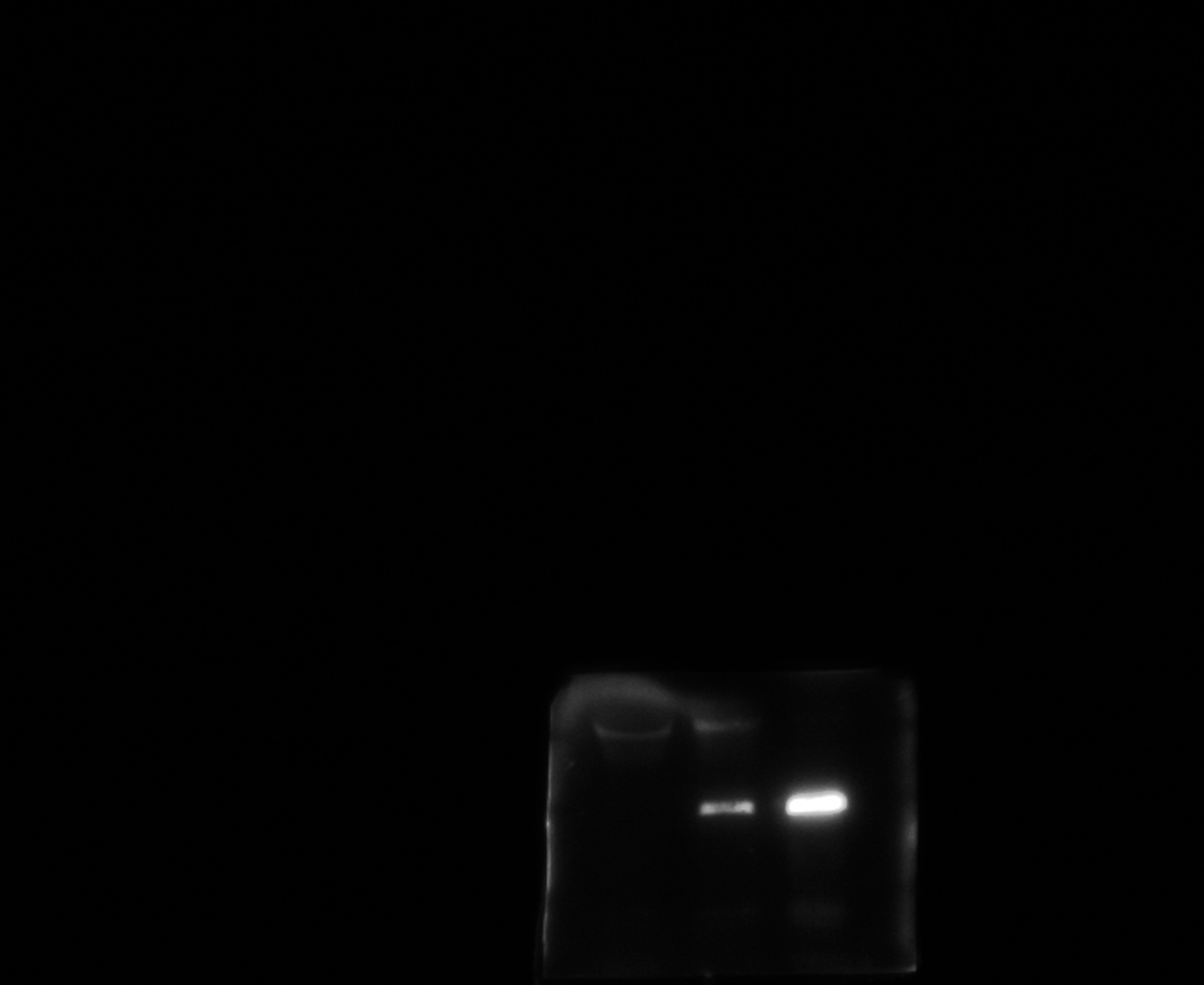

Supplement: Figure 1—source data 2. [file elife-82703-fig1-data2.zip › Figure 1 source data2/Figure 1E-Myod-raw.Tif]

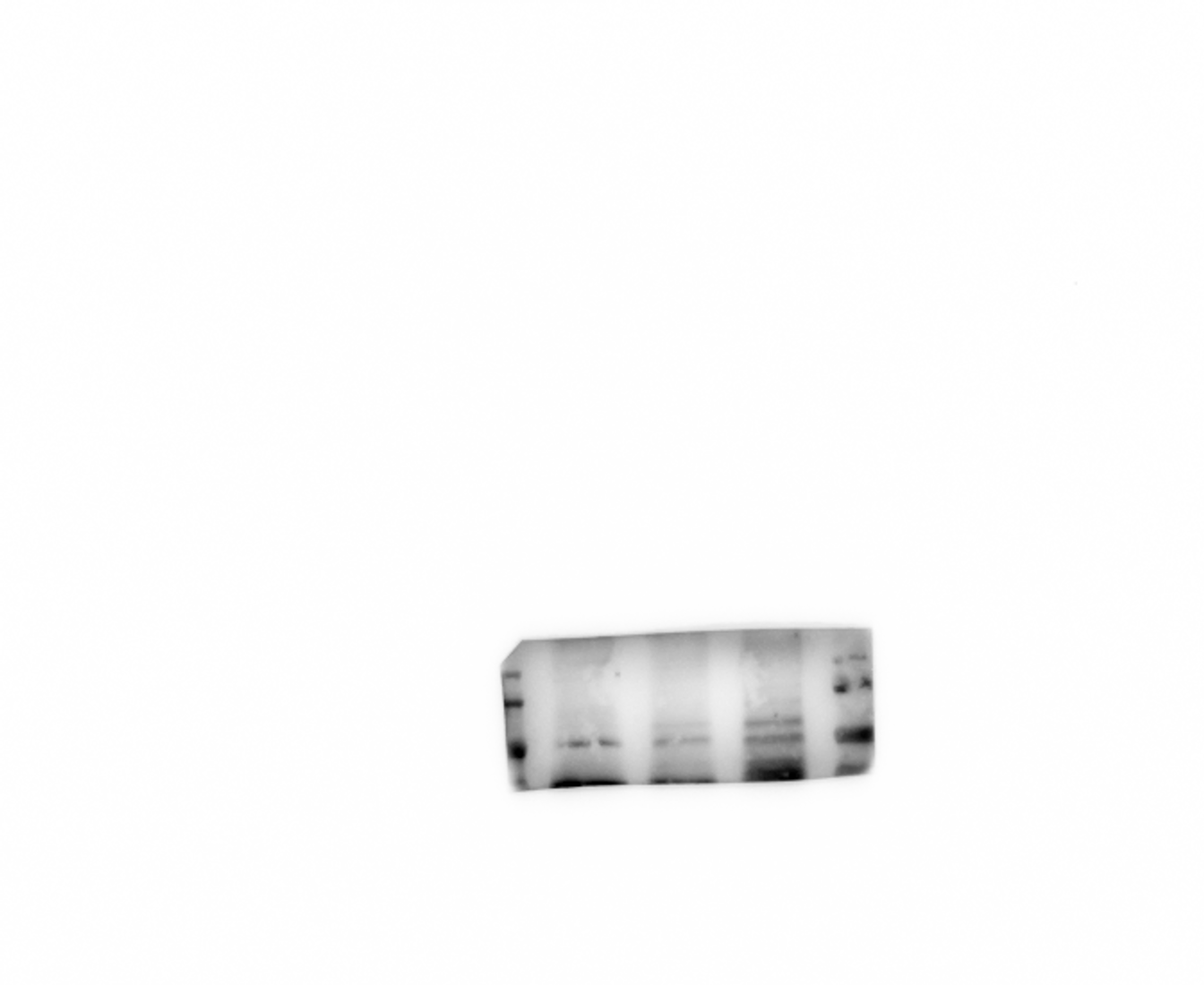

Supplement: Figure 1—source data 2. [file elife-82703-fig1-data2.zip › Figure 1 source data2/Figure 1E-YTHDC1-raw-inverted.Tif]

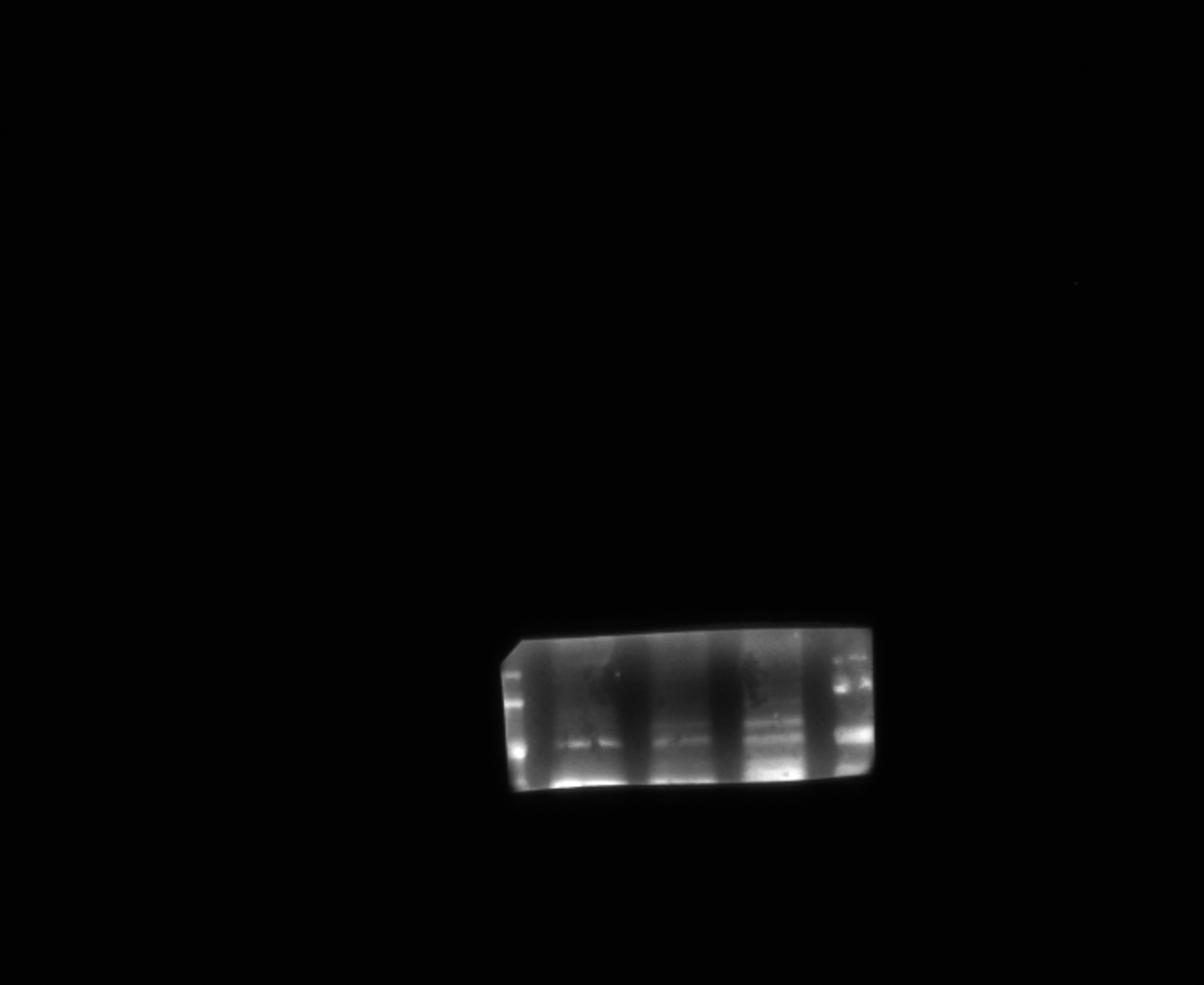

Supplement: Figure 1—source data 2. [file elife-82703-fig1-data2.zip › Figure 1 source data2/Figure 1E-YTHDC1-raw.Tif]

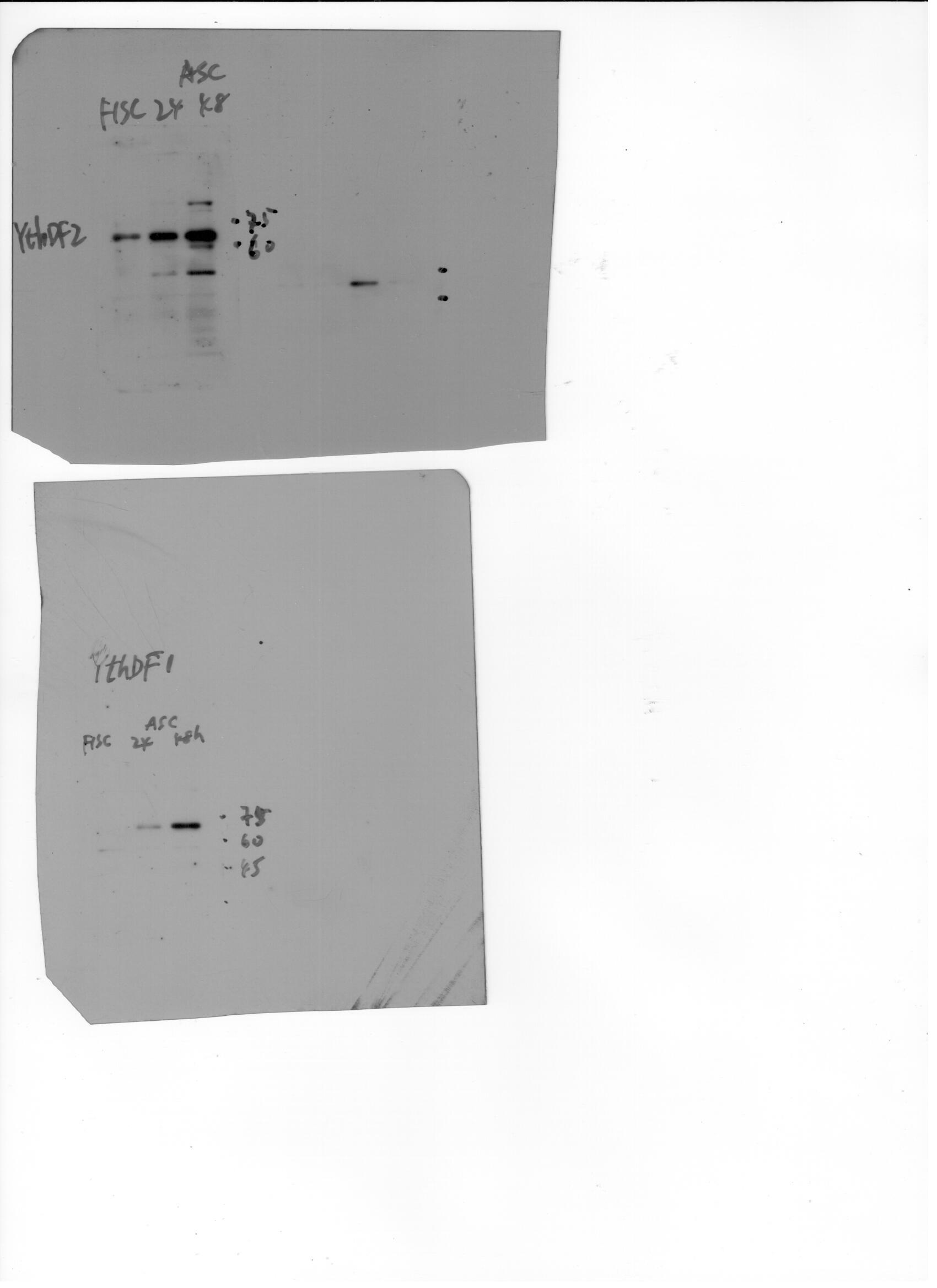

Supplement: Figure 1—source data 2. [file elife-82703-fig1-data2.zip › Figure 1 source data2/Figure 1E-YTHDF1 and YTHDF2-gary-scanned-raw.jpeg]

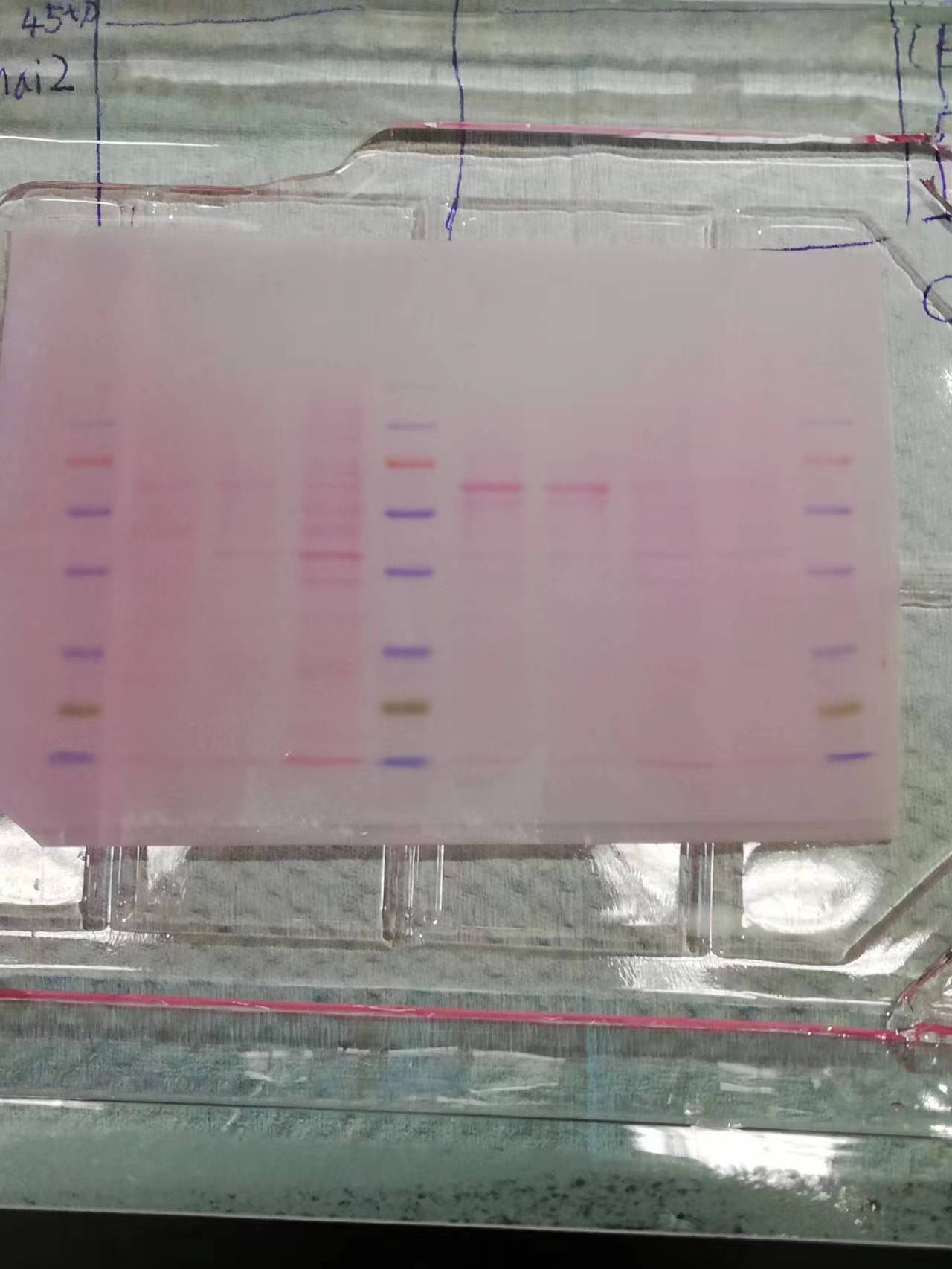

Supplement: Figure 1—source data 2. [file elife-82703-fig1-data2.zip › Figure 1 source data2/Figure 1E-YTHDF1 and YTHDF2-loading control-photo-raw.jpg]

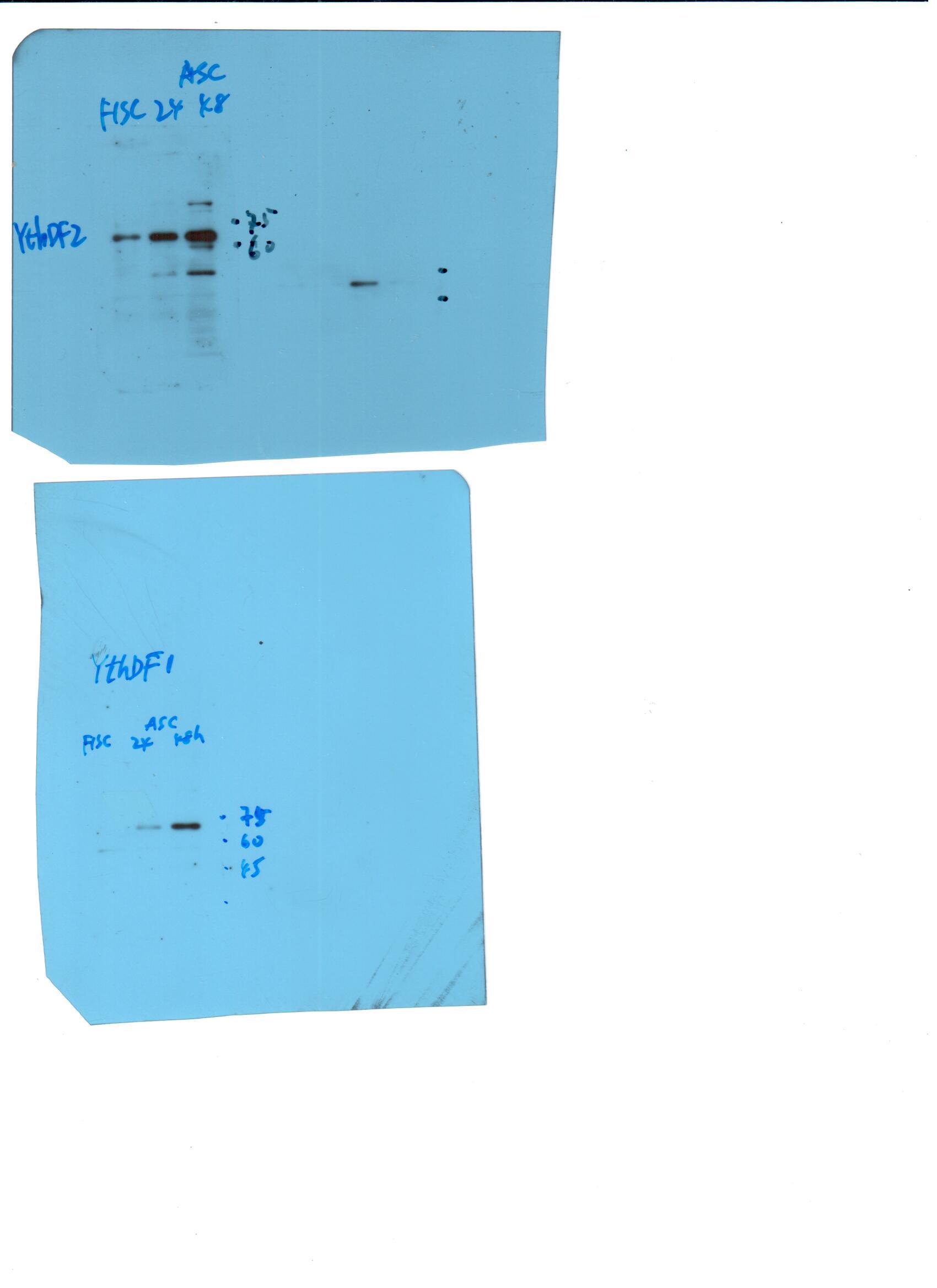

Supplement: Figure 1—source data 2. [file elife-82703-fig1-data2.zip › Figure 1 source data2/Figure 1E-YTHDF1 and YTHDF2-scanned-raw.jpeg]

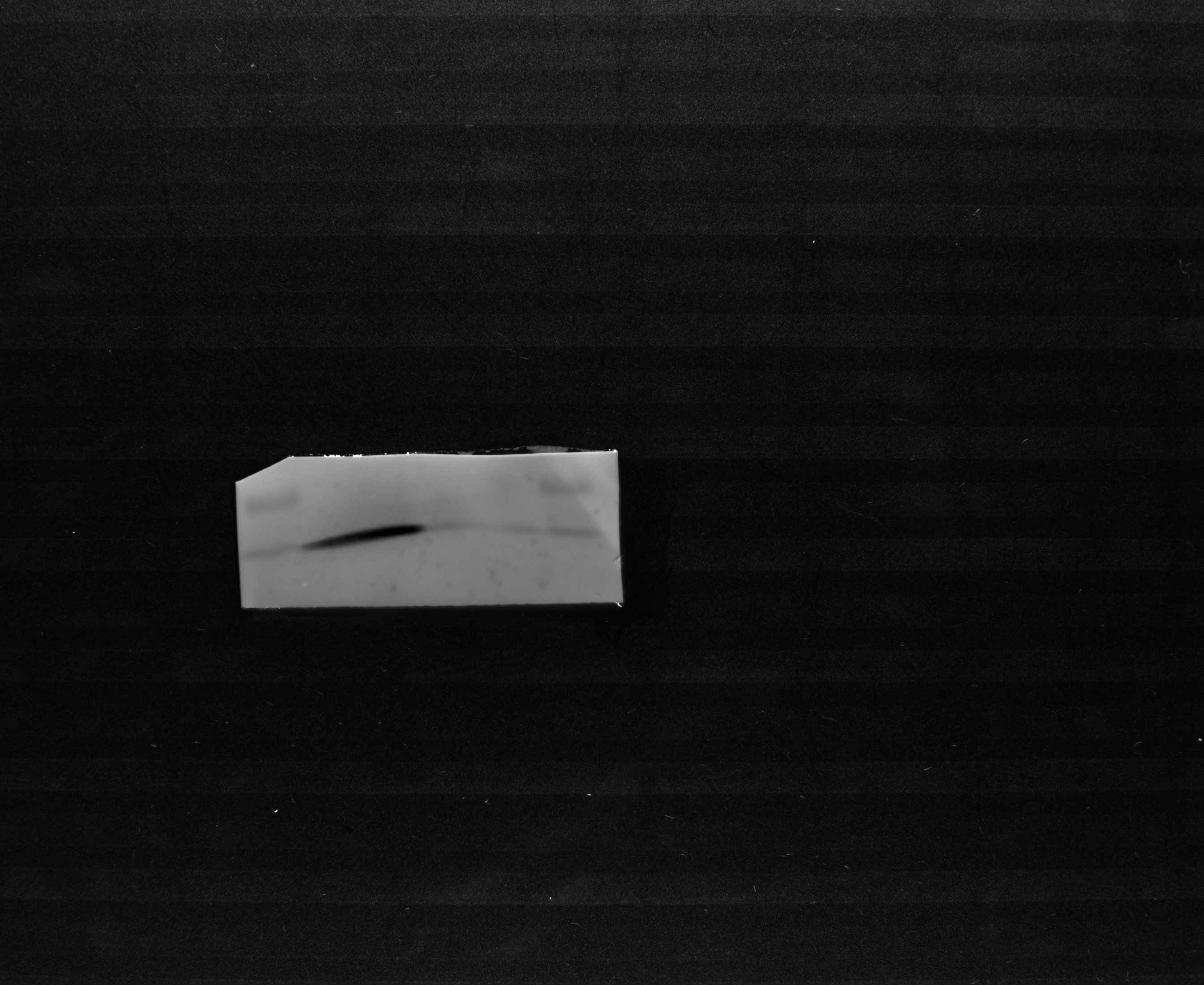

Supplement: Figure 1—source data 2. [file elife-82703-fig1-data2.zip › Figure 1 source data2/Figure 1G-Histone H3-merged with marker.jpg]

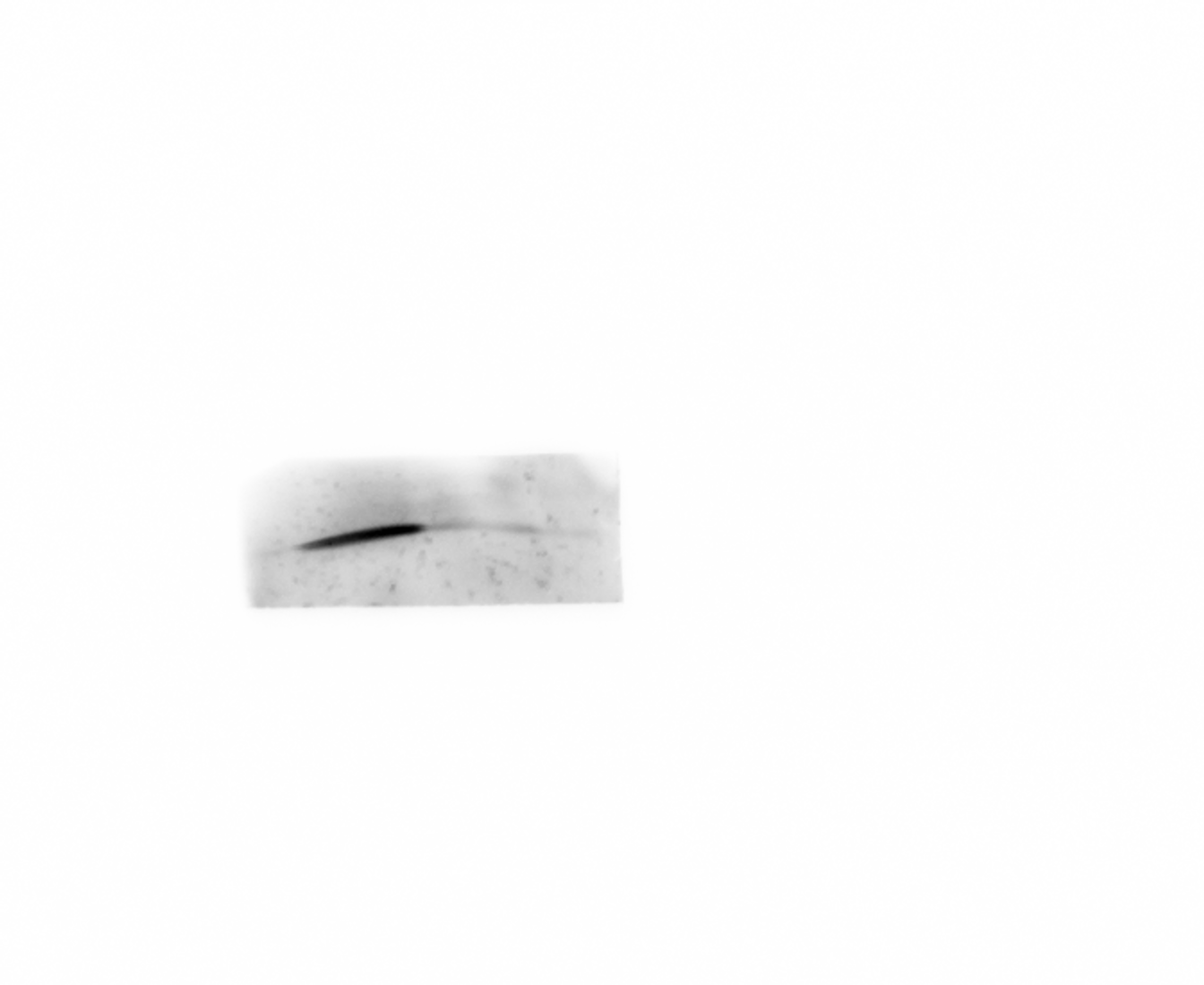

Supplement: Figure 1—source data 2. [file elife-82703-fig1-data2.zip › Figure 1 source data2/Figure 1G-Histone H3-raw-inverted.Tif]

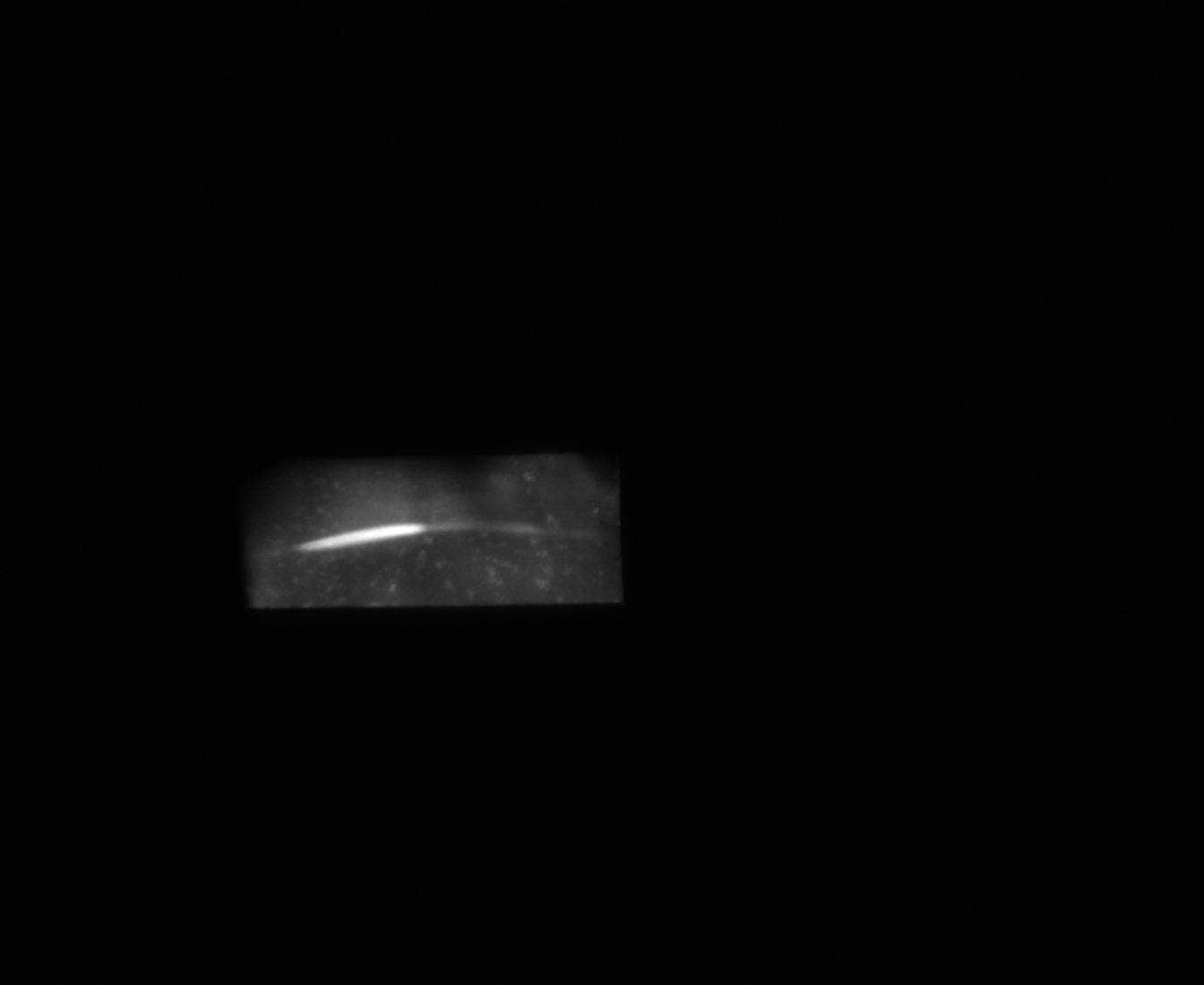

Supplement: Figure 1—source data 2. [file elife-82703-fig1-data2.zip › Figure 1 source data2/Figure 1G-Histone H3-raw.Tif]

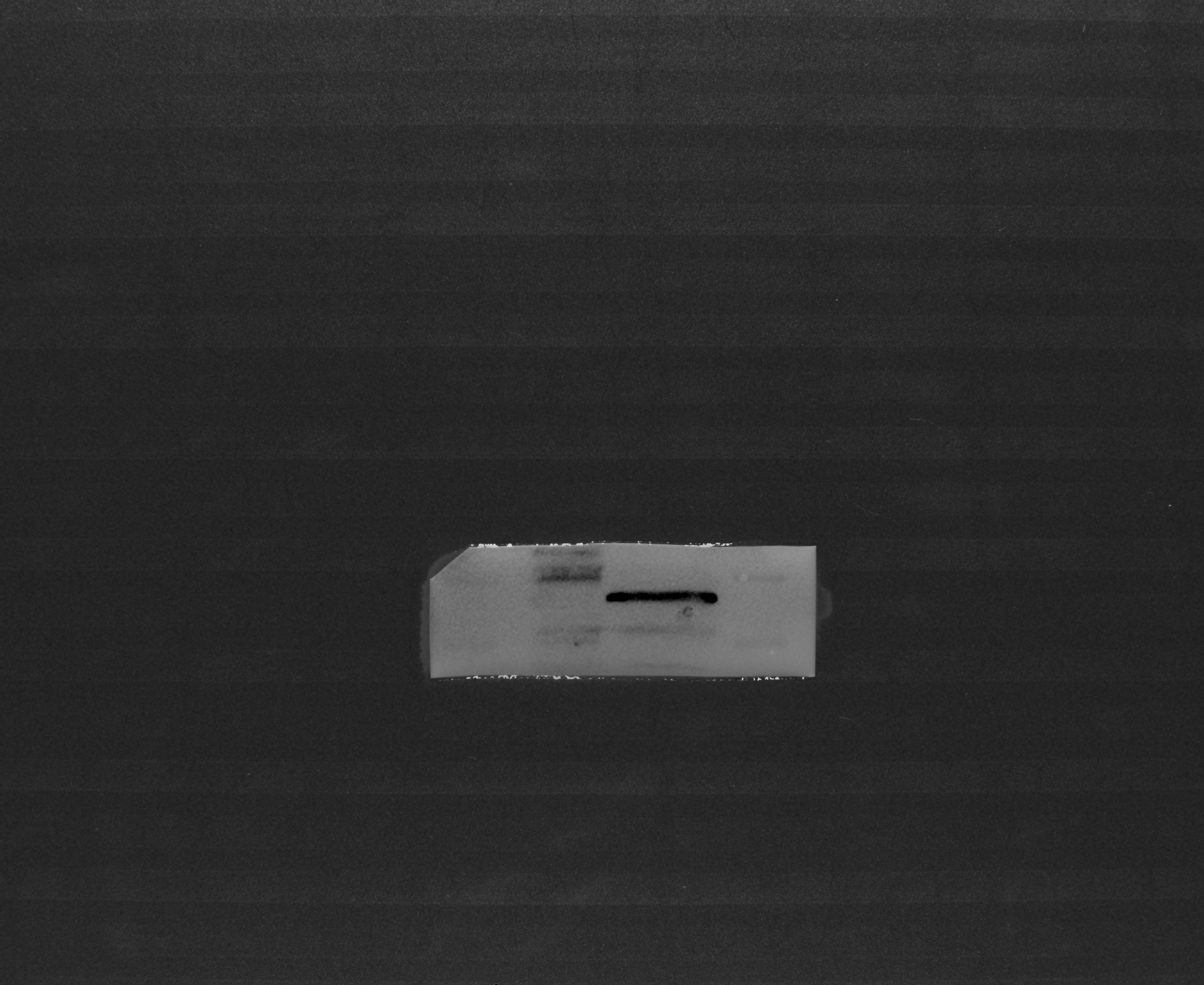

Supplement: Figure 1—source data 2. [file elife-82703-fig1-data2.zip › Figure 1 source data2/Figure 1G-tublin-merged with marker.jpg]

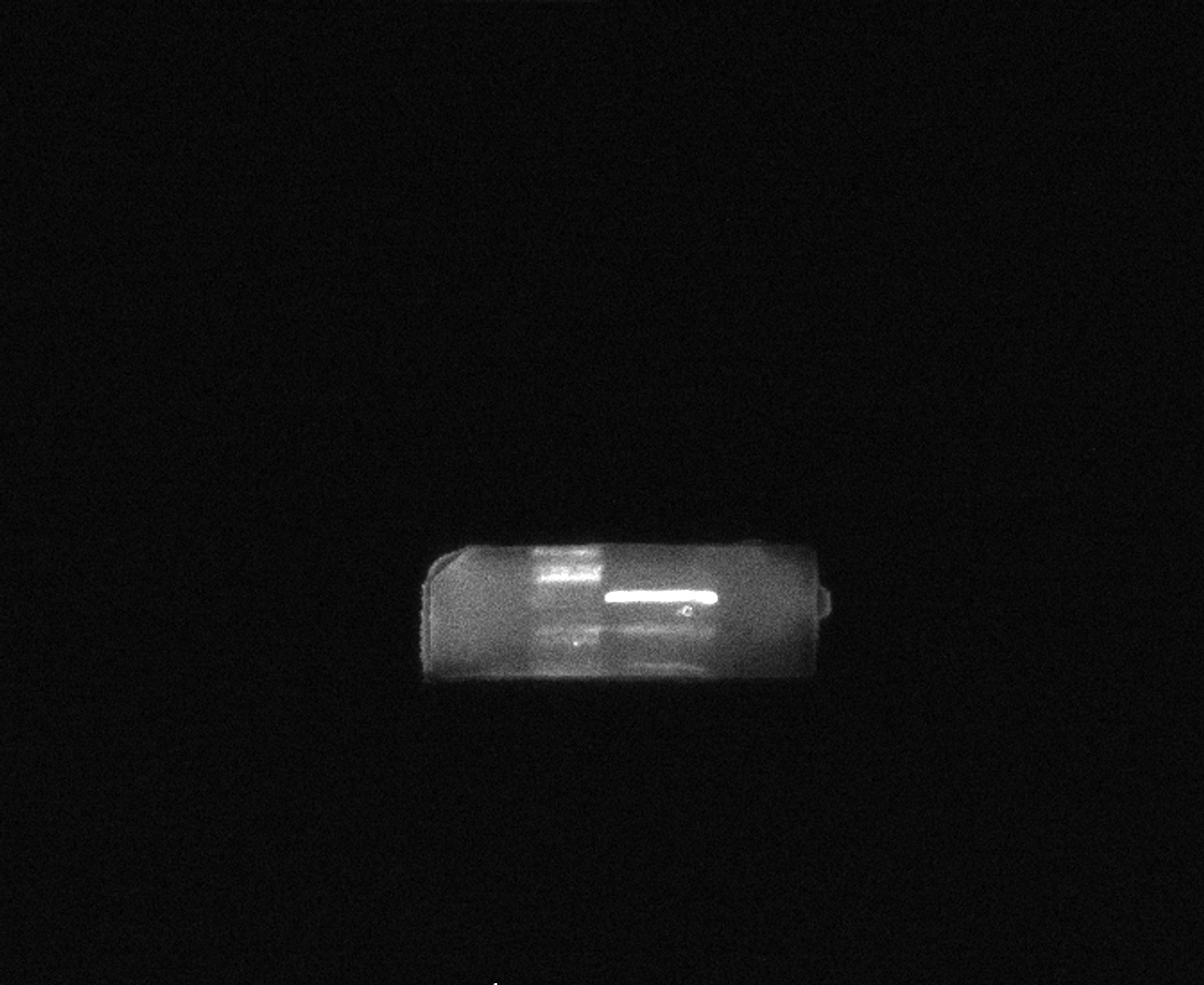

Supplement: Figure 1—source data 2. [file elife-82703-fig1-data2.zip › Figure 1 source data2/Figure 1G-tublin-raw.Tif]

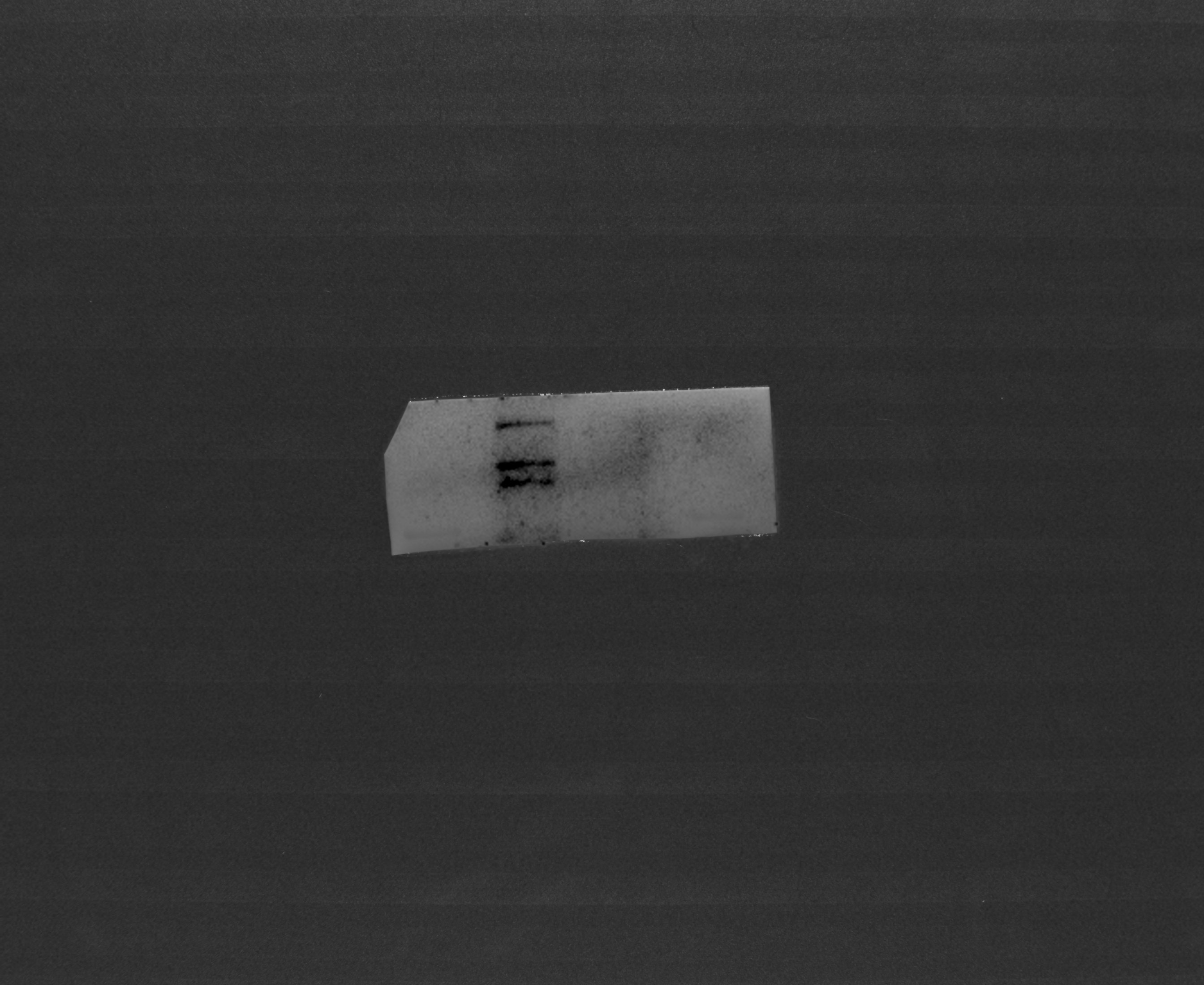

Supplement: Figure 1—source data 2. [file elife-82703-fig1-data2.zip › Figure 1 source data2/Figure 1G-YTHDC1-merged with marker.jpg]

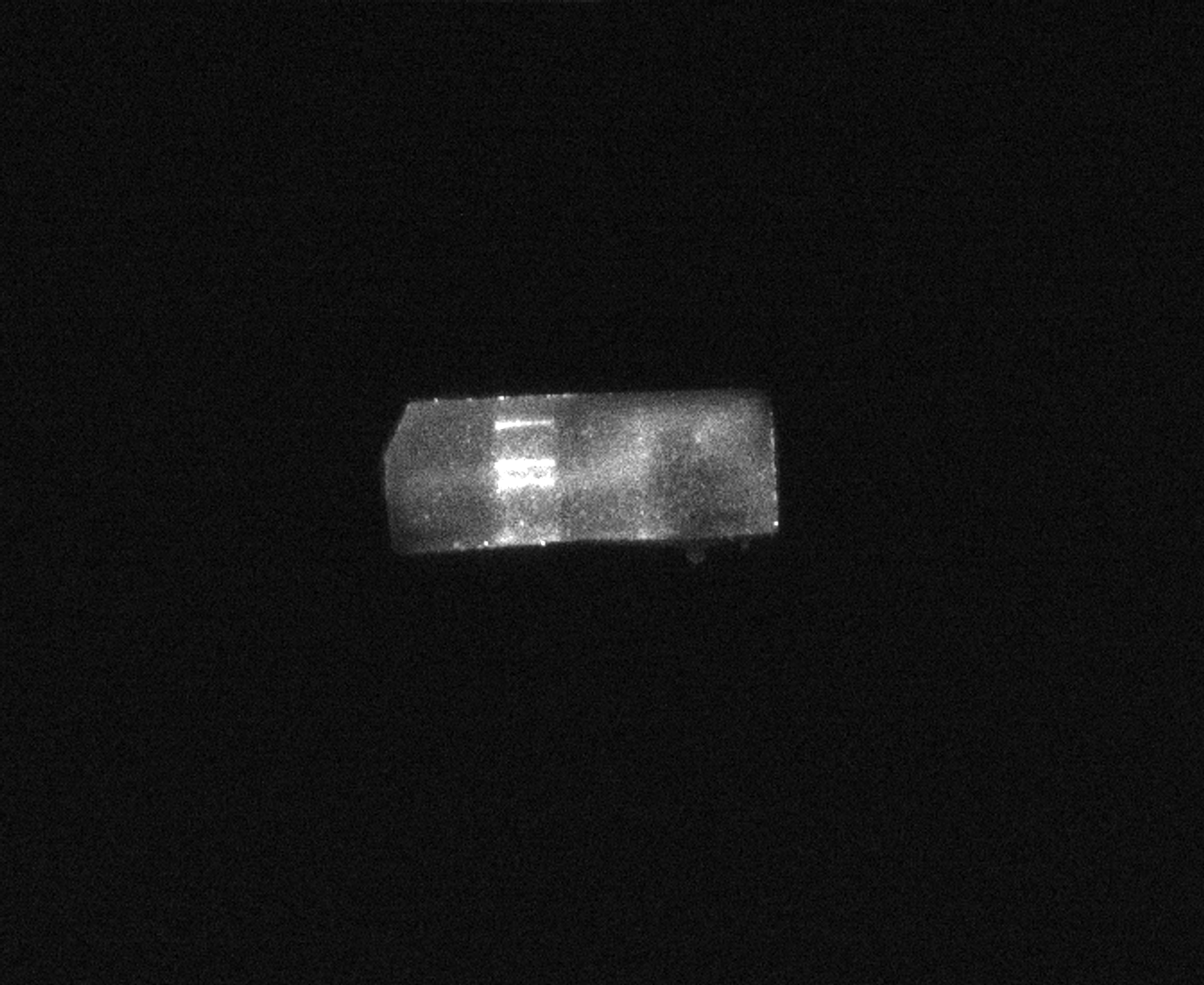

Supplement: Figure 1—source data 2. [file elife-82703-fig1-data2.zip › Figure 1 source data2/Figure 1G-YTHDC1-raw.Tif]

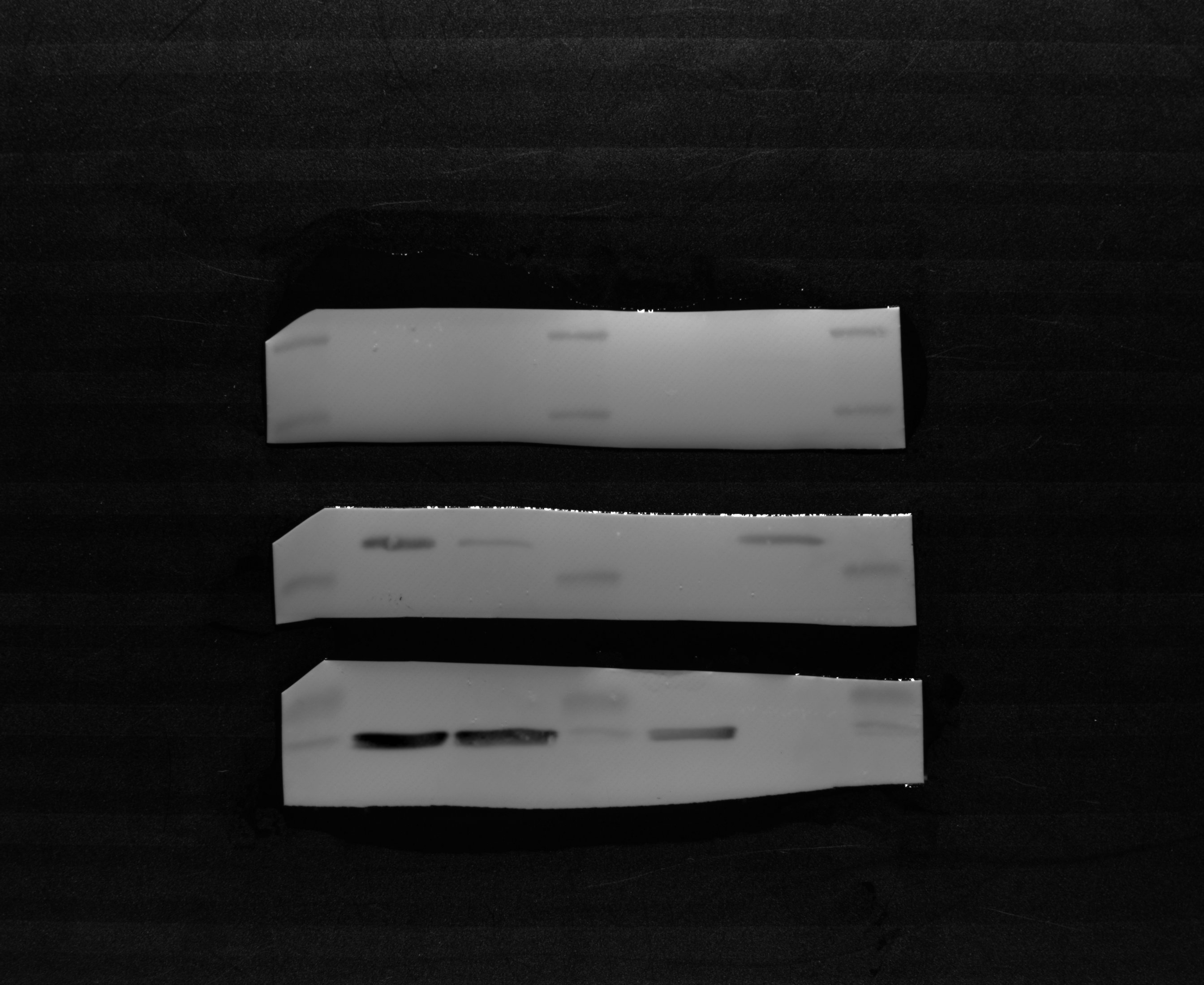

Supplement: Figure 2—source data 1. [file elife-82703-fig2-data1.zip › figure 2 source data1/Figure 2C-Histone H3-merged with marker.jpg]

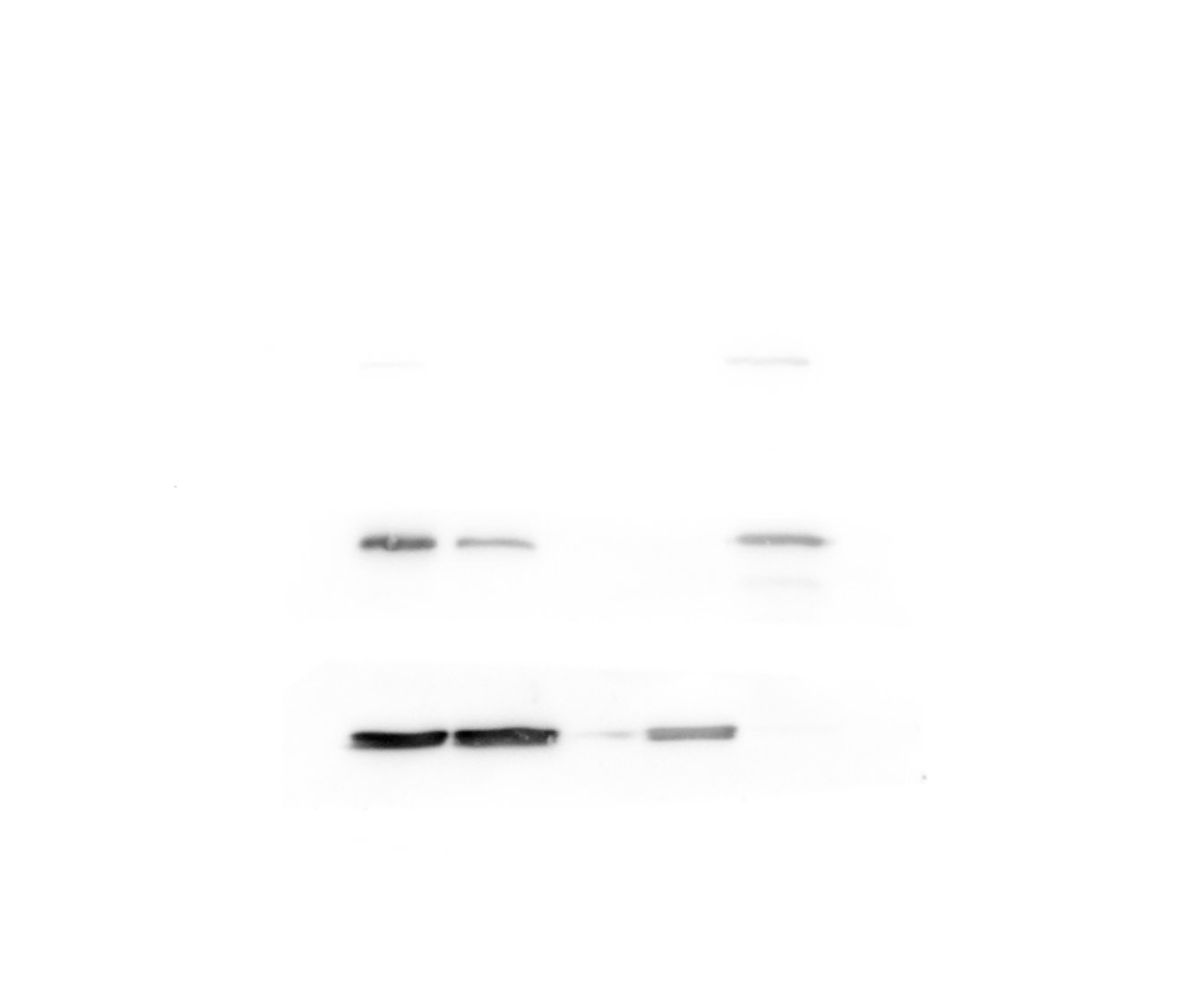

Supplement: Figure 2—source data 1. [file elife-82703-fig2-data1.zip › figure 2 source data1/Figure 2C-Histone H3-raw-inverted.jpg]

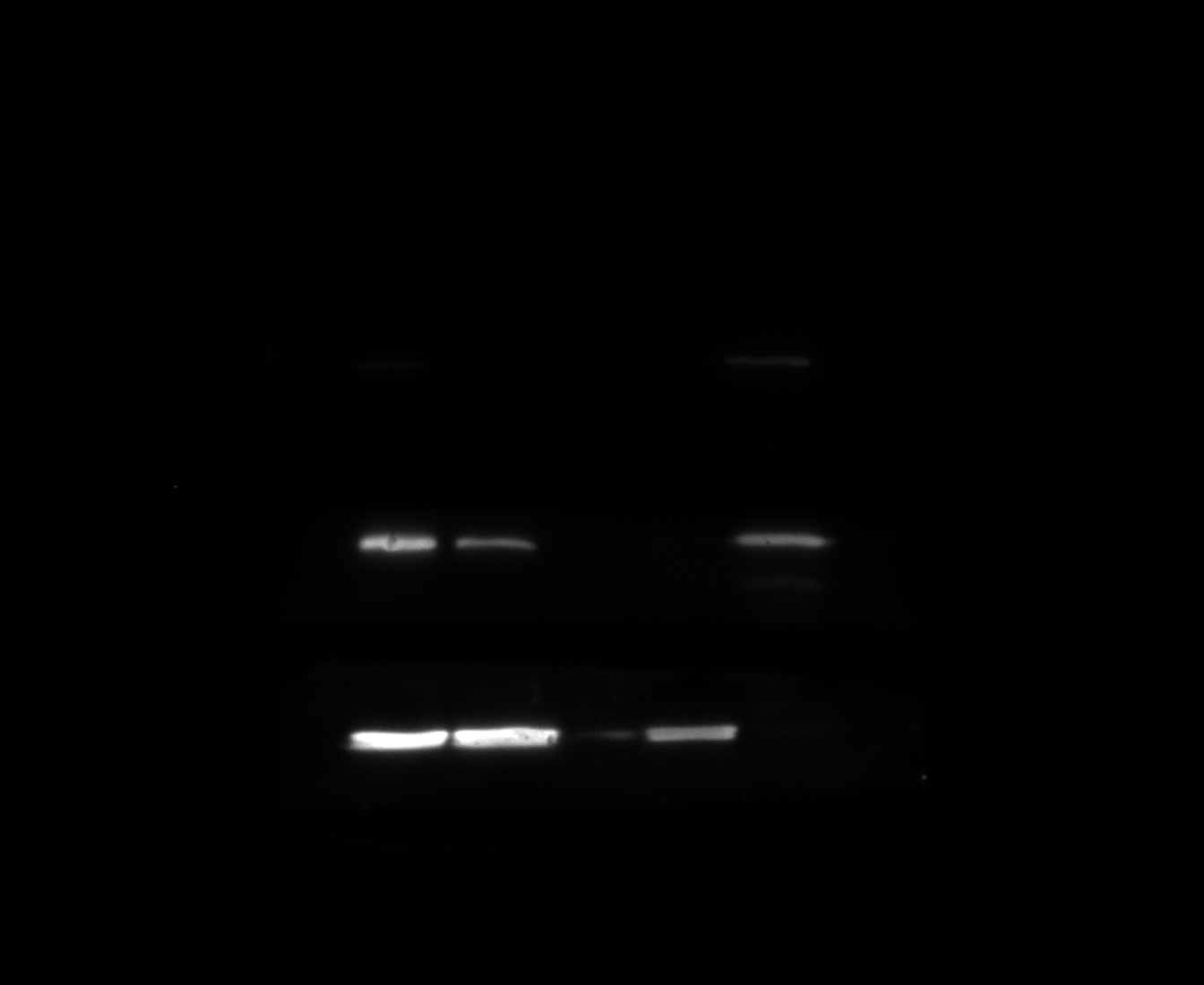

Supplement: Figure 2—source data 1. [file elife-82703-fig2-data1.zip › figure 2 source data1/Figure 2C-Histone H3-raw.Tif]

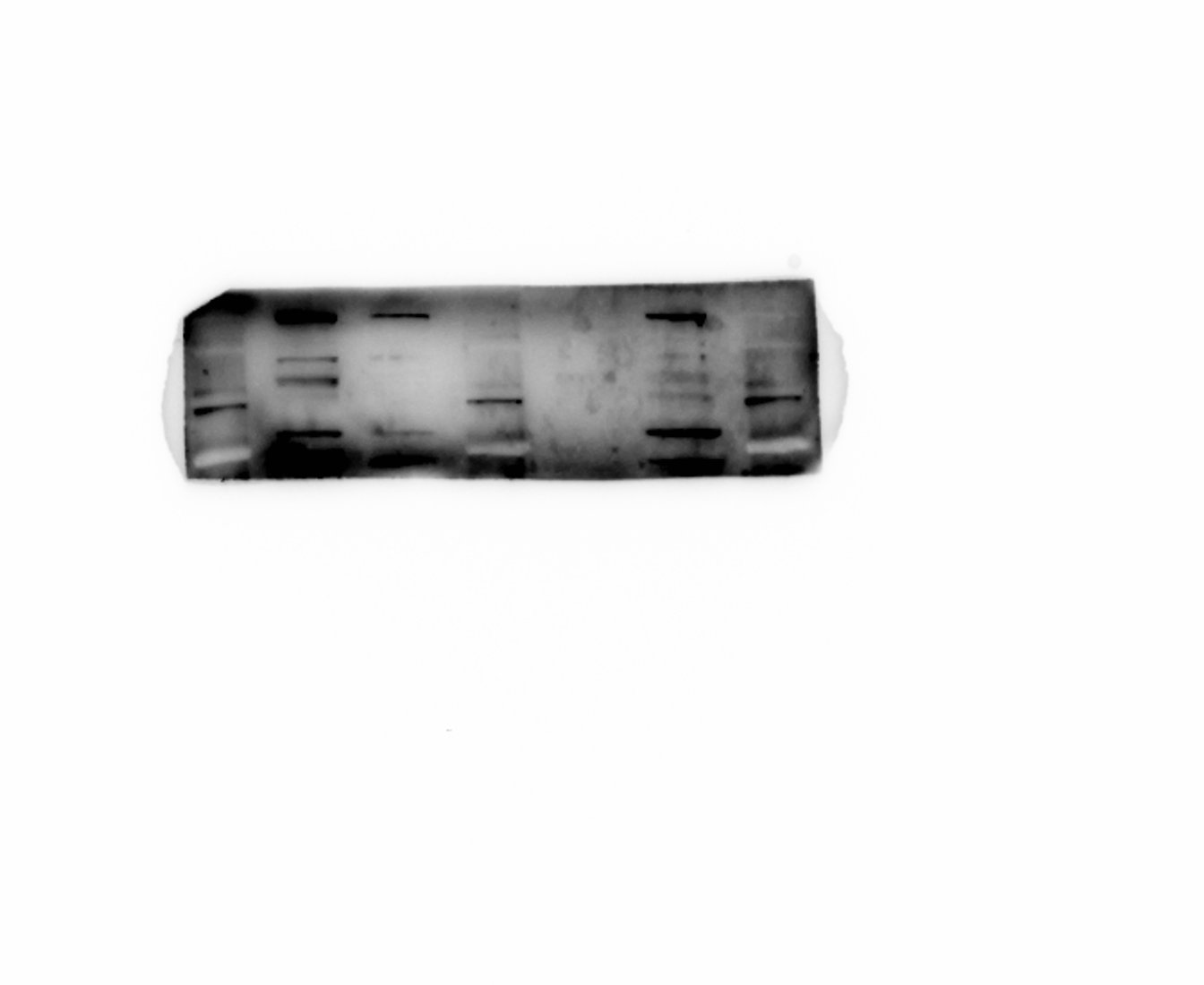

Supplement: Figure 2—source data 1. [file elife-82703-fig2-data1.zip › figure 2 source data1/Figure 2C-YTHDC1-raw-inverted.tif]

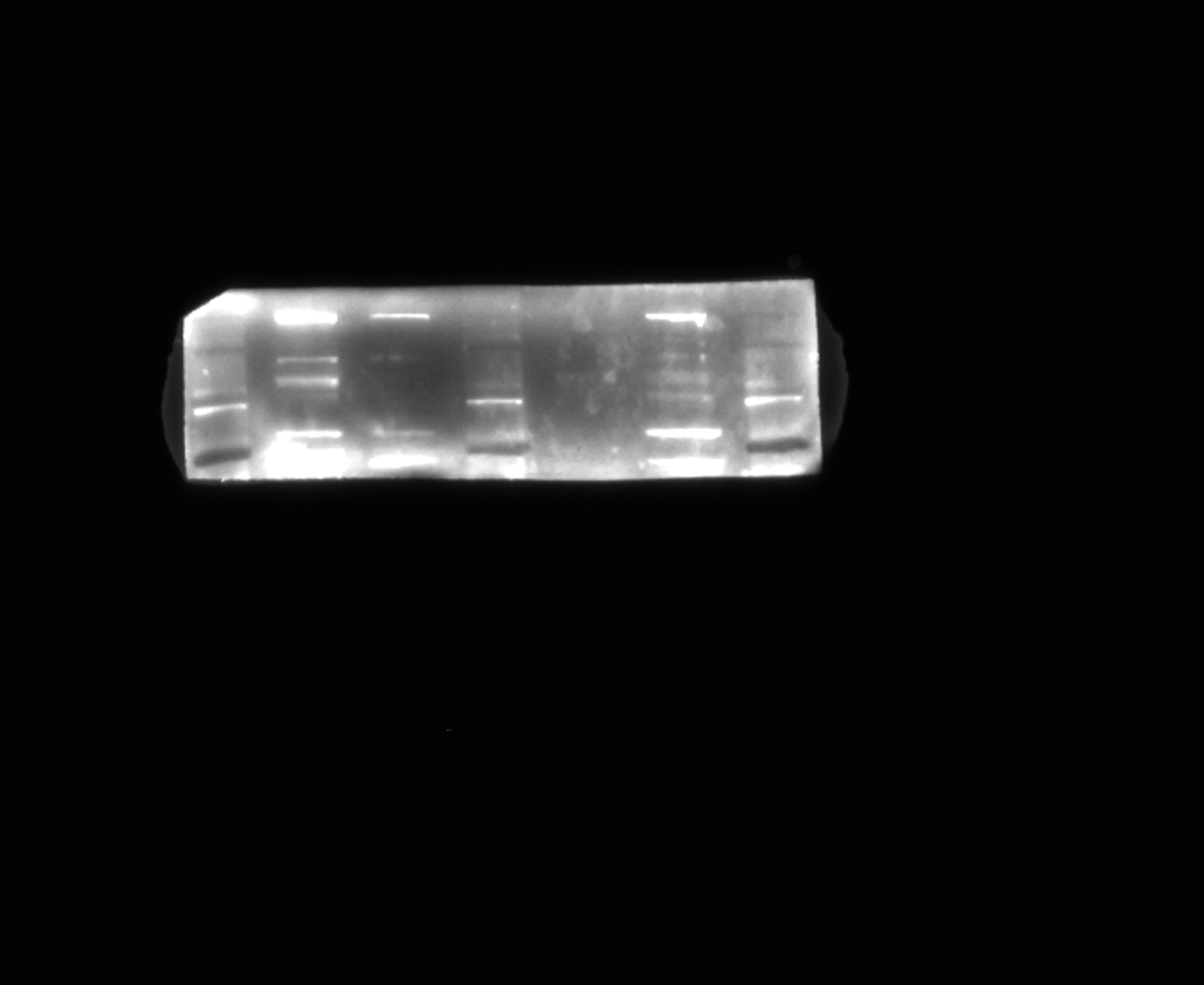

Supplement: Figure 2—source data 1. [file elife-82703-fig2-data1.zip › figure 2 source data1/Figure 2C-YTHDC1-raw.Tif]

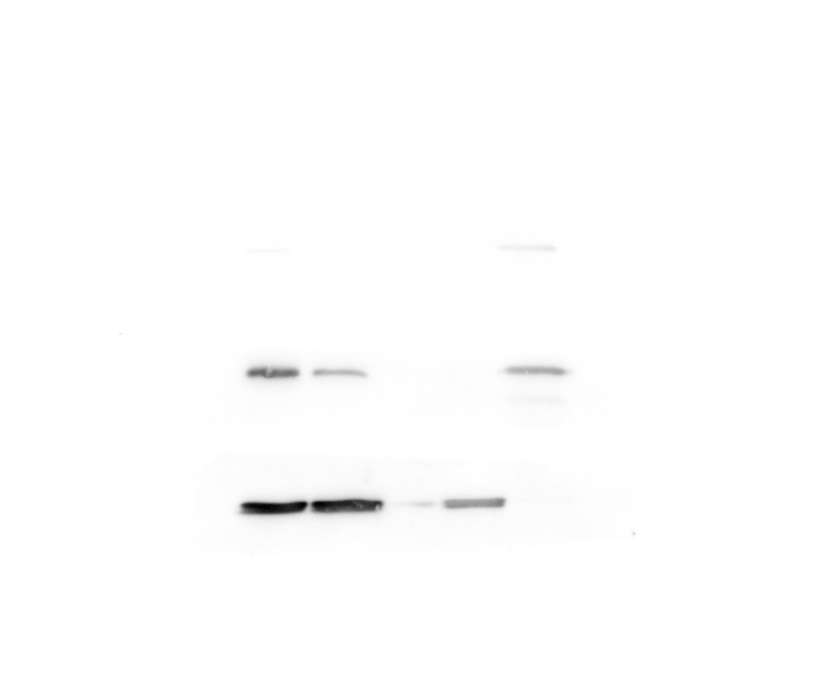
Figure 2C-Hisonte H3

**15kDa**

**Histone H3**

**Ctrl iKO**

**Ctrl iKO**

**Histone H3**


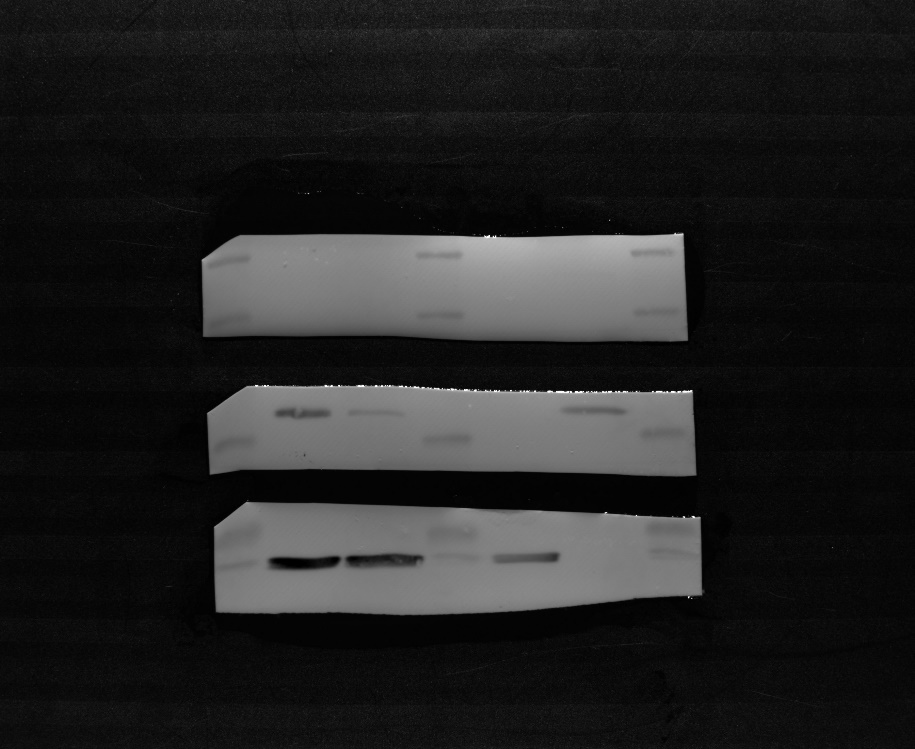


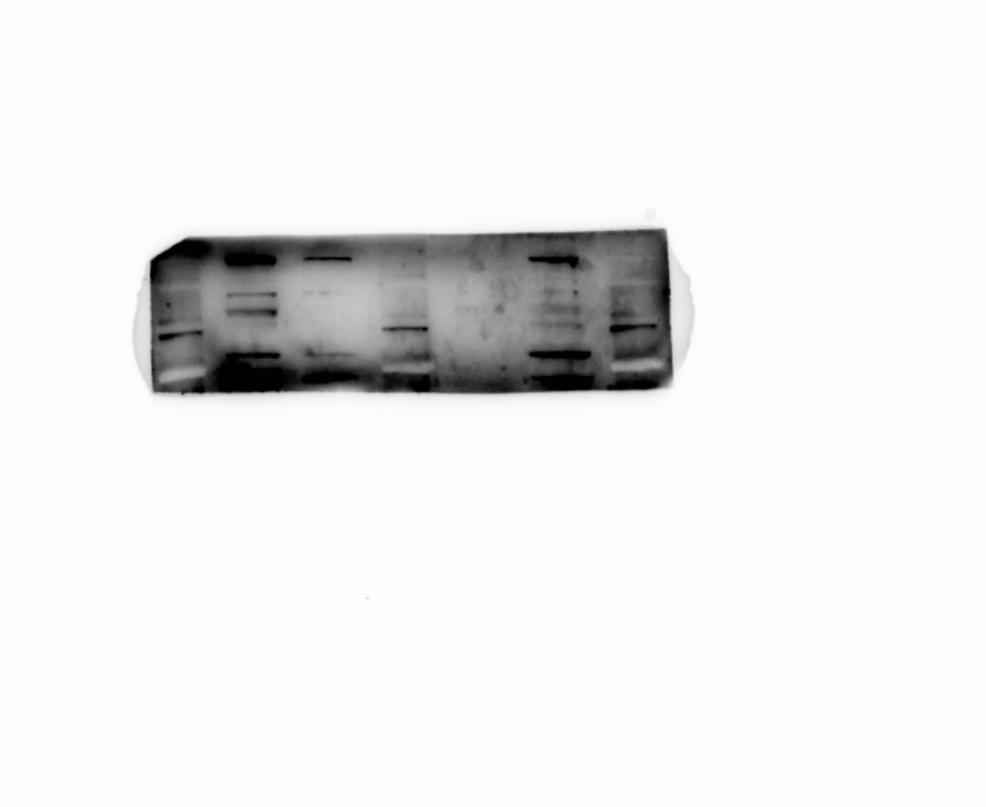
Figure 2C-YTHDC1

**100kDa**

**YTHDC1**

**Ctrl iKO**

Supplement: Figure 2—source data 1. [file elife-82703-fig2-data1.zip › figure 2 source data1/Figure 2C-with all relevant bands labelled.docx]

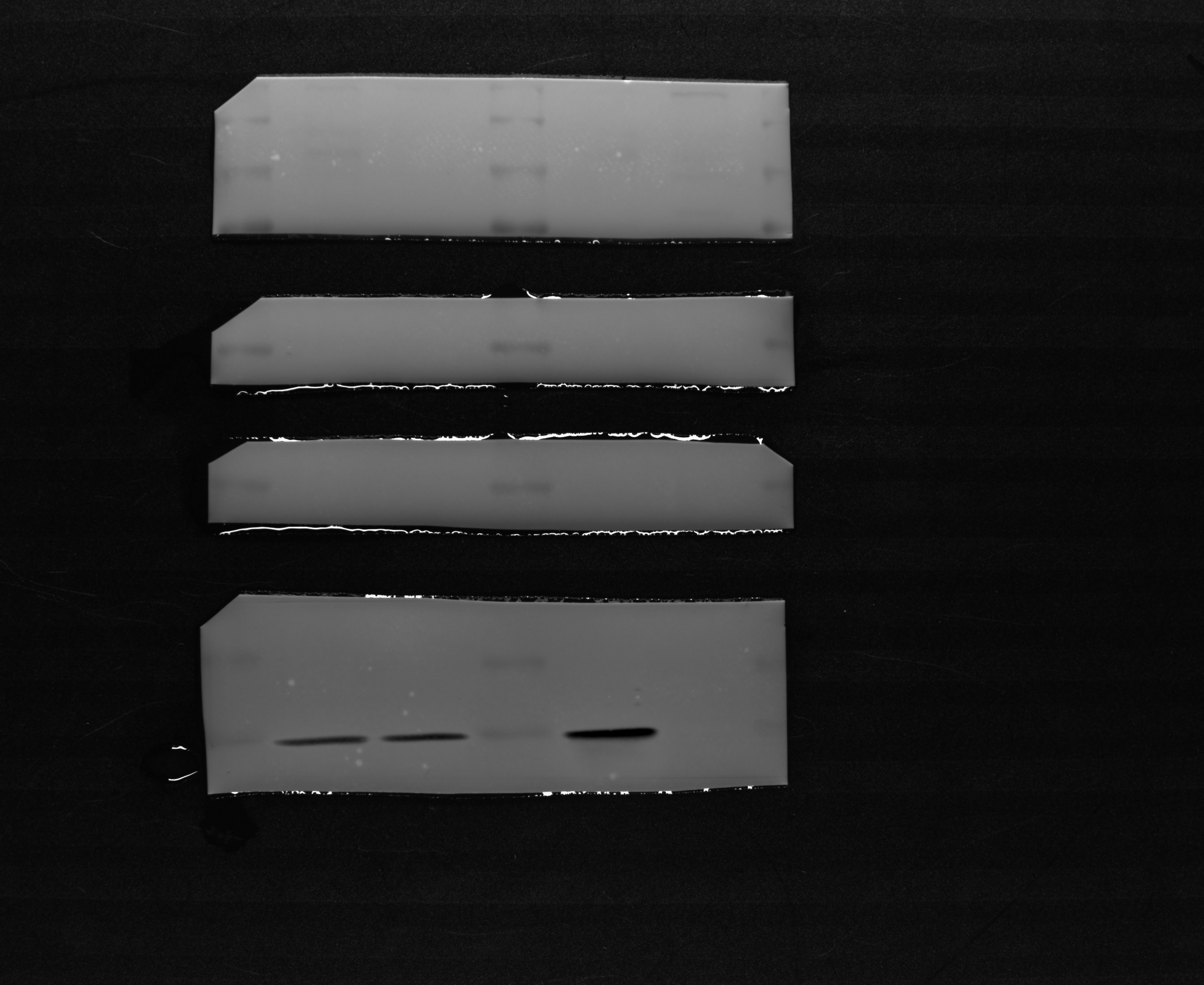

Supplement: Figure 3—figure supplement 1—source data 1. [file elife-82703-fig3-figsupp1-data1.zip › Figure 3-figure supplement 1-Source data 1/Figure 3 source data1/Figure 3-supplement-1C/Figure 3-supplement-1C-Histone H3-merged with marker.jpg]

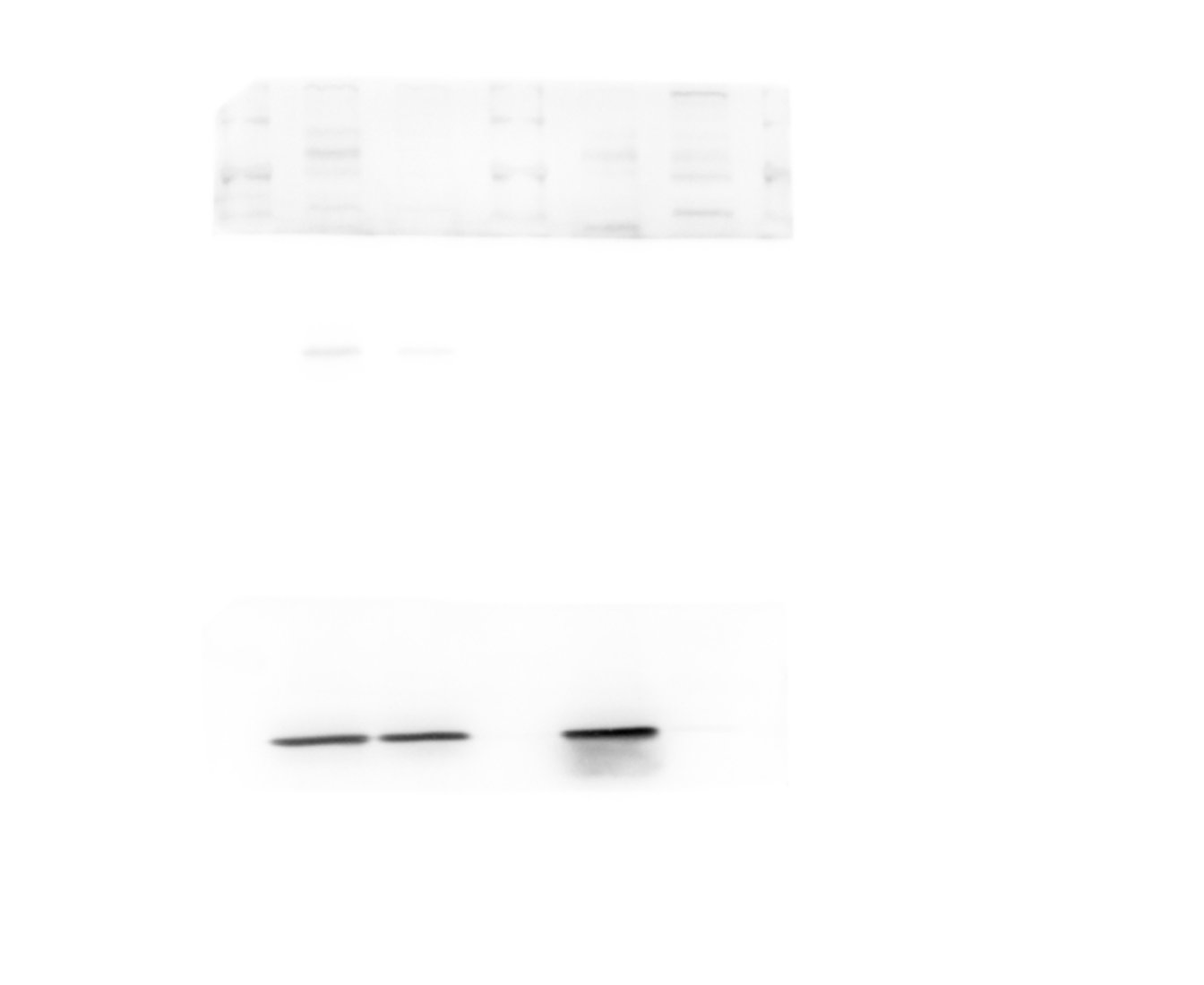

Supplement: Figure 3—figure supplement 1—source data 1. [file elife-82703-fig3-figsupp1-data1.zip › Figure 3-figure supplement 1-Source data 1/Figure 3 source data1/Figure 3-supplement-1C/Figure 3-supplement-1C-Histone H3-raw-inverted.jpg]

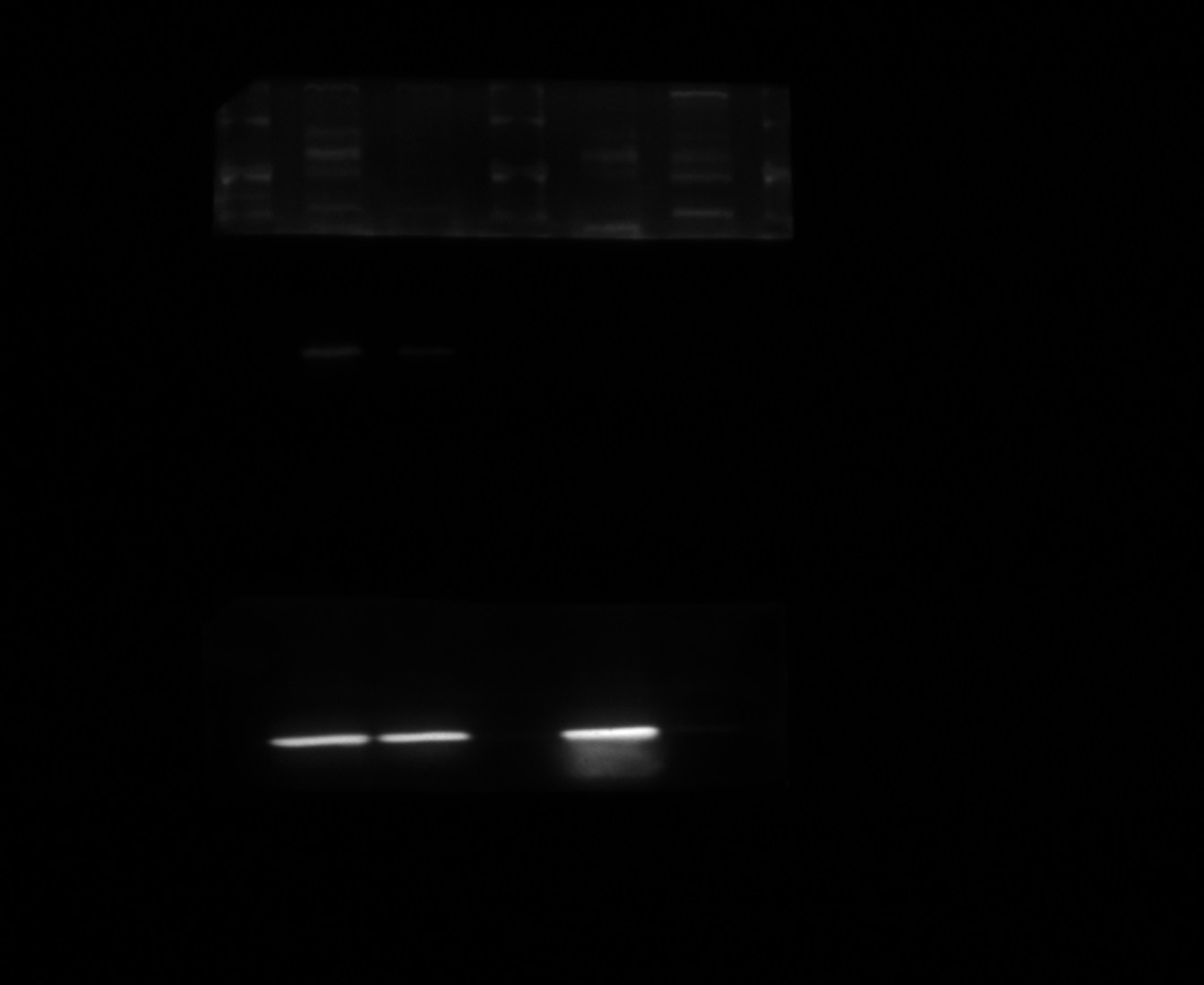

Supplement: Figure 3—figure supplement 1—source data 1. [file elife-82703-fig3-figsupp1-data1.zip › Figure 3-figure supplement 1-Source data 1/Figure 3 source data1/Figure 3-supplement-1C/Figure 3-supplement-1C-Histone H3-raw.Tif]

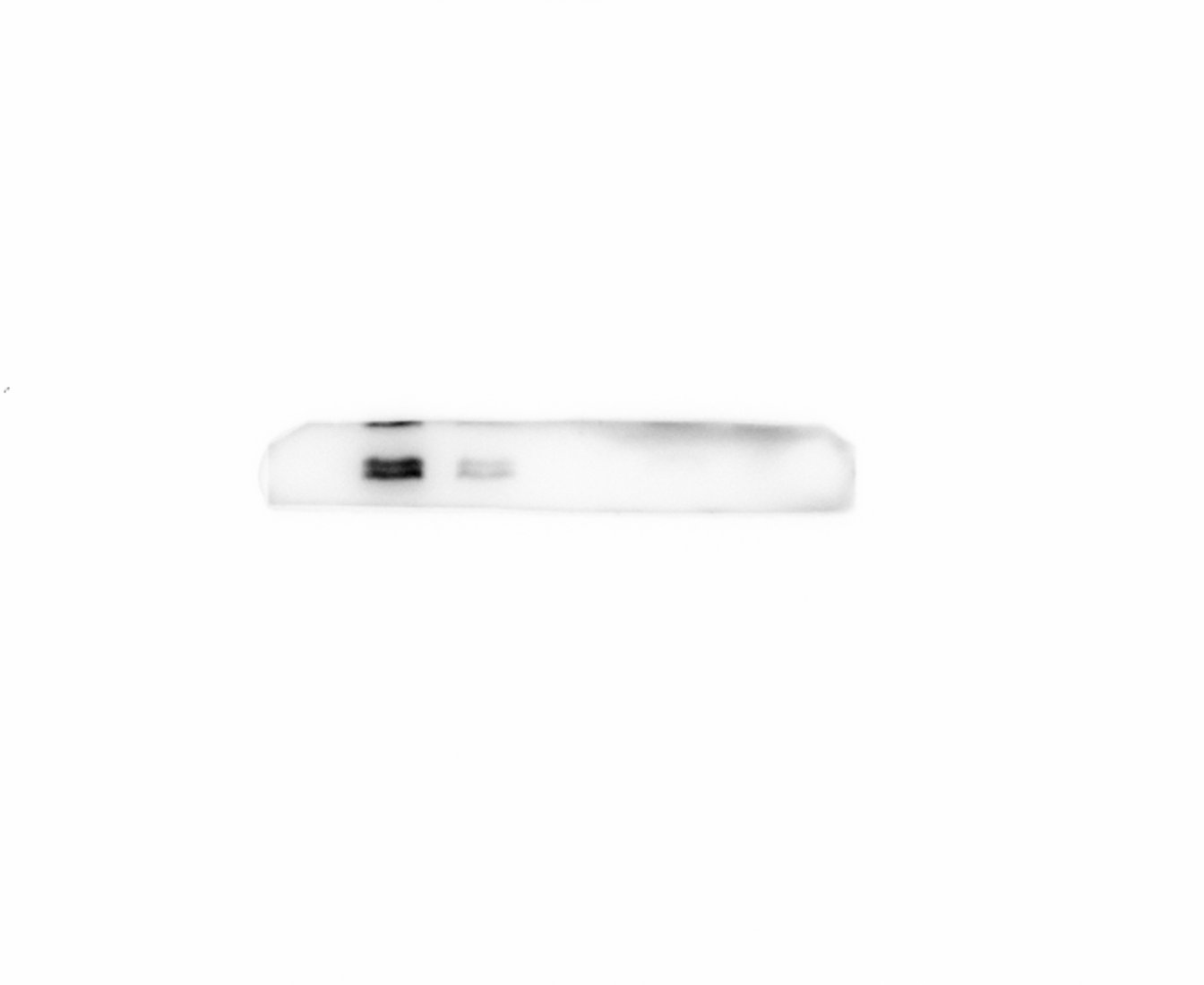

Supplement: Figure 3—figure supplement 1—source data 1. [file elife-82703-fig3-figsupp1-data1.zip › Figure 3-figure supplement 1-Source data 1/Figure 3 source data1/Figure 3-supplement-1C/Figure 3-supplement-1C-Myod-raw-inverted.jpg]

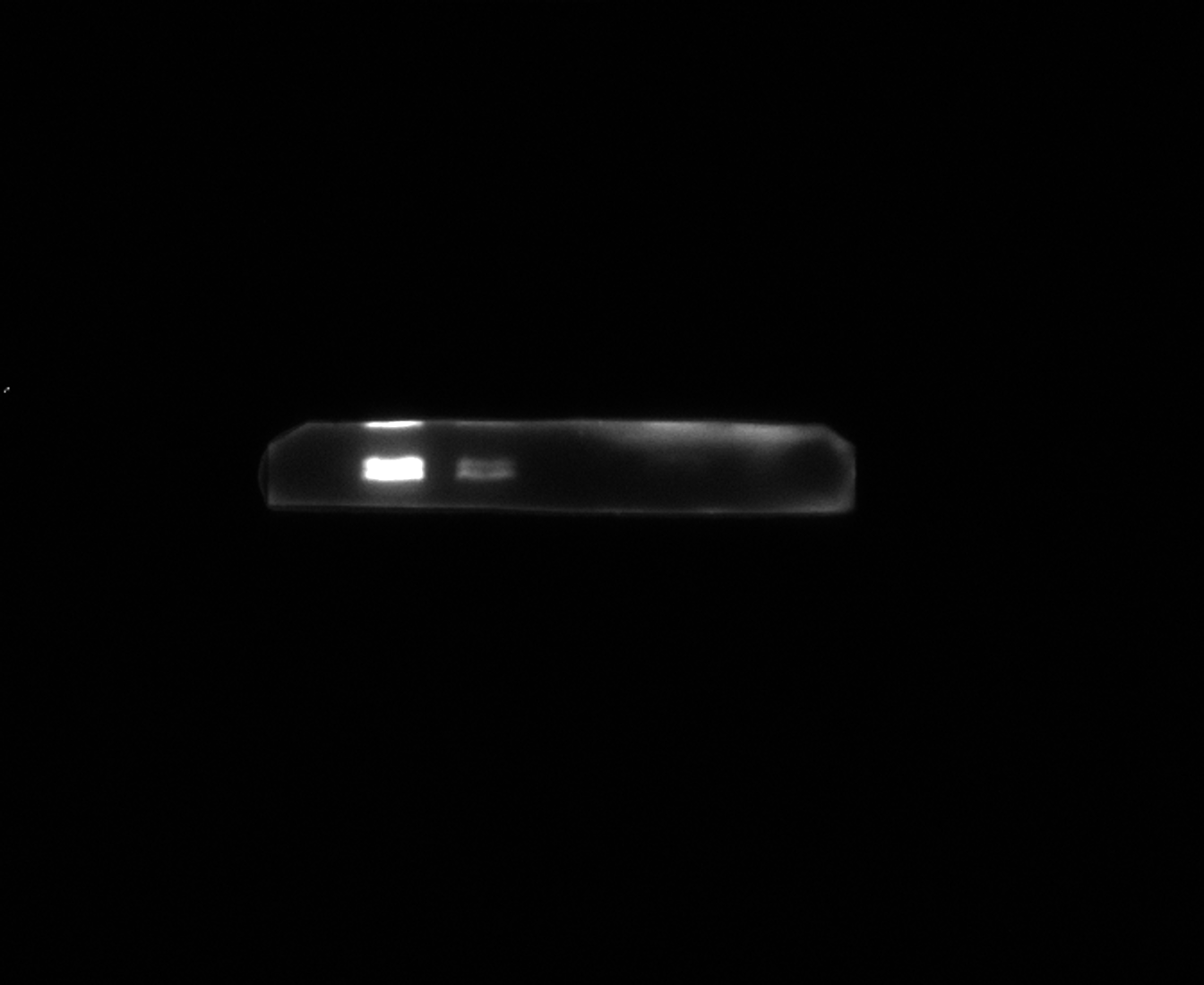

Supplement: Figure 3—figure supplement 1—source data 1. [file elife-82703-fig3-figsupp1-data1.zip › Figure 3-figure supplement 1-Source data 1/Figure 3 source data1/Figure 3-supplement-1C/Figure 3-supplement-1C-Myod-raw.Tif]

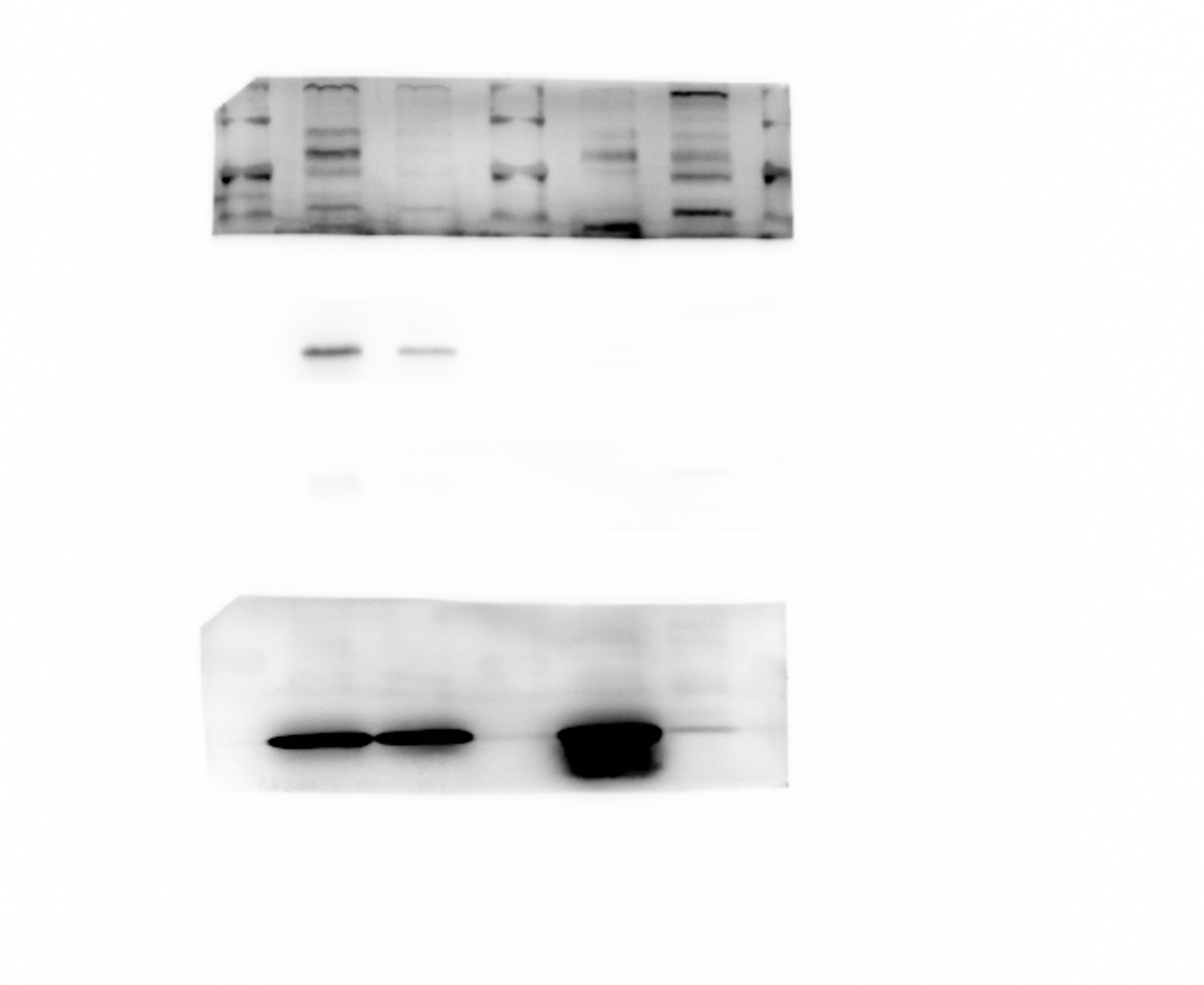

Supplement: Figure 3—figure supplement 1—source data 1. [file elife-82703-fig3-figsupp1-data1.zip › Figure 3-figure supplement 1-Source data 1/Figure 3 source data1/Figure 3-supplement-1C/Figure 3-supplement-1C-YTHDC1 and Pax7-raw-inverted.tif]

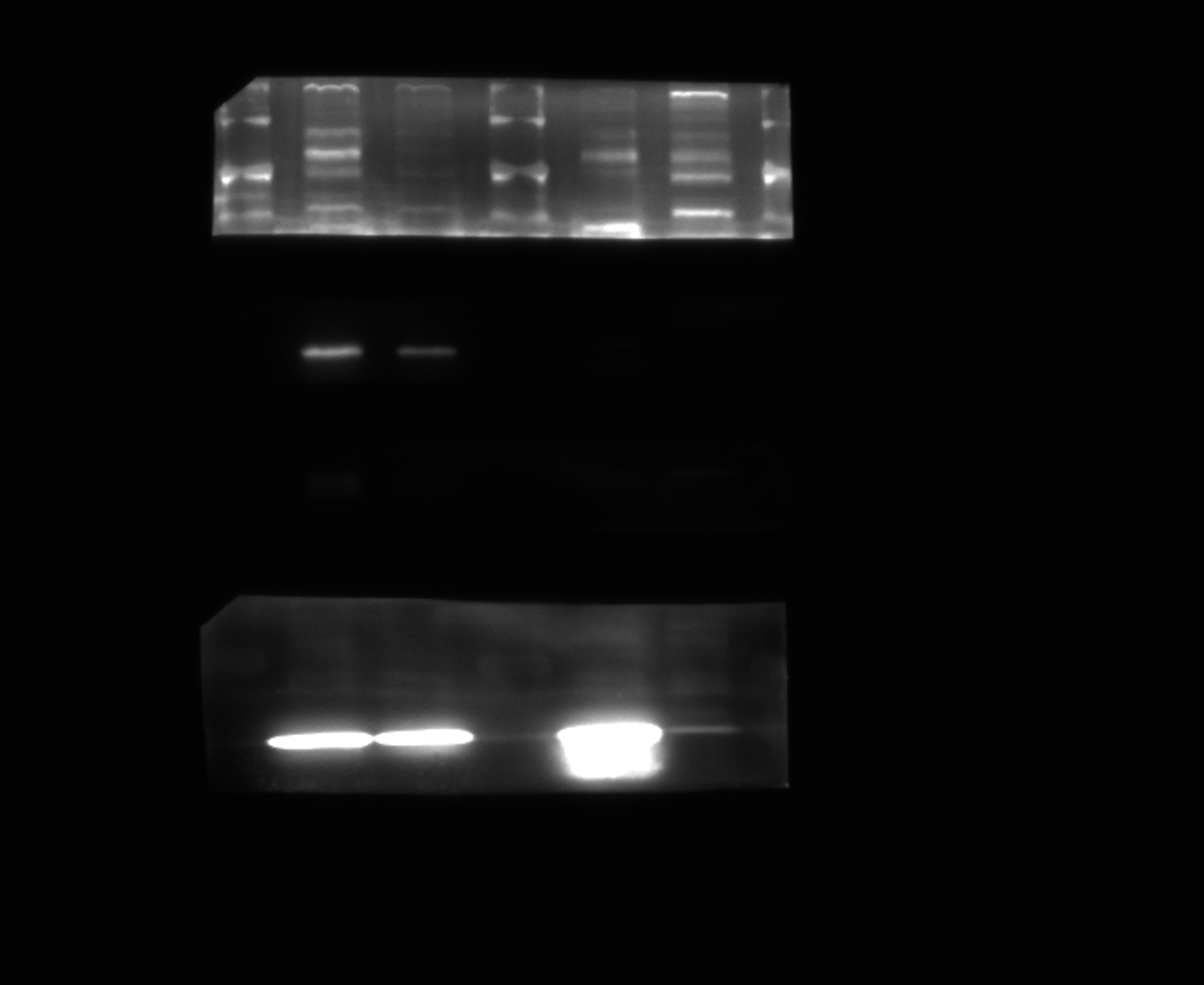

Supplement: Figure 3—figure supplement 1—source data 1. [file elife-82703-fig3-figsupp1-data1.zip › Figure 3-figure supplement 1-Source data 1/Figure 3 source data1/Figure 3-supplement-1C/Figure 3-supplement-1C-YTHDC1 and Pax7-raw.Tif]

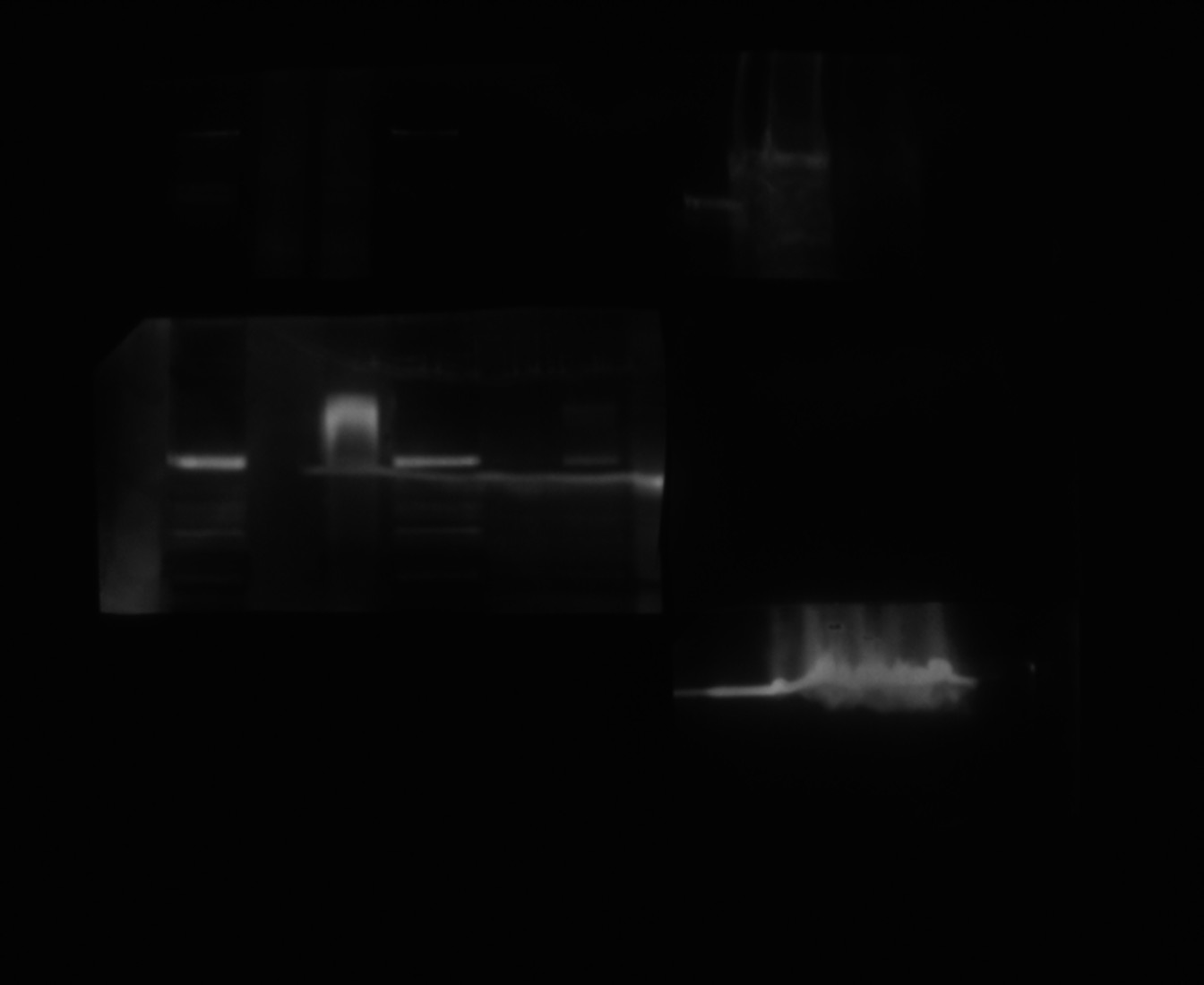

Supplement: Figure 3—figure supplement 1—source data 1. [file elife-82703-fig3-figsupp1-data1.zip › Figure 3-figure supplement 1-Source data 1/Figure 3 source data1/Figure 3-supplement-1E/Figure 3-supplement-1E-clone1-Histone H3-raw.TIF]

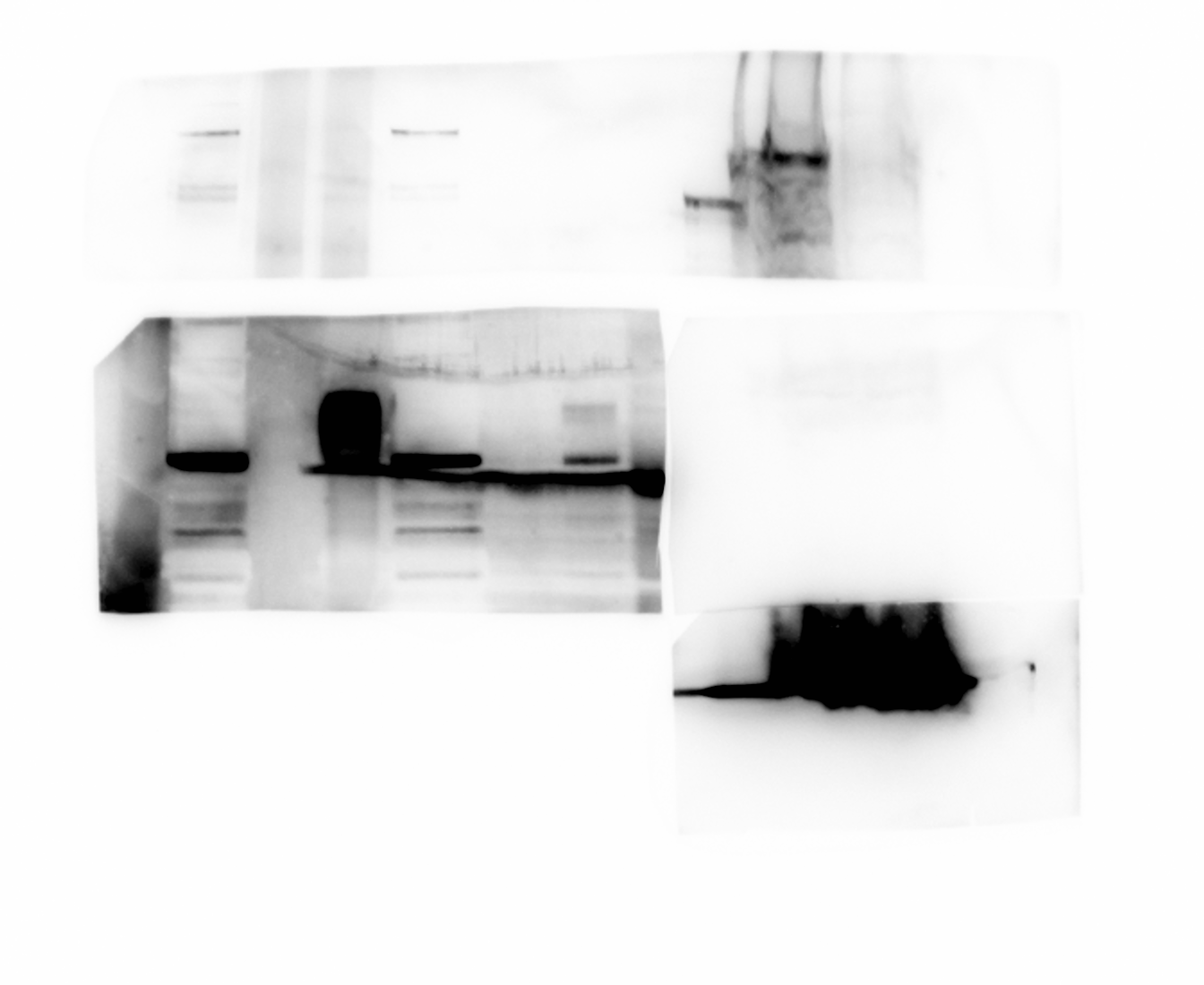

Supplement: Figure 3—figure supplement 1—source data 1. [file elife-82703-fig3-figsupp1-data1.zip › Figure 3-figure supplement 1-Source data 1/Figure 3 source data1/Figure 3-supplement-1E/Figure 3-supplement-1E-clone1-YTHDC1-raw-inverted.tif]

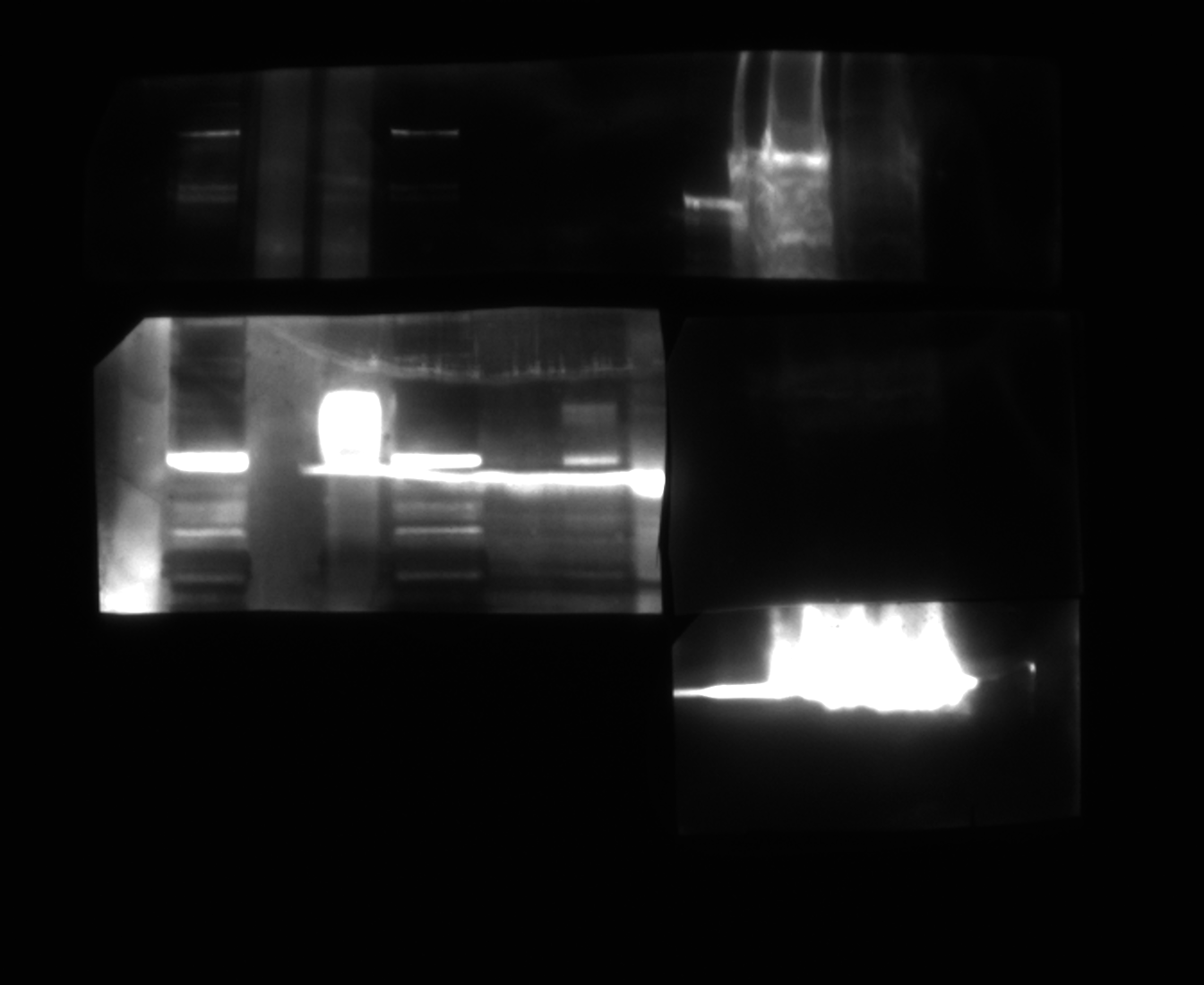

Supplement: Figure 3—figure supplement 1—source data 1. [file elife-82703-fig3-figsupp1-data1.zip › Figure 3-figure supplement 1-Source data 1/Figure 3 source data1/Figure 3-supplement-1E/Figure 3-supplement-1E-clone1-YTHDC1-raw.TIF]

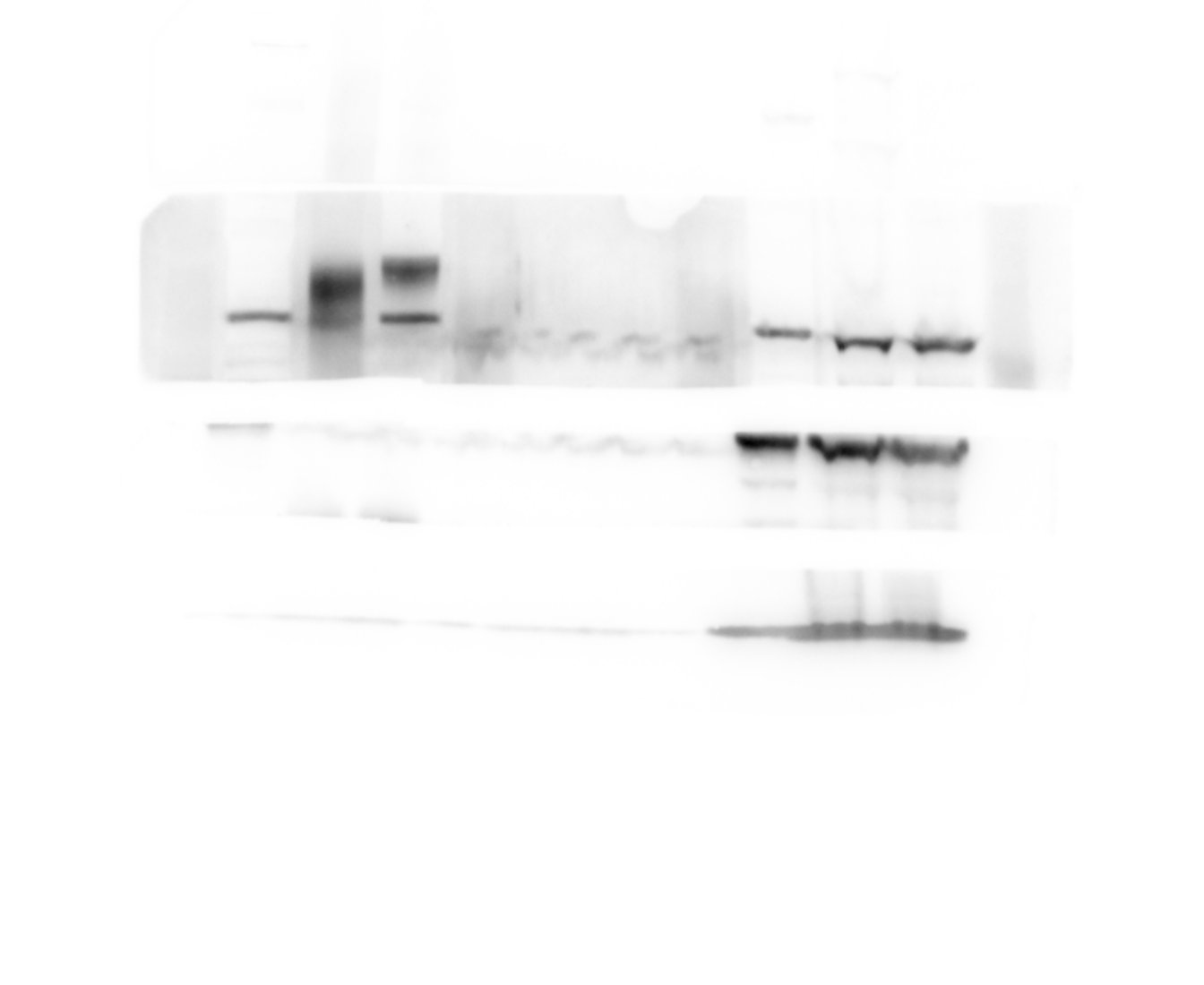

Supplement: Figure 3—figure supplement 1—source data 1. [file elife-82703-fig3-figsupp1-data1.zip › Figure 3-figure supplement 1-Source data 1/Figure 3 source data1/Figure 3-supplement-1E/Figure 3-supplement-1E-clone2-Histone H3and GAPDH-raw-inverted.jpg]

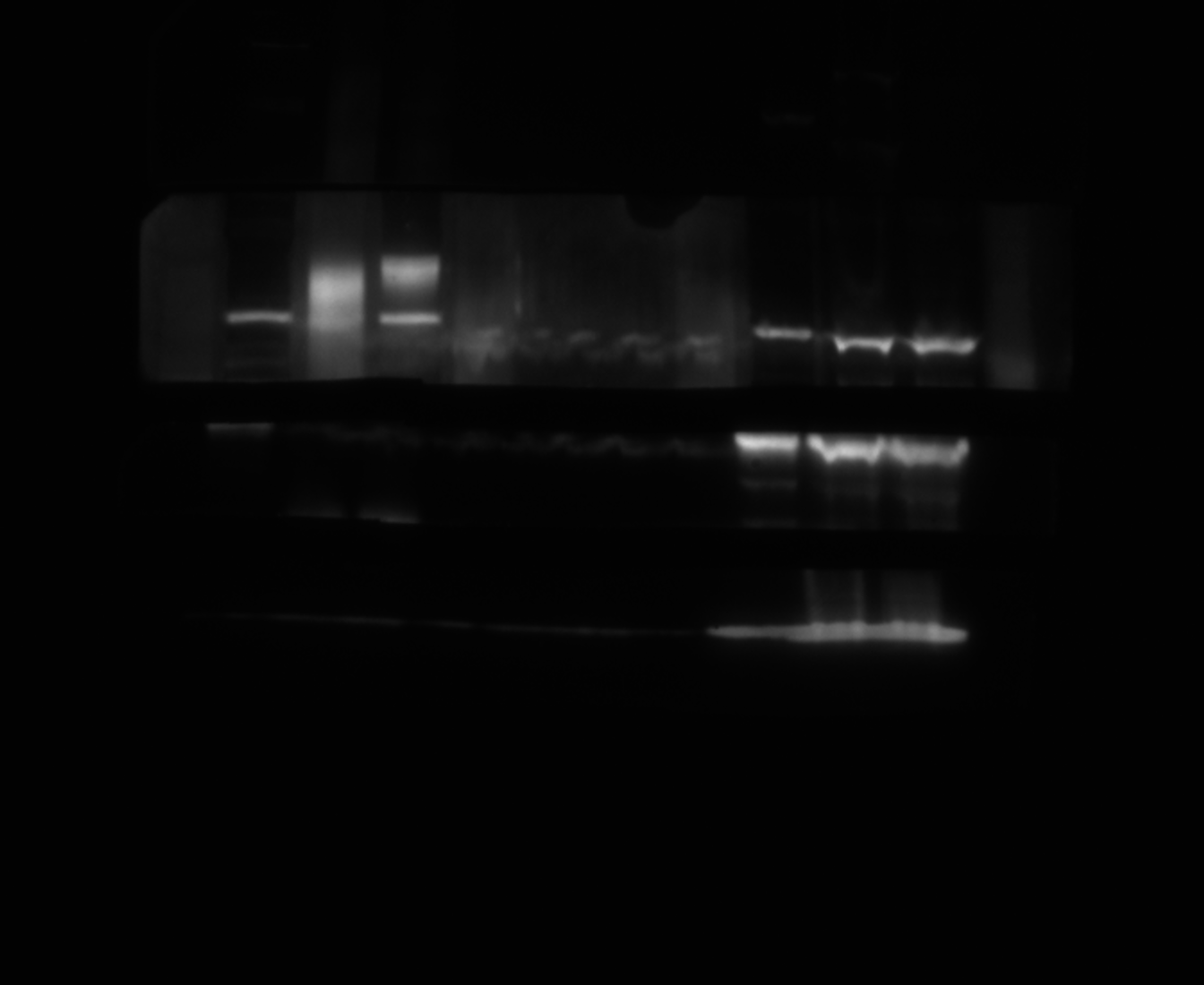

Supplement: Figure 3—figure supplement 1—source data 1. [file elife-82703-fig3-figsupp1-data1.zip › Figure 3-figure supplement 1-Source data 1/Figure 3 source data1/Figure 3-supplement-1E/Figure 3-supplement-1E-clone2-Histone H3and GAPDH-raw.TIF]

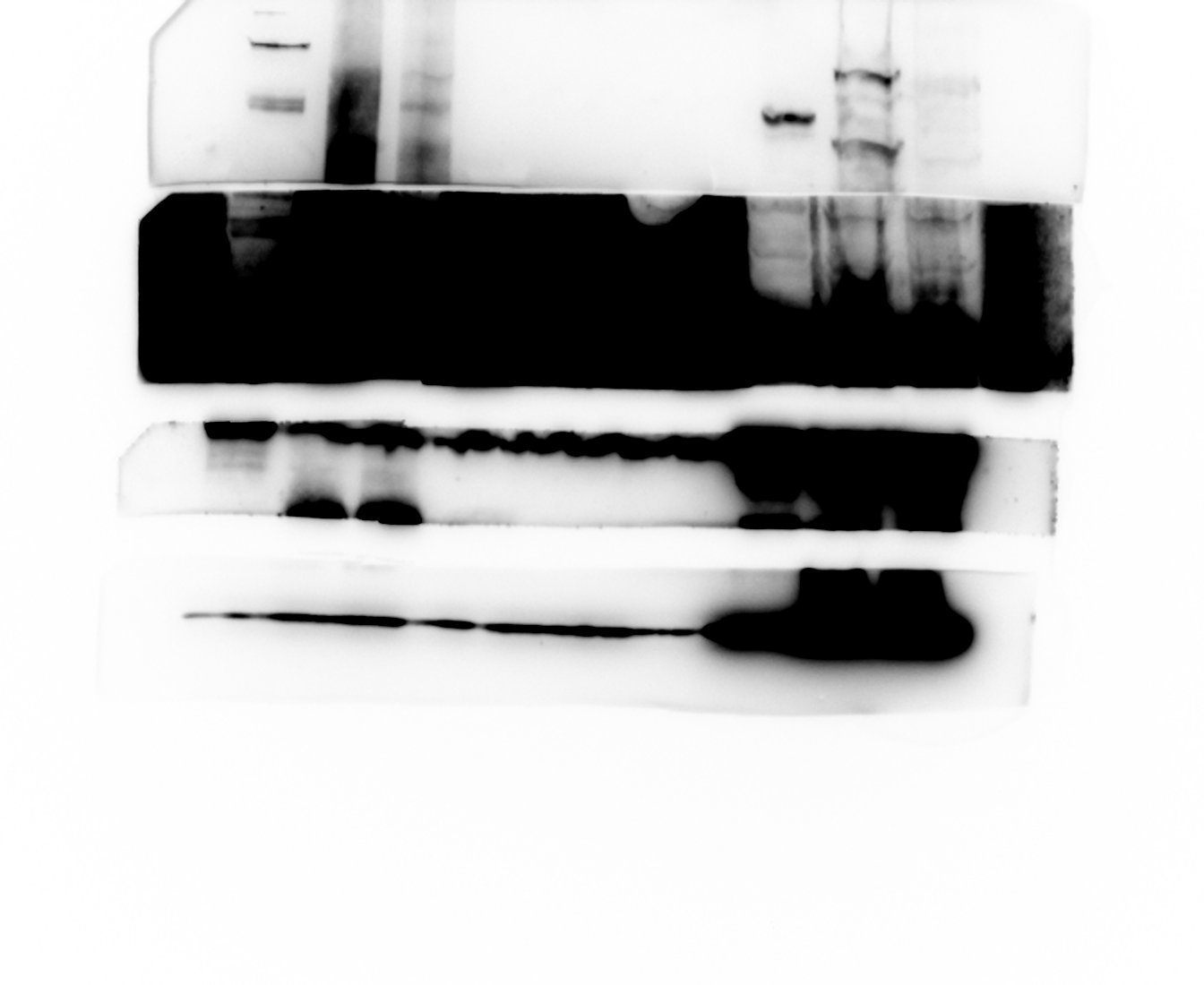

Supplement: Figure 3—figure supplement 1—source data 1. [file elife-82703-fig3-figsupp1-data1.zip › Figure 3-figure supplement 1-Source data 1/Figure 3 source data1/Figure 3-supplement-1E/Figure 3-supplement-1E-clone2-YTHDC1-raw-inverted.jpg]

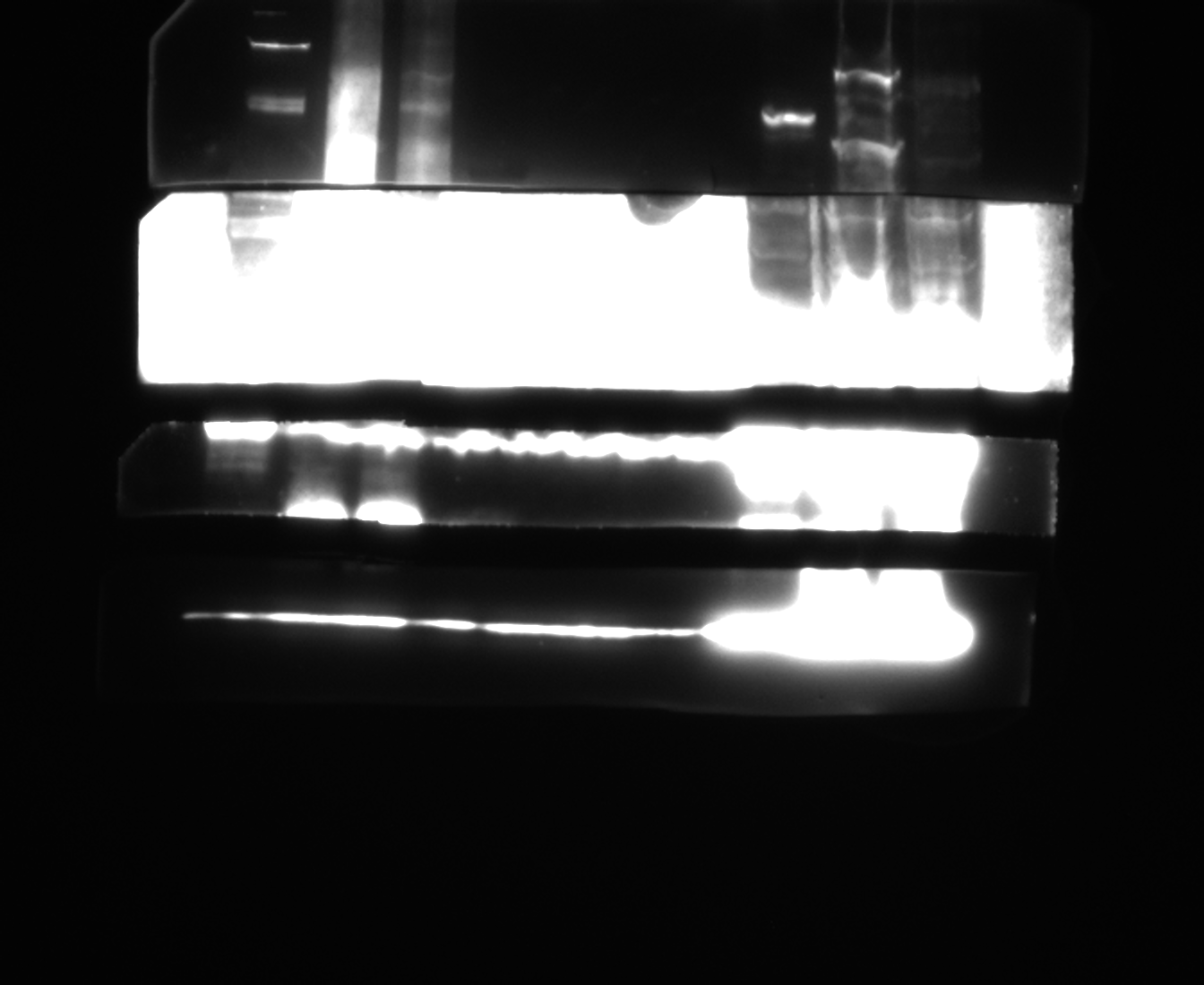

Supplement: Figure 3—figure supplement 1—source data 1. [file elife-82703-fig3-figsupp1-data1.zip › Figure 3-figure supplement 1-Source data 1/Figure 3 source data1/Figure 3-supplement-1E/Figure 3-supplement-1E-clone2-YTHDC1-raw.TIF]

DMSO

IAA

WT

W429A

Mutant

IAA


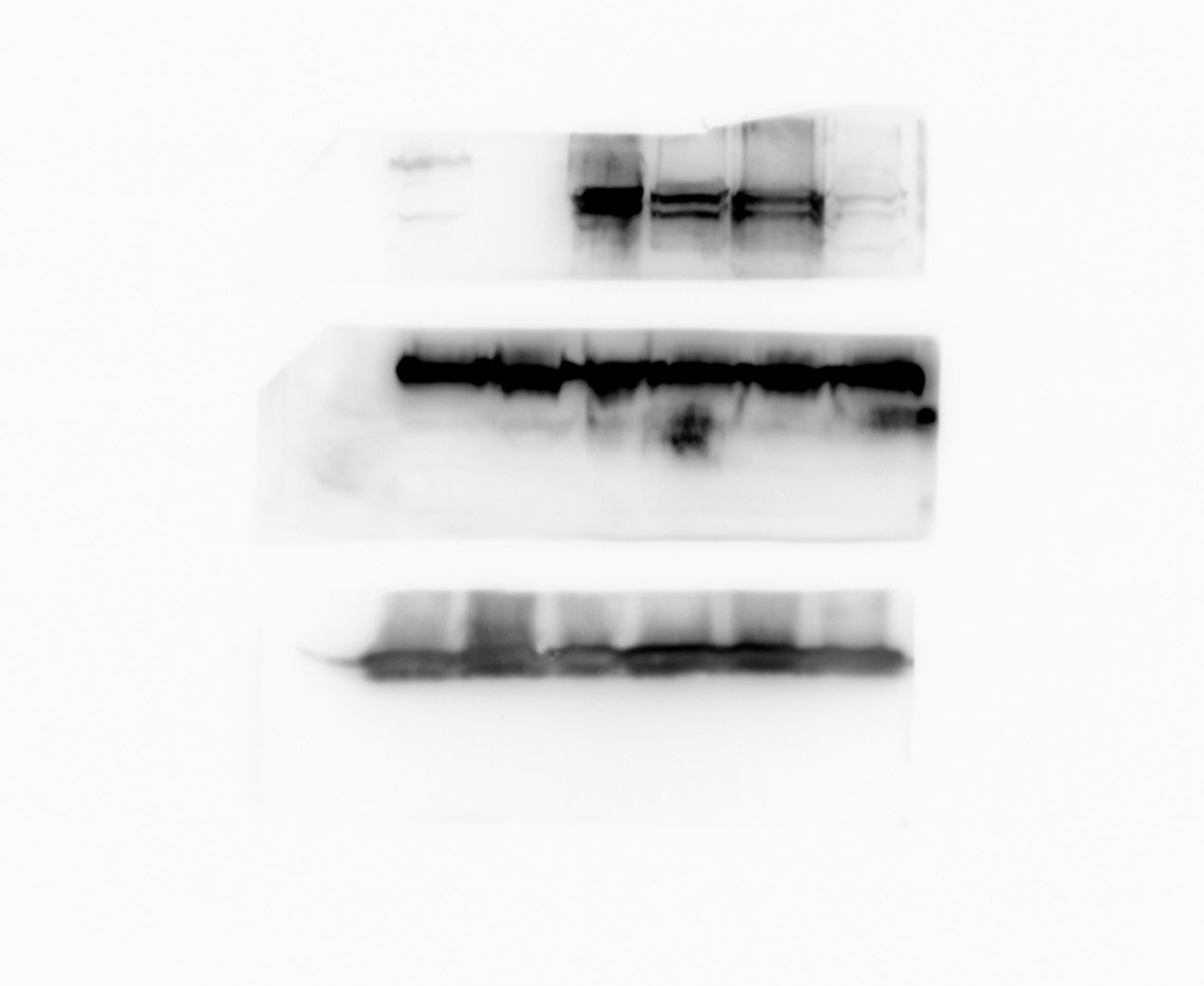


**100kDa**

**YTHDC1**


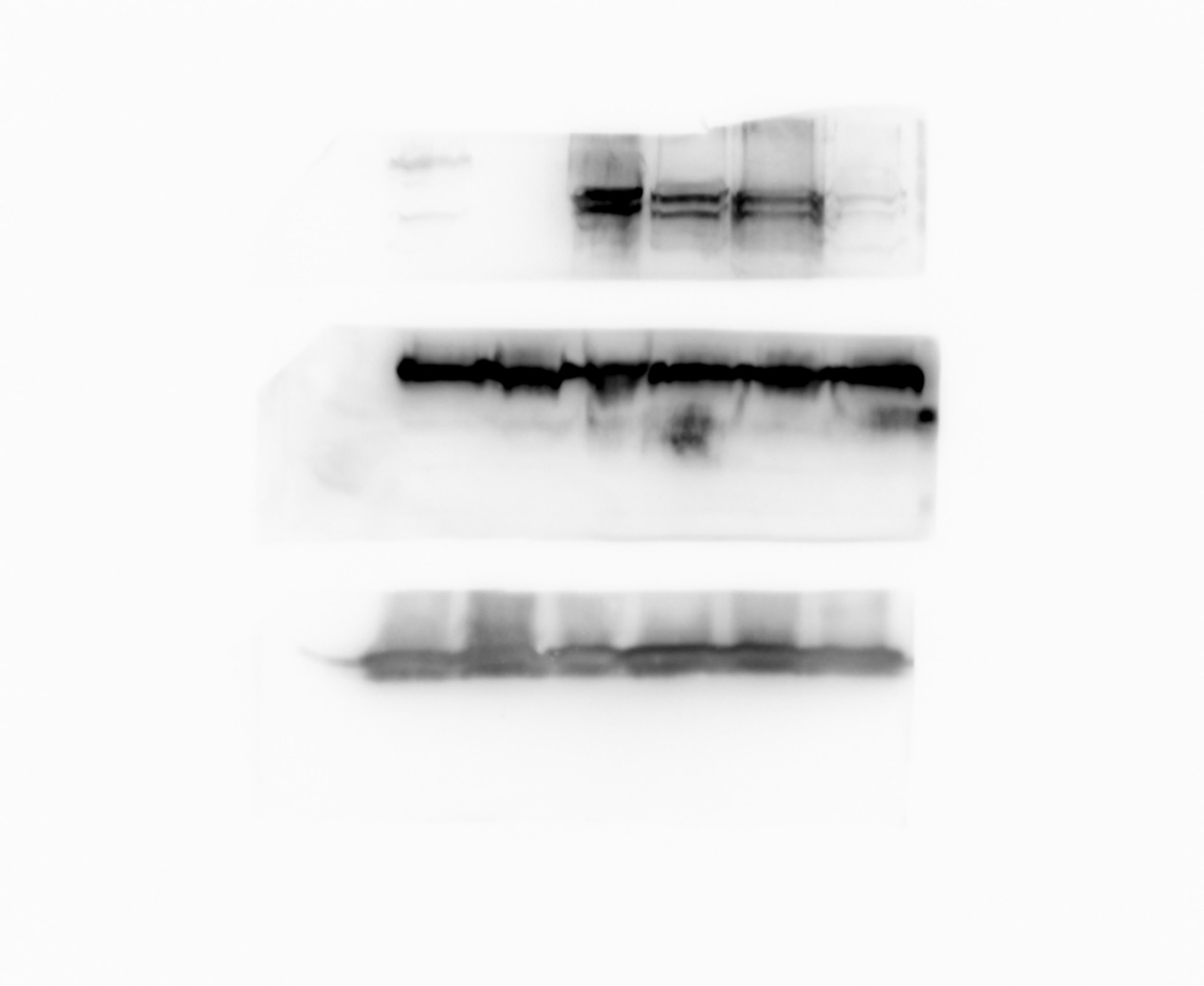


DMSO

IAA

WT

W429A

Mutant

IAA

**GAPDH**

**35kDa**

Supplement: Figure 3—figure supplement 1—source data 1. [file elife-82703-fig3-figsupp1-data1.zip › Figure 3-figure supplement 1-Source data 1/Figure 3 source data1/Figure 3-supplement-1I/Figure 3-supplement-1I-with all relevant bands labelled.docx]

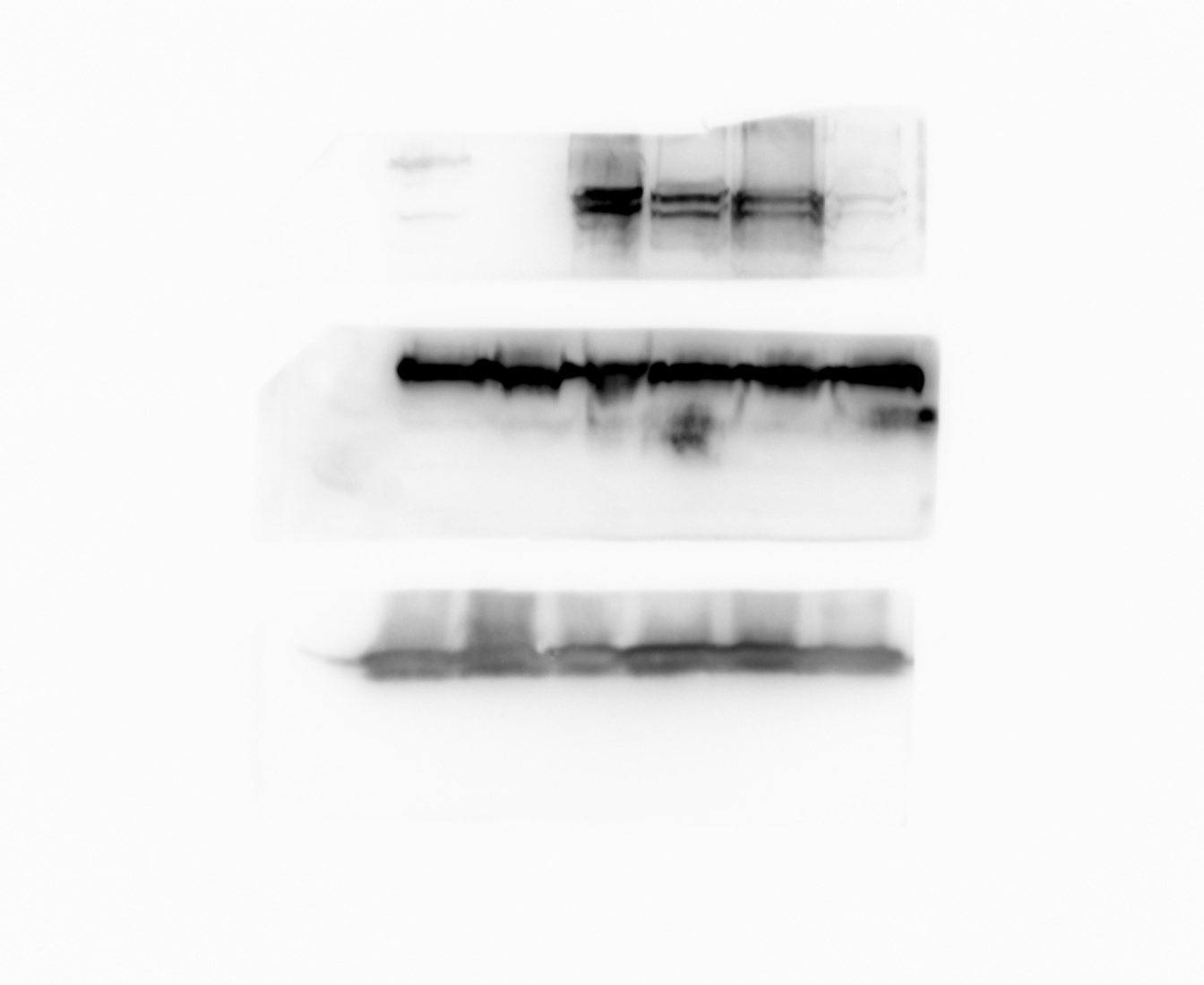

Supplement: Figure 3—figure supplement 1—source data 1. [file elife-82703-fig3-figsupp1-data1.zip › Figure 3-figure supplement 1-Source data 1/Figure 3 source data1/Figure 3-supplement-1I/GAPDH-6s-exporsure-raw-inverted.JPG]

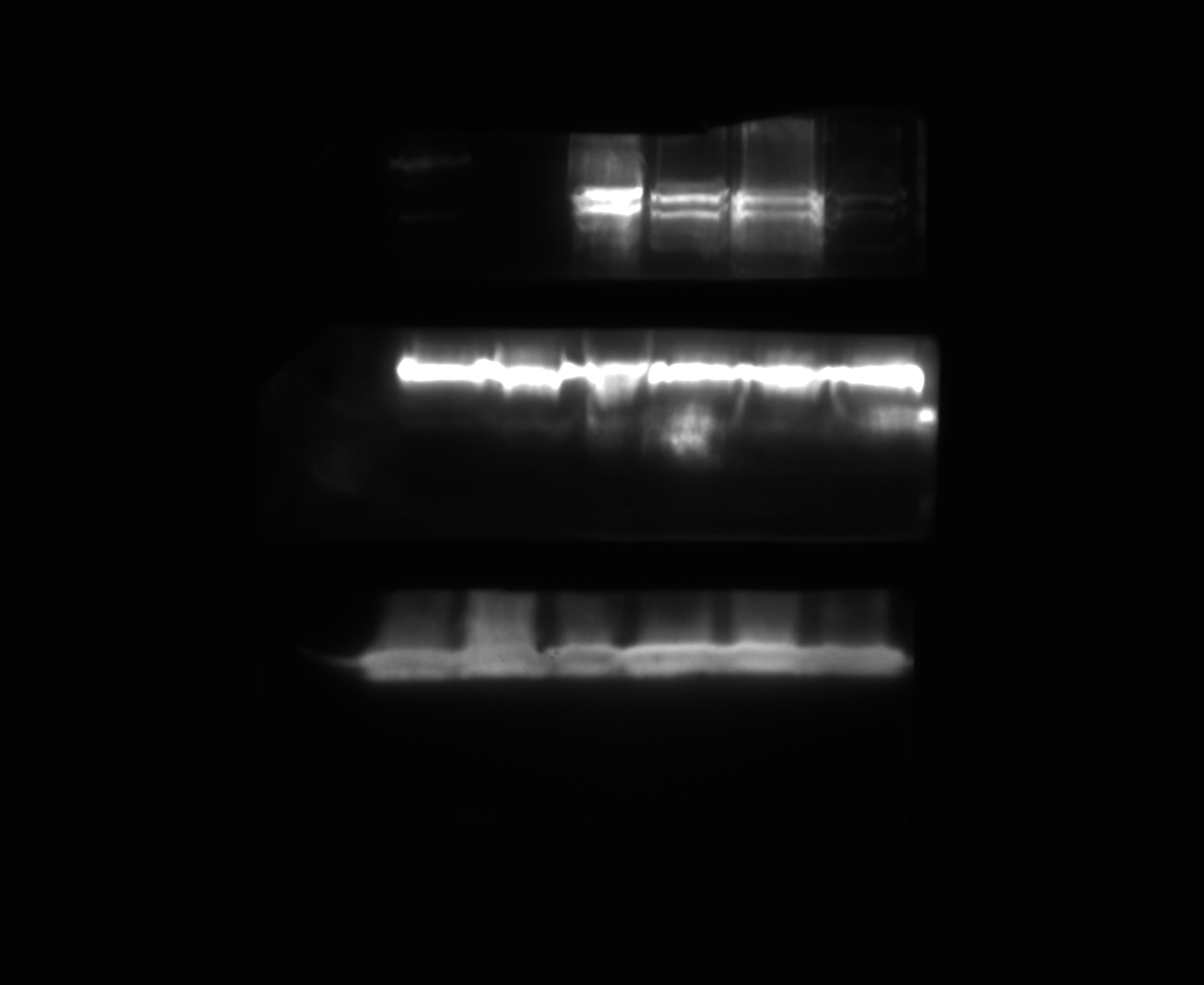

Supplement: Figure 3—figure supplement 1—source data 1. [file elife-82703-fig3-figsupp1-data1.zip › Figure 3-figure supplement 1-Source data 1/Figure 3 source data1/Figure 3-supplement-1I/GAPDH-6s-exporsure-raw.TIF]

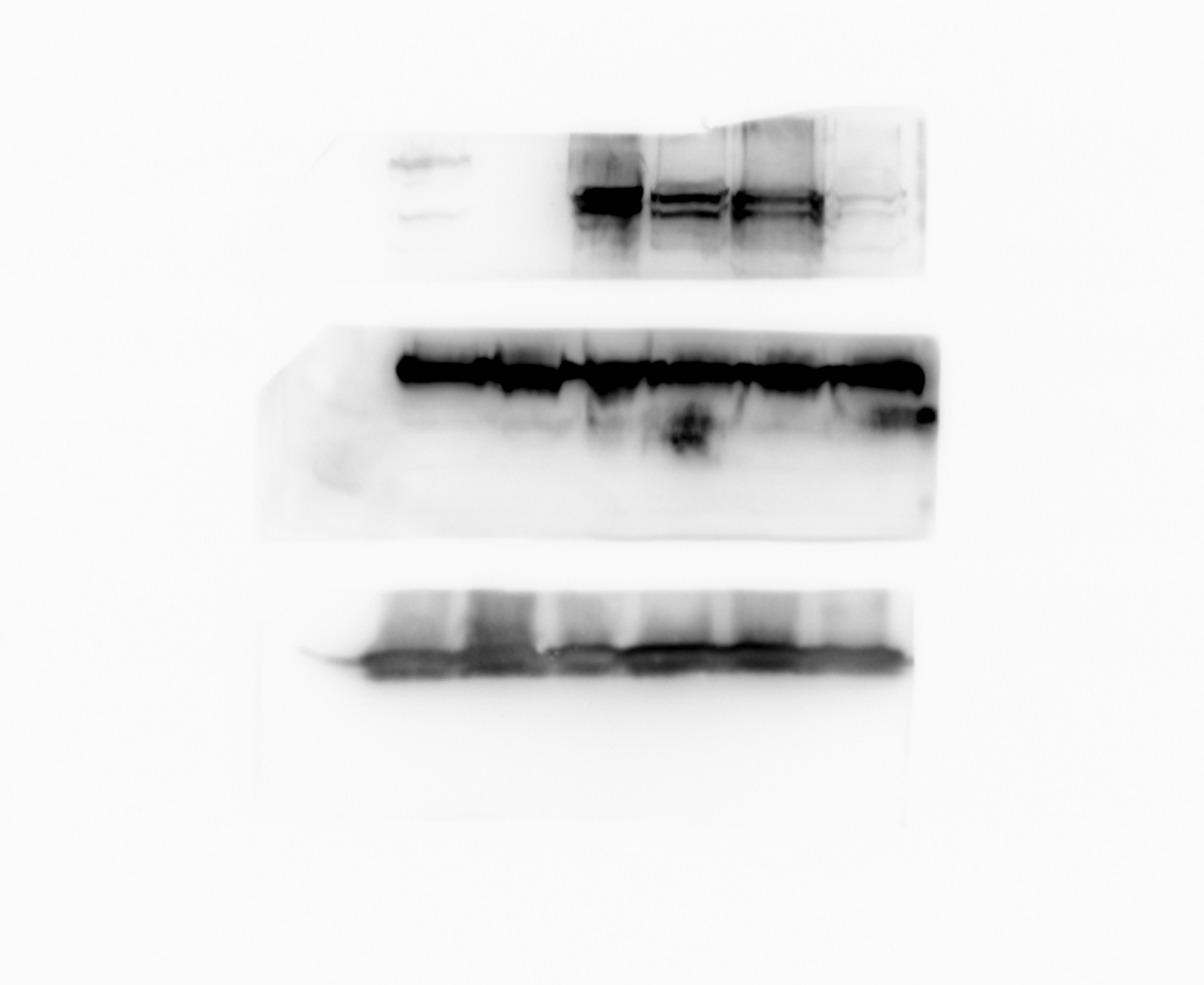

Supplement: Figure 3—figure supplement 1—source data 1. [file elife-82703-fig3-figsupp1-data1.zip › Figure 3-figure supplement 1-Source data 1/Figure 3 source data1/Figure 3-supplement-1I/YTHDC1-8s exposure-raw-inverted.JPG]

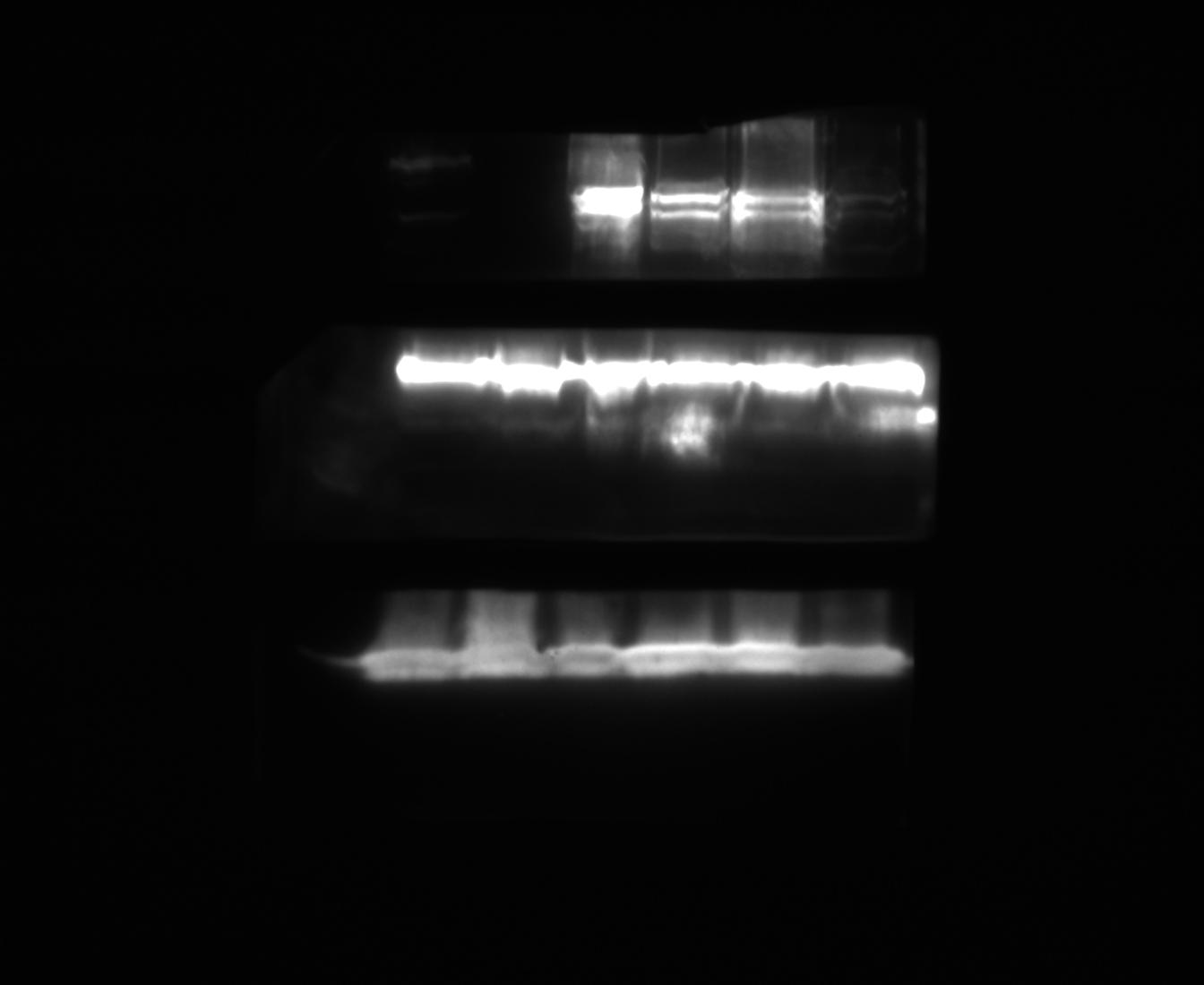

Supplement: Figure 3—figure supplement 1—source data 1. [file elife-82703-fig3-figsupp1-data1.zip › Figure 3-figure supplement 1-Source data 1/Figure 3 source data1/Figure 3-supplement-1I/YTHDC1-8s exposure-raw.TIF]

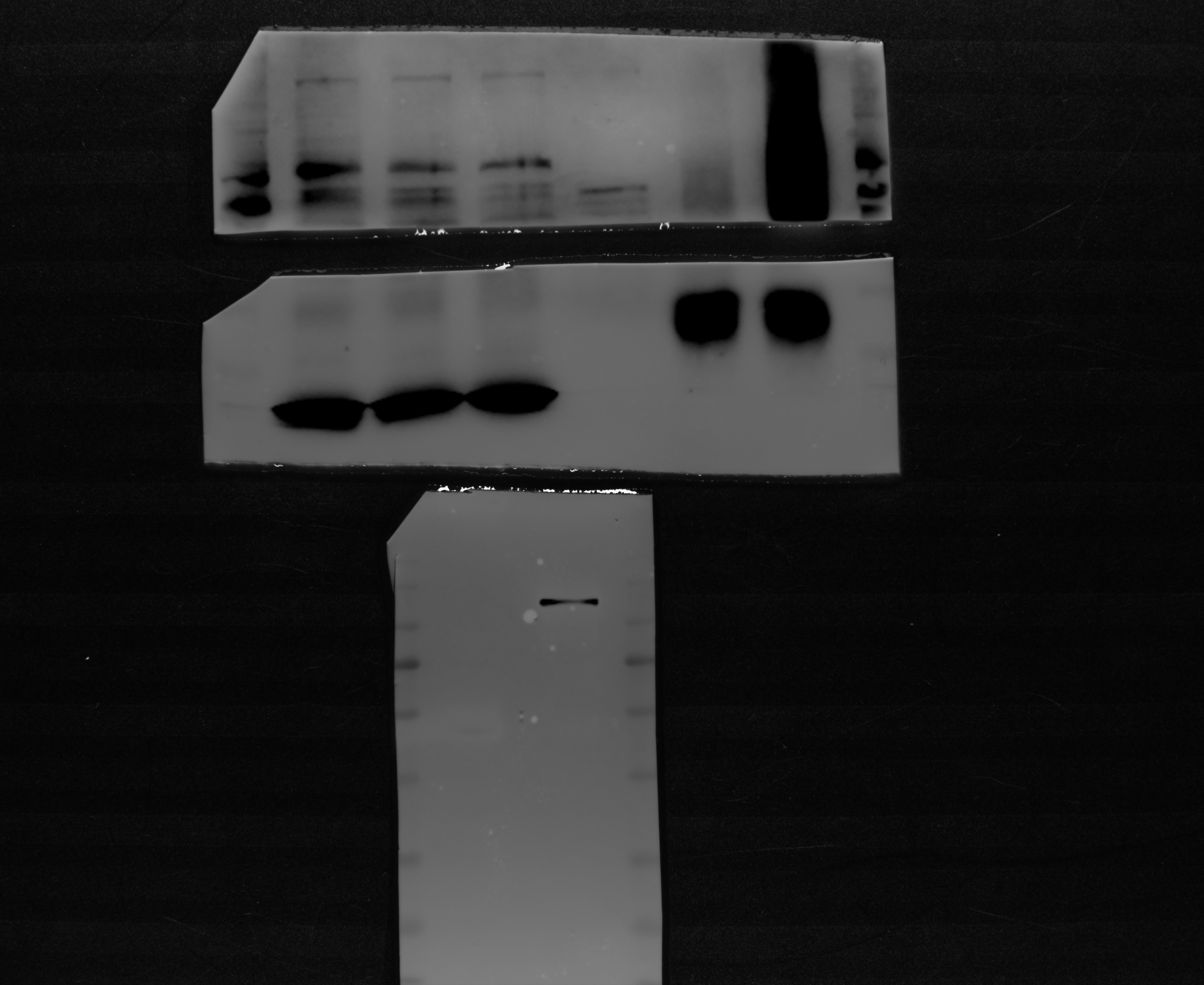

Supplement: Figure 7—source data 1. [file elife-82703-fig7-data1.zip › Figure 7 source data1/Figure 7B-Anti-Flag-raw-multi-Tiff-files with marker-signal-merged.Tif]

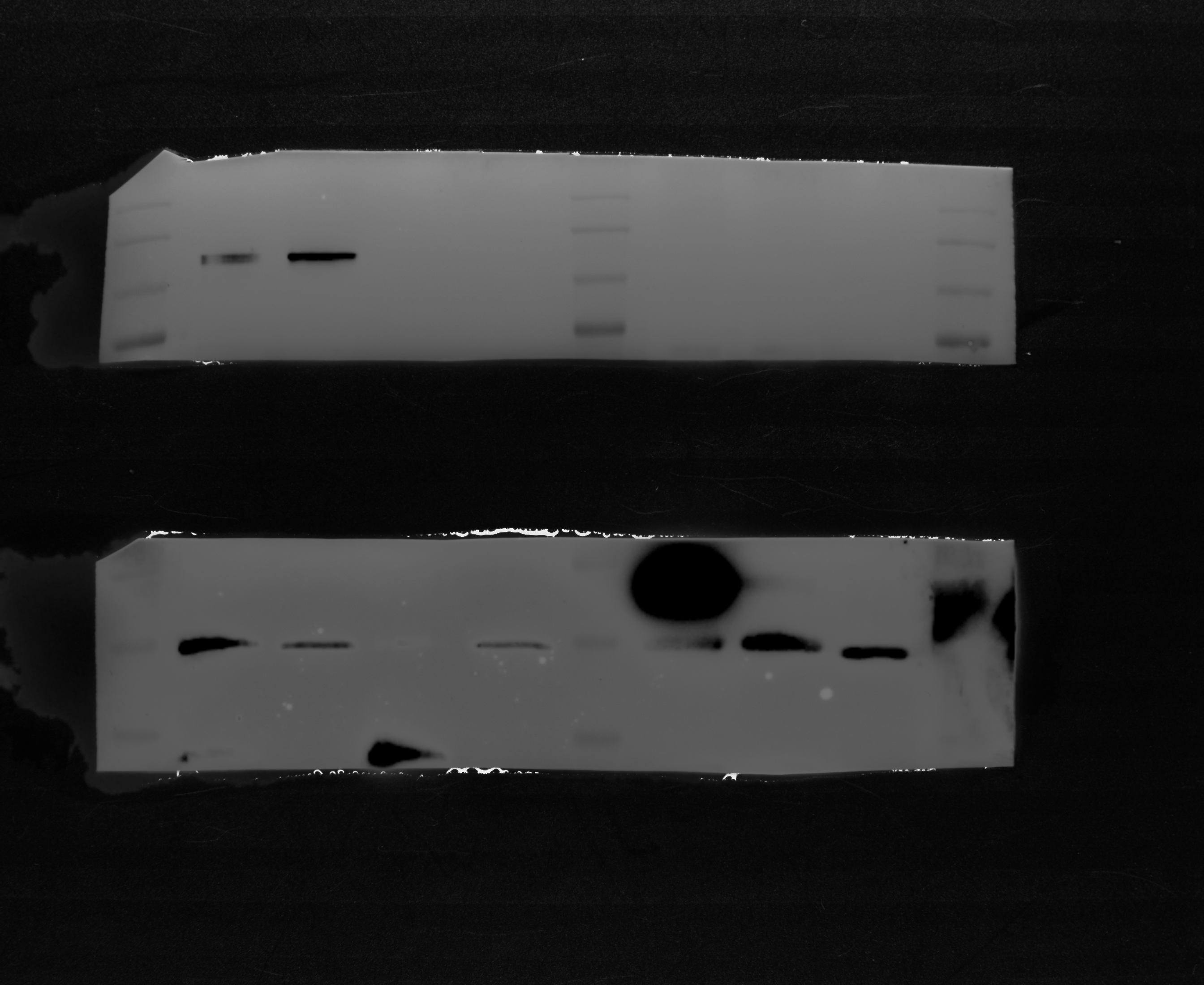

Supplement: Figure 7—source data 1. [file elife-82703-fig7-data1.zip › Figure 7 source data1/Figure 7H-Anti hnRNPG and YTHDC1-raw-multi-Tiff-files with marker-signal-merged.Tif]

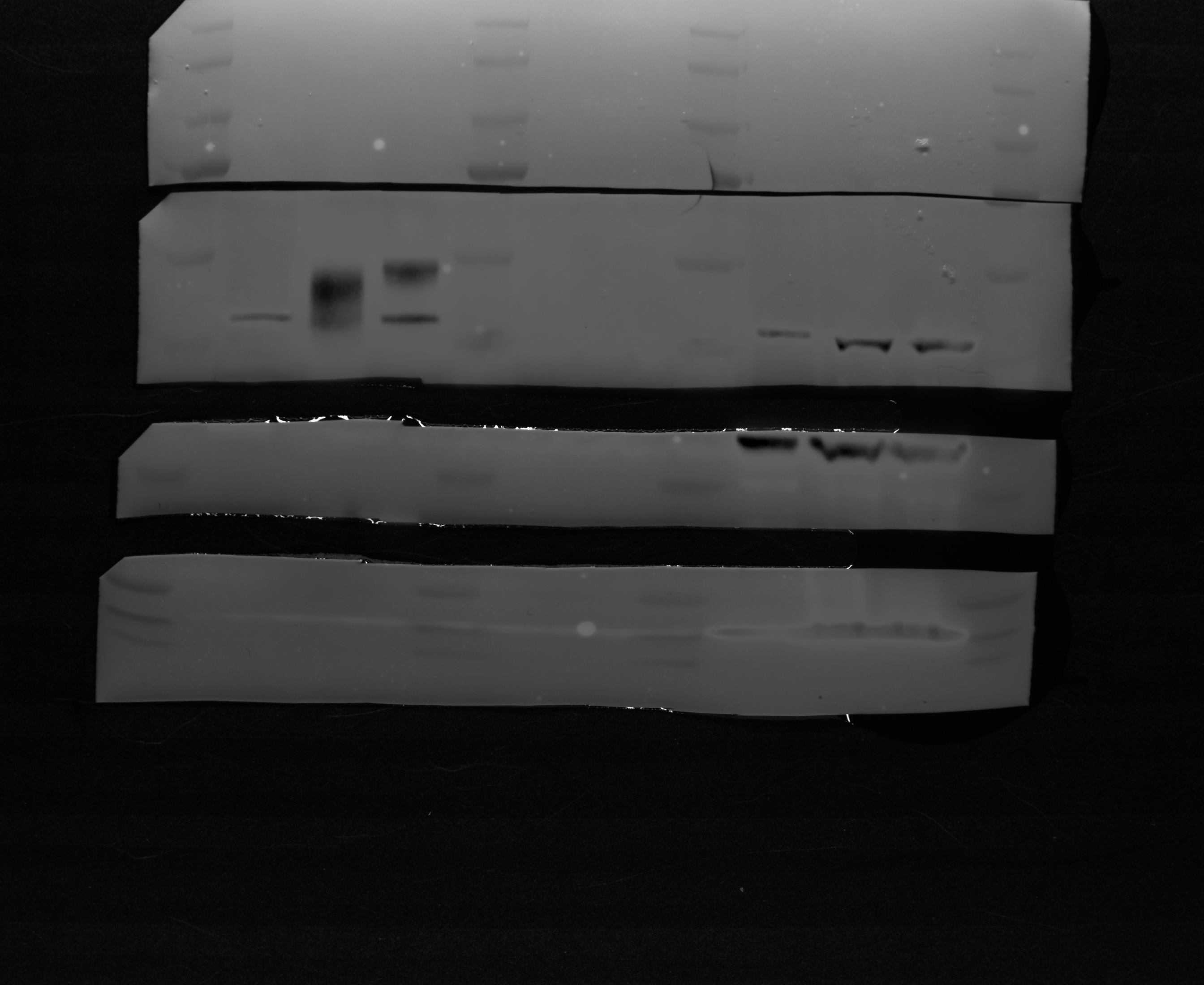

Supplement: Figure 7—source data 2. [file elife-82703-fig7-data2.zip › Figure 7 source data2/Figure 7I-anti hnRNPG-raw-mluti-Tiff-file-signal-marker-merged.Tif]

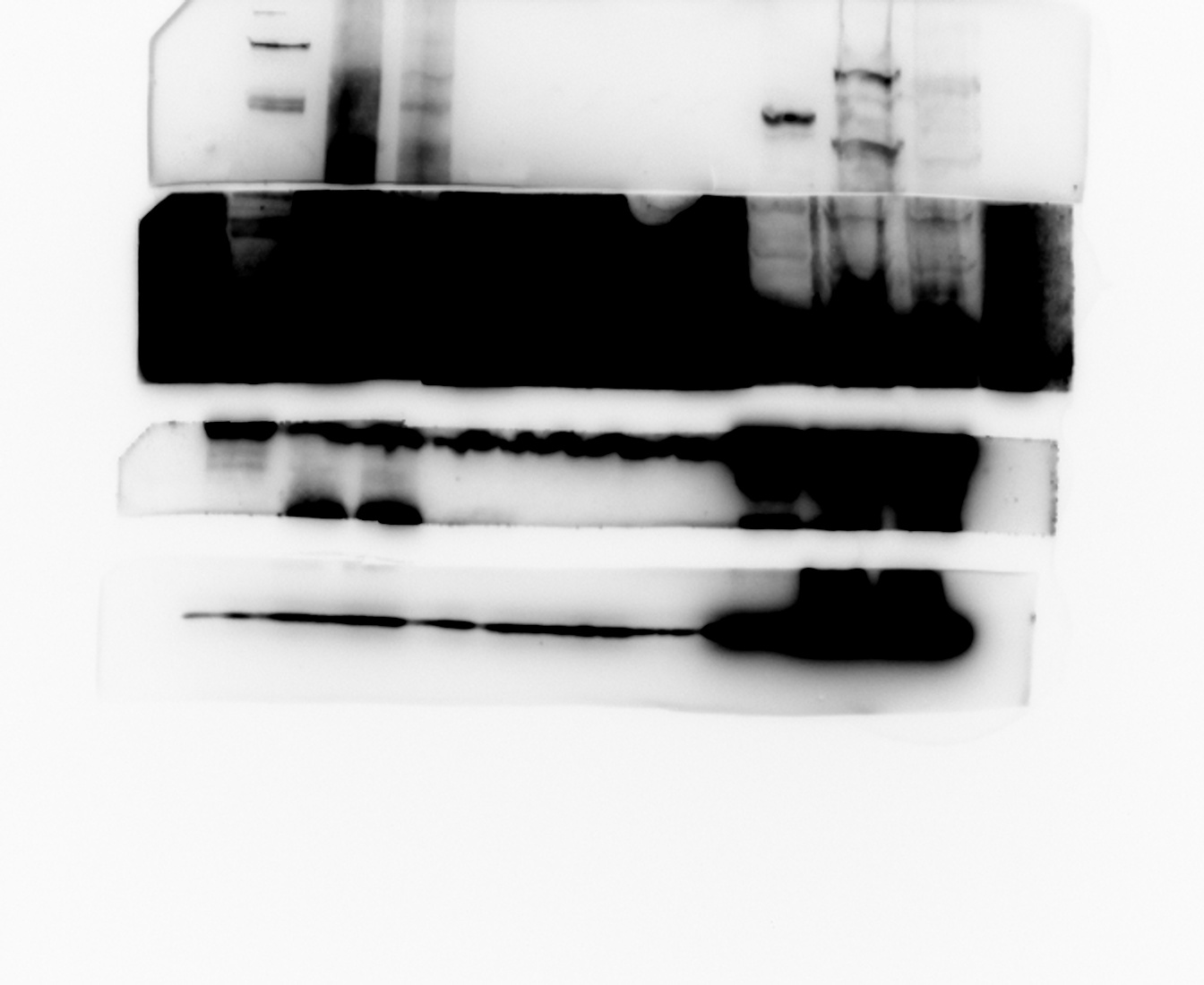

Supplement: Figure 7—source data 2. [file elife-82703-fig7-data2.zip › Figure 7 source data2/Figure 7I-anti YTHDC1-raw-inverted.JPG]

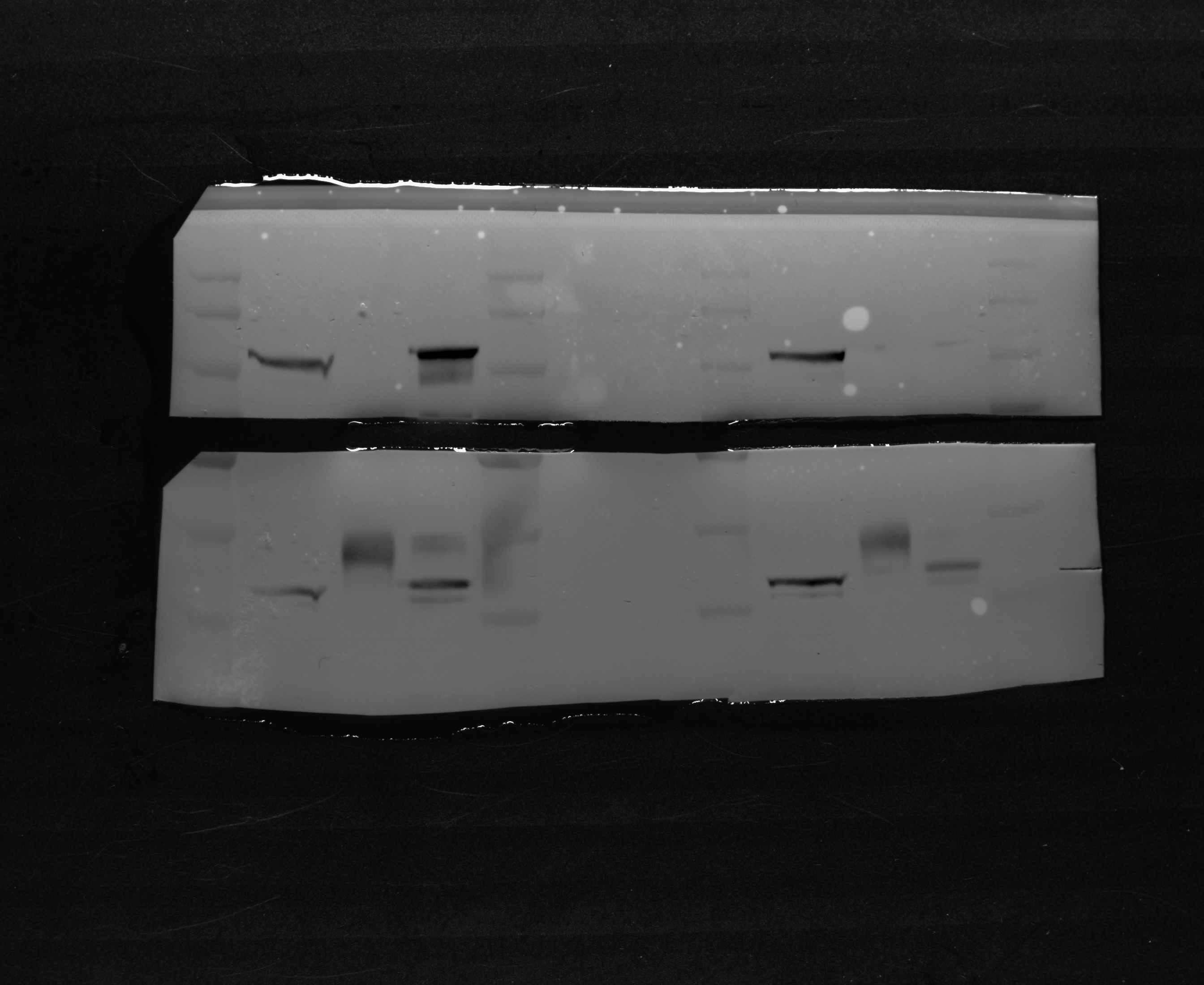

Supplement: Figure 7—source data 2. [file elife-82703-fig7-data2.zip › Figure 7 source data2/Figure 7J-anti YTHDC1 and hnRNPG-raw-multi-Tiff files-signal-marker-merged.Tif]

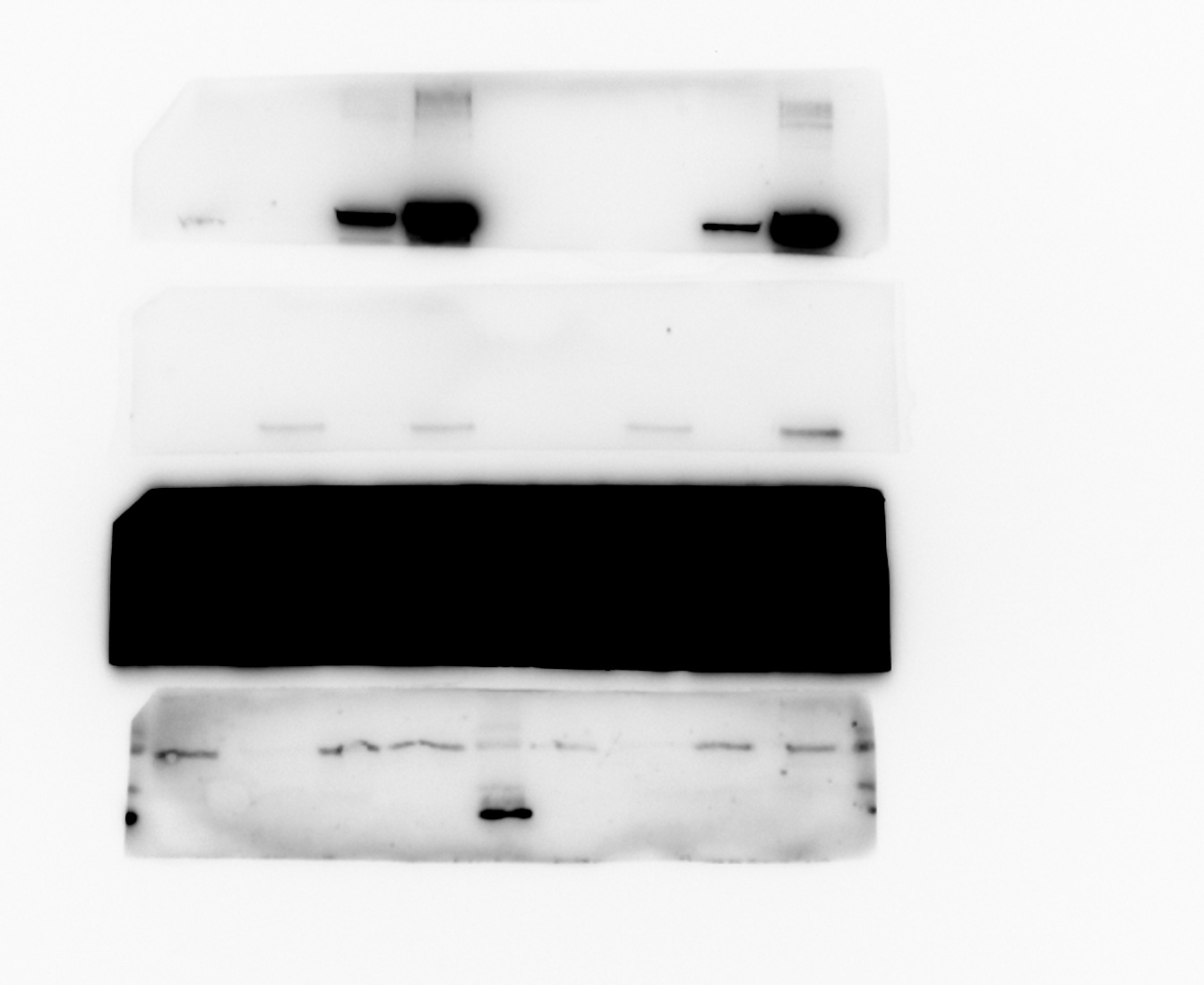

Supplement: Figure 7—source data 3. [file elife-82703-fig7-data3.zip › figure 7L/Figure 7L-thoc7-raw-inverted.JPG]

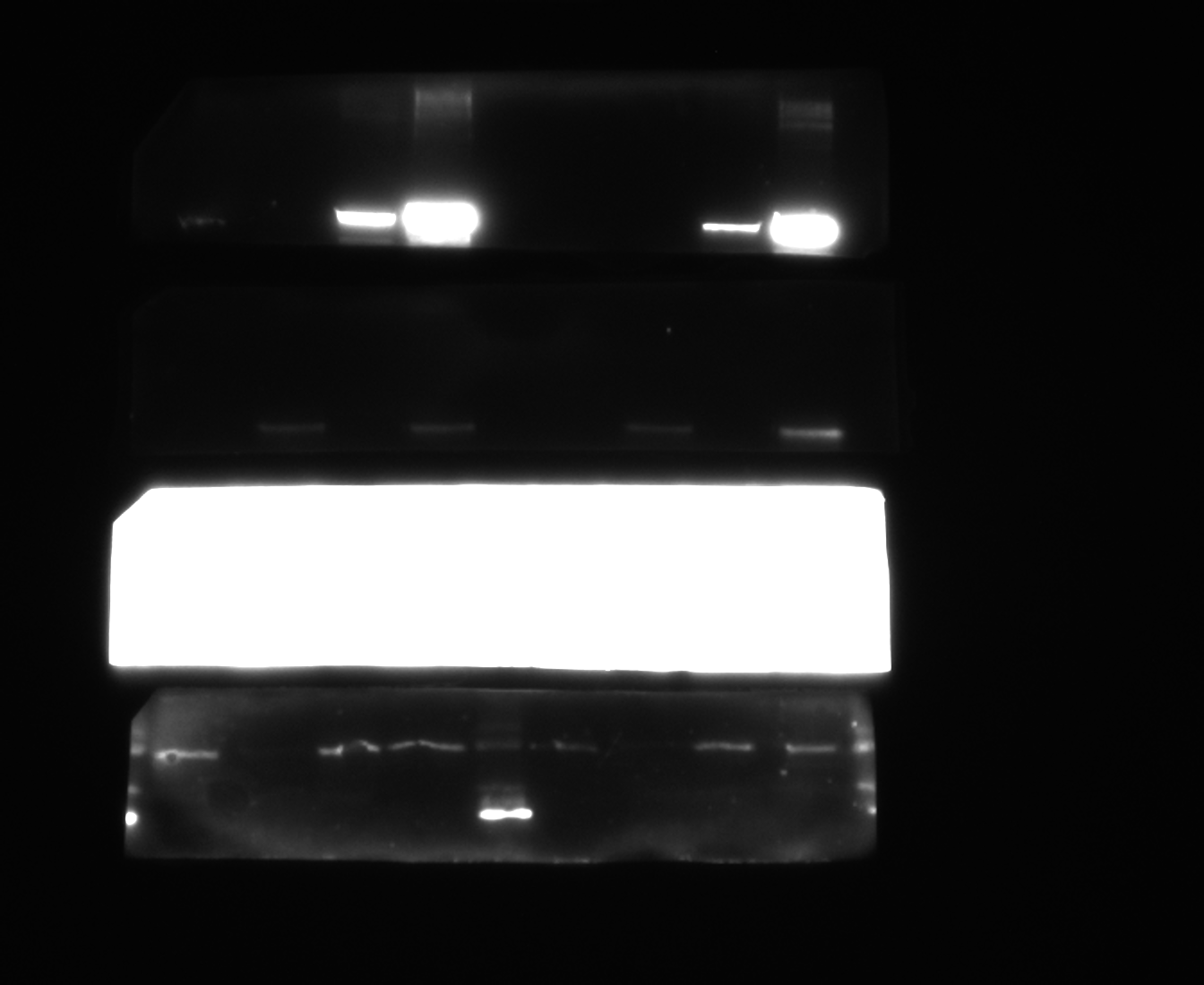

Supplement: Figure 7—source data 3. [file elife-82703-fig7-data3.zip › figure 7L/Figure 7L-thoc7-raw.TIF]

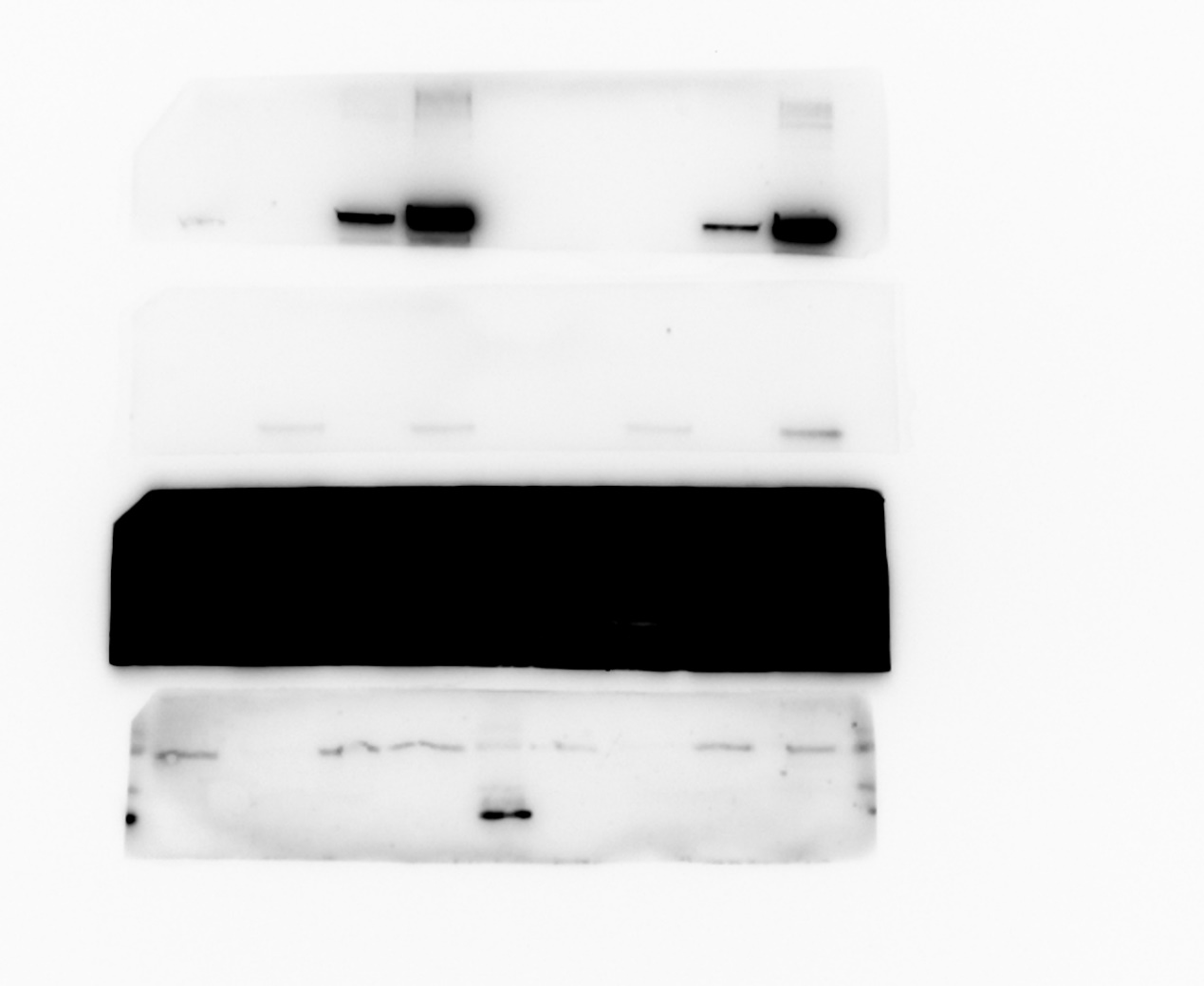

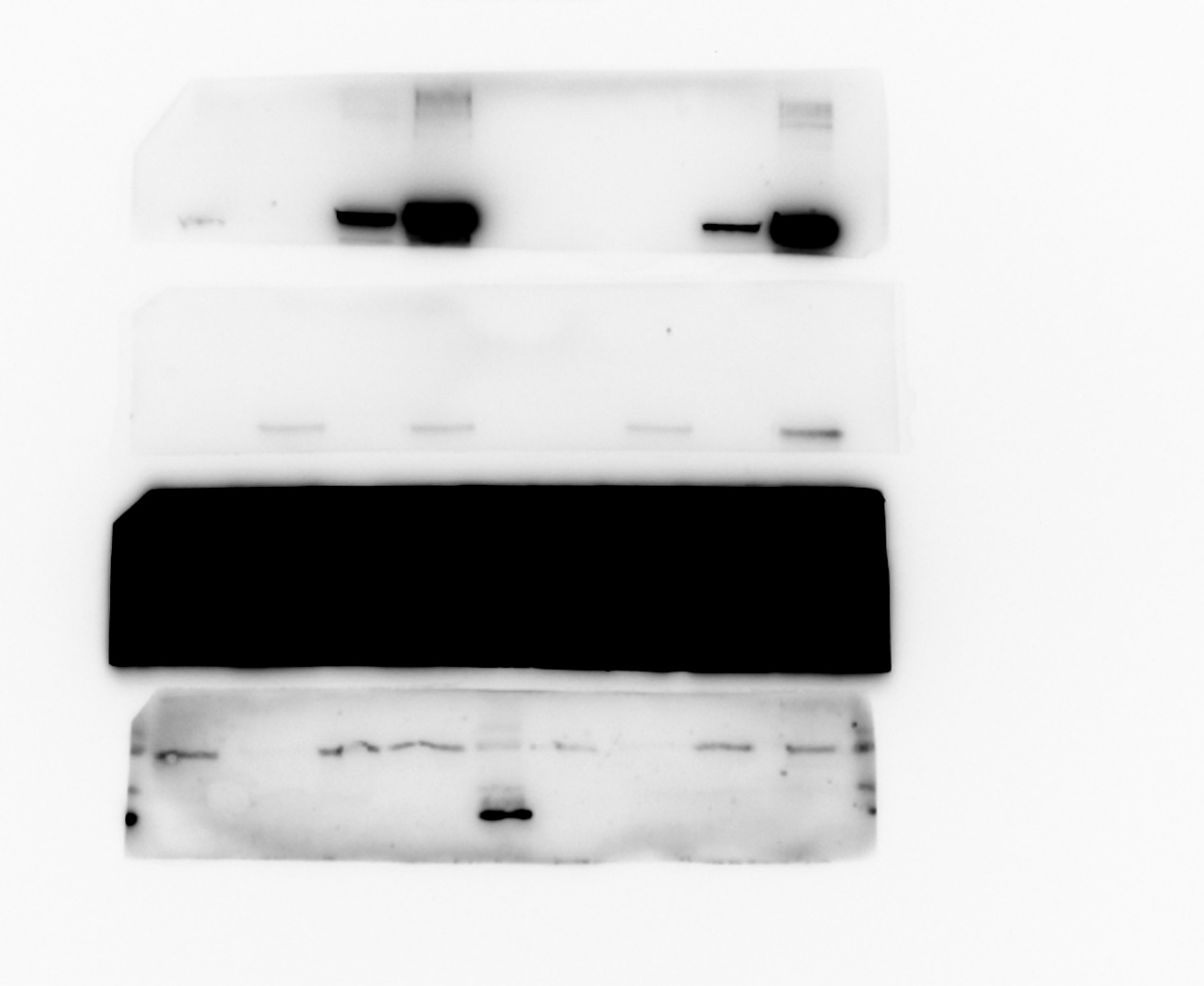


**25kDa**

**100kDa**

Input

IP:Flag

Input

IP:Flag

Input

IP:Flag

IP:Flag

Input

**THOC7**

**YTHDC1**

Supplement: Figure 7—source data 3. [file elife-82703-fig7-data3.zip › figure 7L/Figure 7L-with all relevant bands labelled.docx]

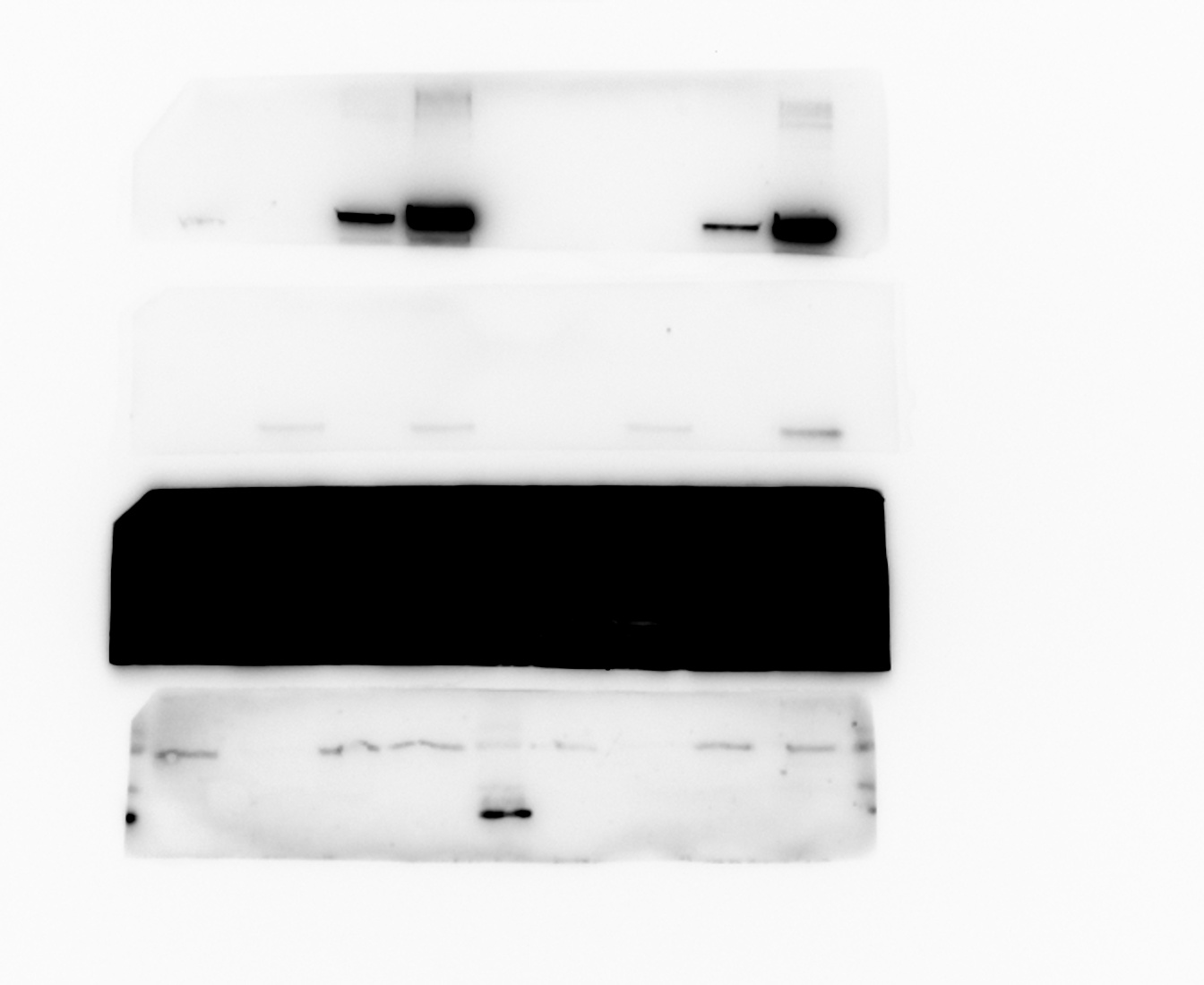

Supplement: Figure 7—source data 3. [file elife-82703-fig7-data3.zip › figure 7L/Figure 7L-ythdc1-raw-inverted.JPG]

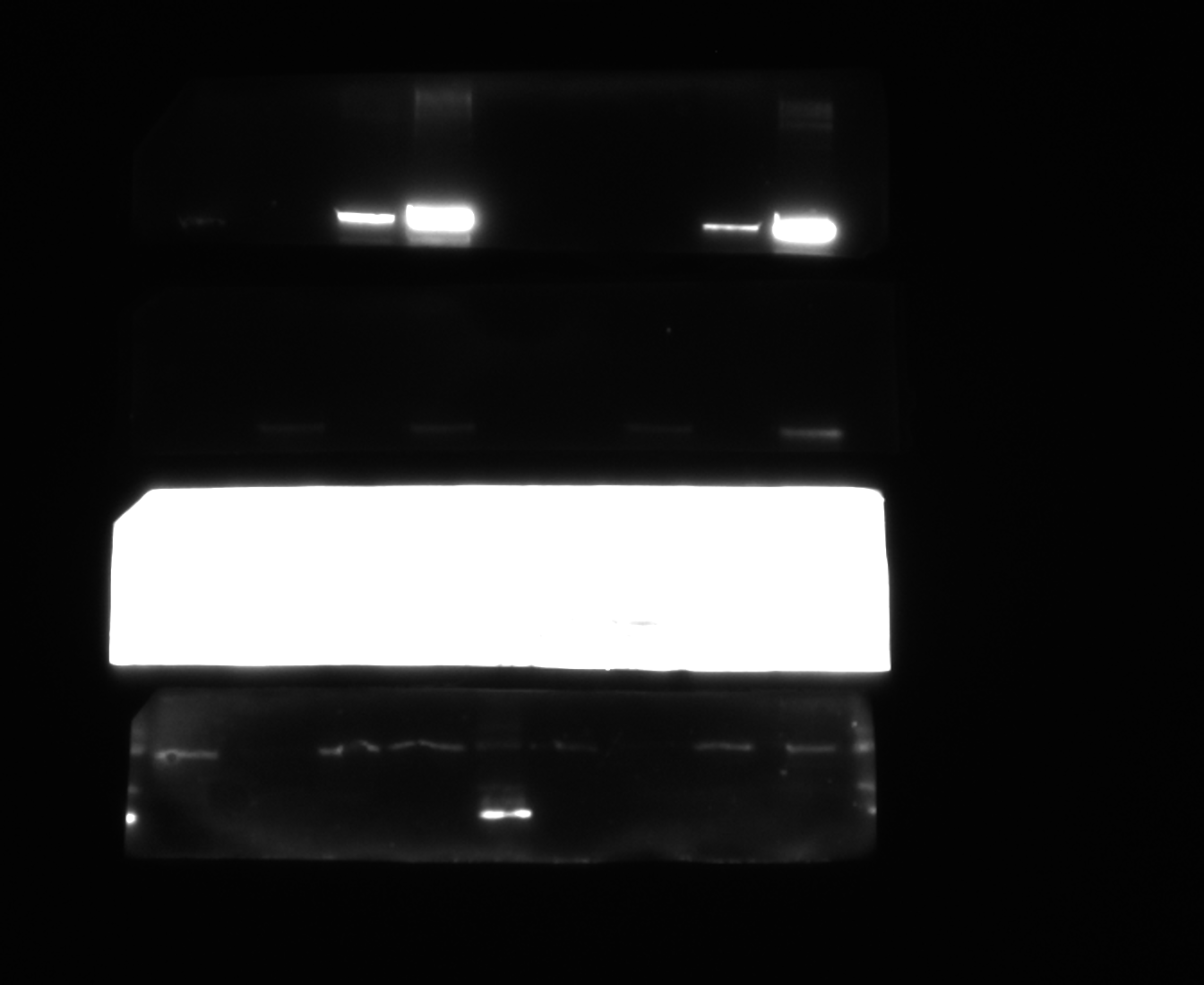

Supplement: Figure 7—source data 3. [file elife-82703-fig7-data3.zip › figure 7L/Figure 7L-ythdc1-raw.TIF]
