# Supplementary material for: Nuclear m6A reader YTHDC1 promotes muscle stem cell activation/proliferation by regulating mRNA splicing and nuclear export
Source: eLife. 2023 Mar 9;12:e82703. doi: 10.7554/eLife.82703 (PMC10089659; doi:10.7554/eLife.82703)
Supplement: Figure 3—figure supplement 1—source data 1. [file elife-82703-fig3-figsupp1-data1.zip › Figure 3-figure supplement 1-Source data 1/Figure 3 source data1/Figure 3-supplement-1C/Figure 3-supplement-1C-with all relevant bands labelled.docx]

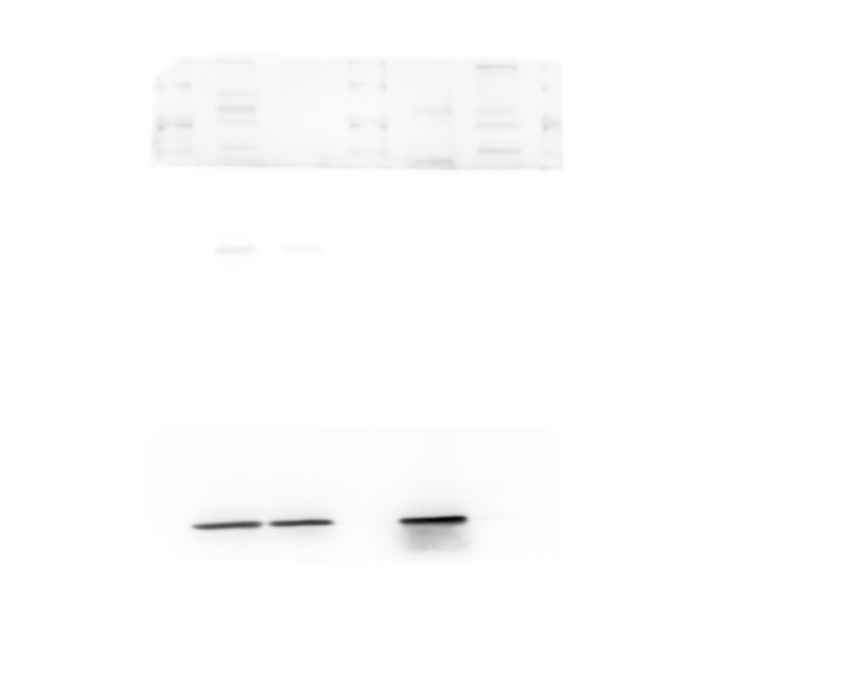
Figure3-supplement-1C-Histone H3

**15kDa**

**Histone H3**

**Ctrl iKO**


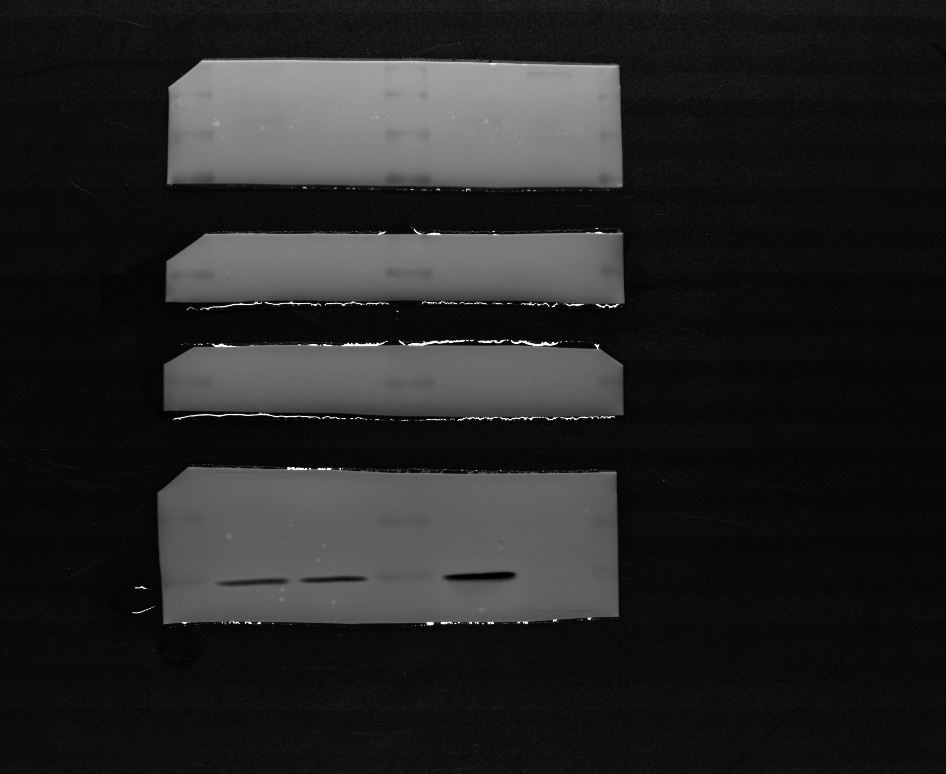


**15kDa**

**Ctrl iKO**

**Histone H3**


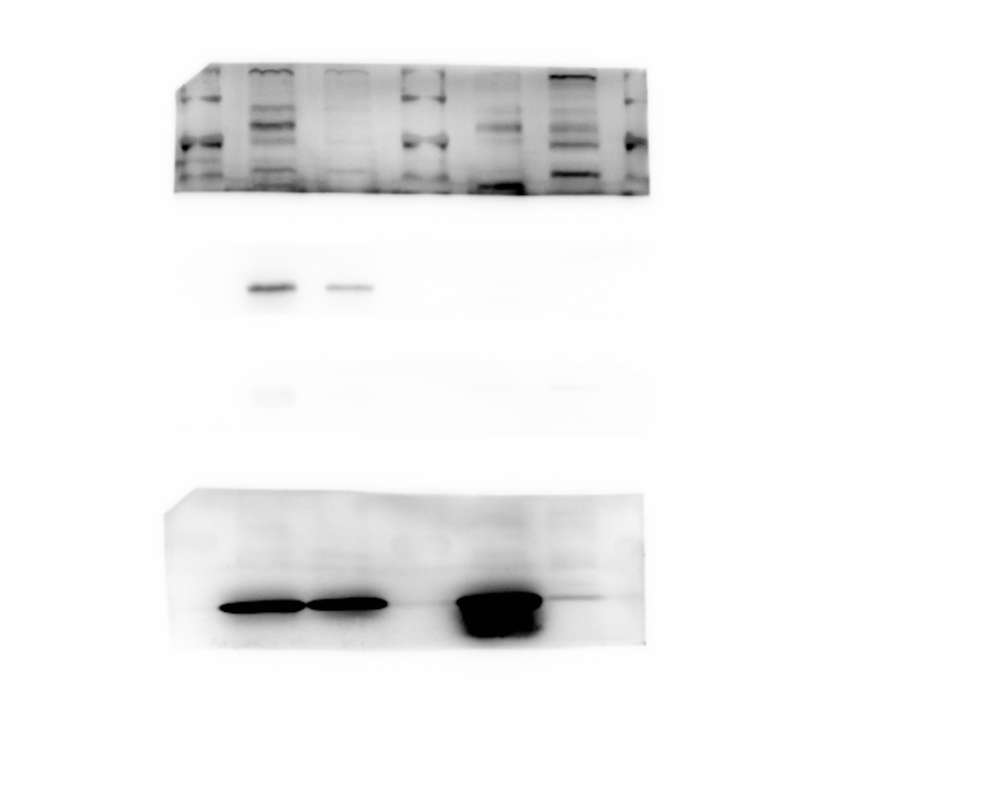
Figure3-supplement-1C-YTHDC1andPax7

**60kDa**

**Pax7**

**100kDa**

**Ctrl iKO**

**YTHDC**

Figure3-supplement-1C-Myod
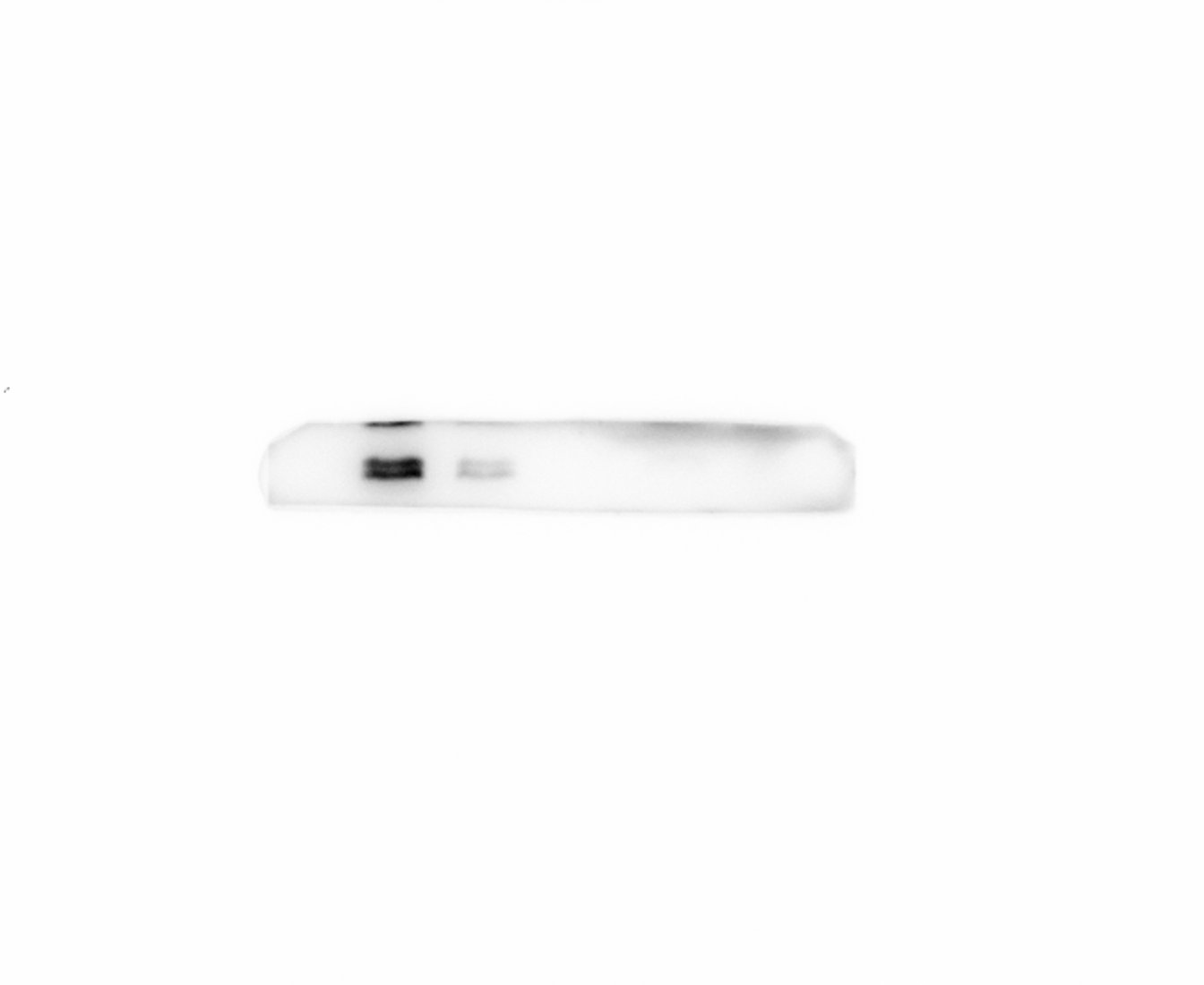


**Myod**

**Ctrl iKO**

**45kDa**
