# Supplementary material for: Nuclear m6A reader YTHDC1 promotes muscle stem cell activation/proliferation by regulating mRNA splicing and nuclear export
Source: eLife. 2023 Mar 9;12:e82703. doi: 10.7554/eLife.82703 (PMC10089659; doi:10.7554/eLife.82703)
Supplement: Figure 3—figure supplement 1—source data 1. [file elife-82703-fig3-figsupp1-data1.zip › Figure 3-figure supplement 1-Source data 1/Figure 3 source data1/Figure 3-supplement-1E/Figure 3-supplement-1E-with all relevant bands labelled.docx]

Figure3-supplement-1E-Clone1-YTHDC1
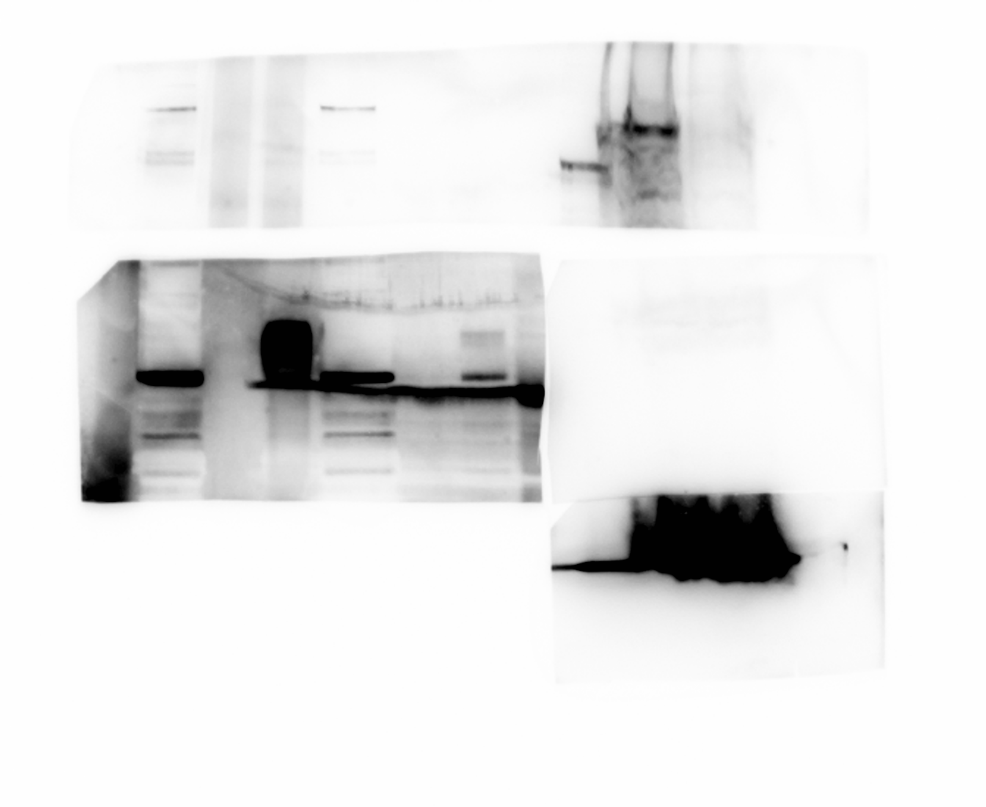


**YTHDC1**

**100kDa**

**Ctrl DMSO IAA**



Figure3-supplement-1E-Clone1-Histone H3

**Myod**

**Ctrl DMSO IAA**

**Histone H3**

**15kDa**

Figure3-supplement-1E-Clone2-Histone H3 and GAPDH


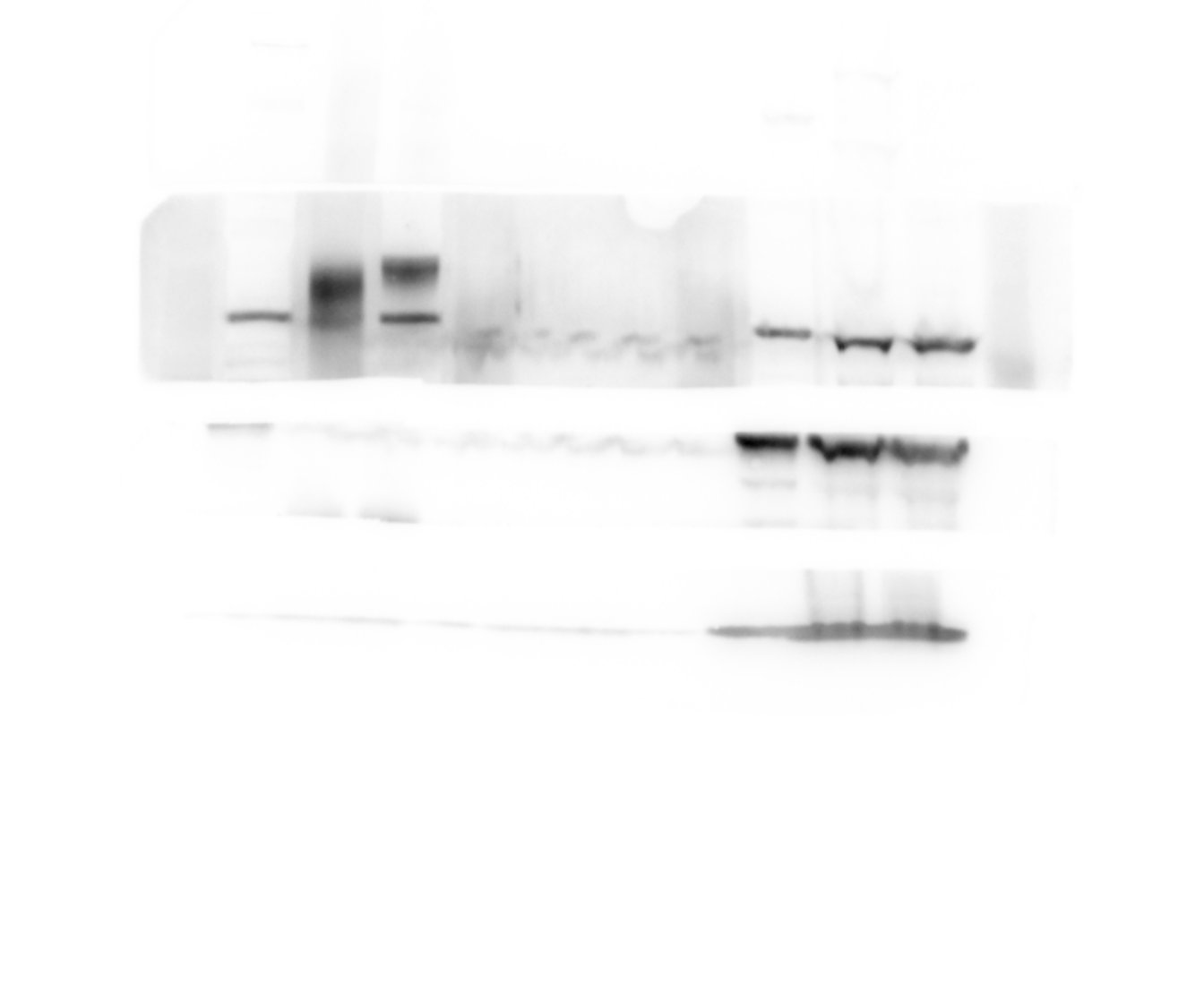


**35kDa**

**Ctrl DMSO IAA**

**Histone H3**

**GAPDH**

**Ctrl DMSO IAA**

**15kDa**


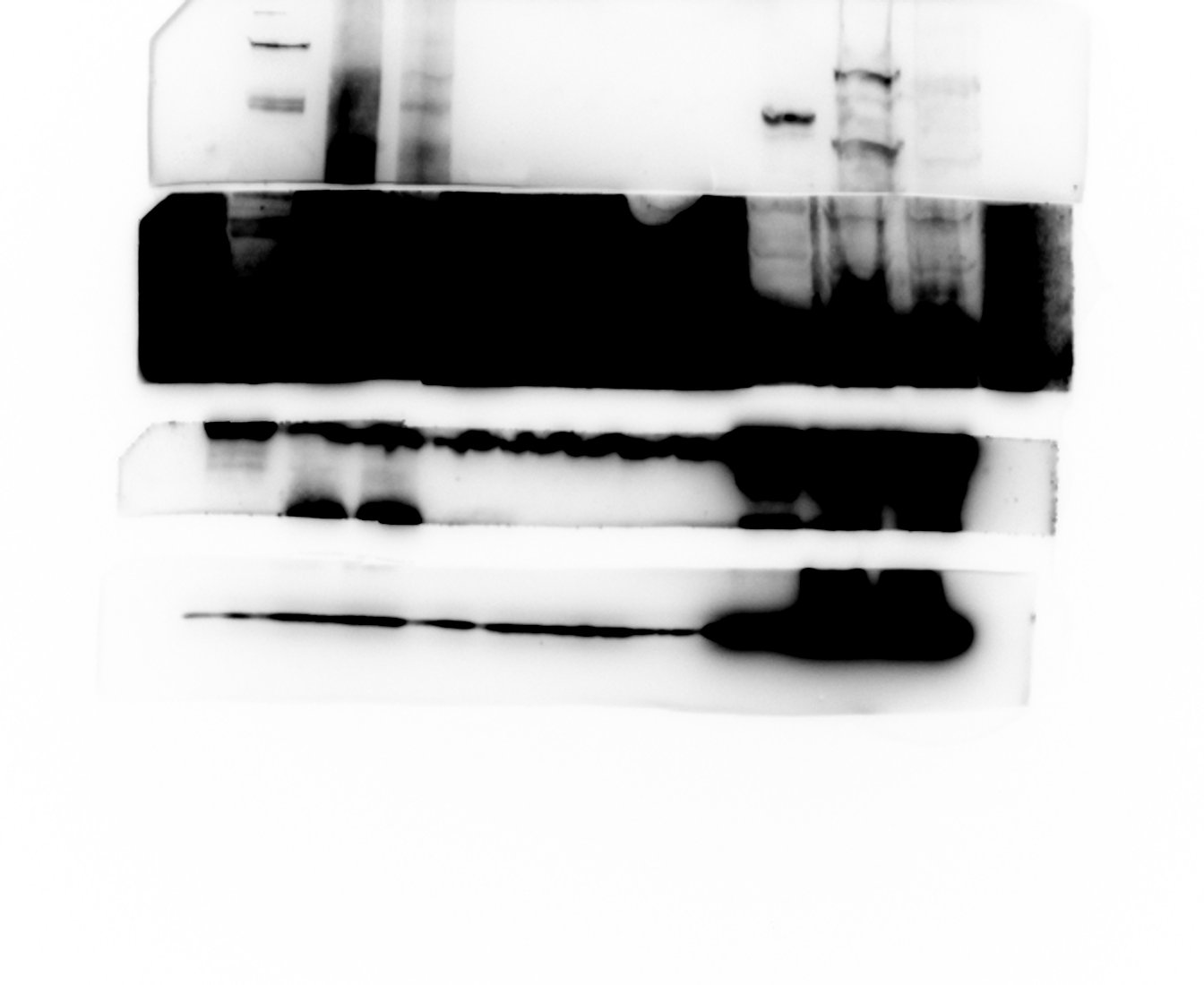
Figure3-supplement-1E-Clone2-YTHDC1

**YTHDC1**

**Ctrl DMSO IAA**

**100kDa**
