# Supplementary material for: Nuclear m6A reader YTHDC1 promotes muscle stem cell activation/proliferation by regulating mRNA splicing and nuclear export
Source: eLife. 2023 Mar 9;12:e82703. doi: 10.7554/eLife.82703 (PMC10089659; doi:10.7554/eLife.82703)
Supplement: Figure 5—source data 1. [file elife-82703-fig5-data1.zip › Figure 5 source data1/Figure 5C-with all relevant bands labelled.docx]

Figure 5C- lrp8

**Lrp8**

**ctrl2**



**iko2**

**ctrl1**

**iko1**





Figure 5C- palb2





**ctrl2**



**iko2**

**ctrl1**

**iko1**

**Palb2 1688bp**

**Palb2 293bp**

Figure 5C-GAPDH for lrp8 and palb2





**ctrl2**



**iko2**

**ctrl1**

**iko1**

**GAPDH**

Figure 5C-Scn5a


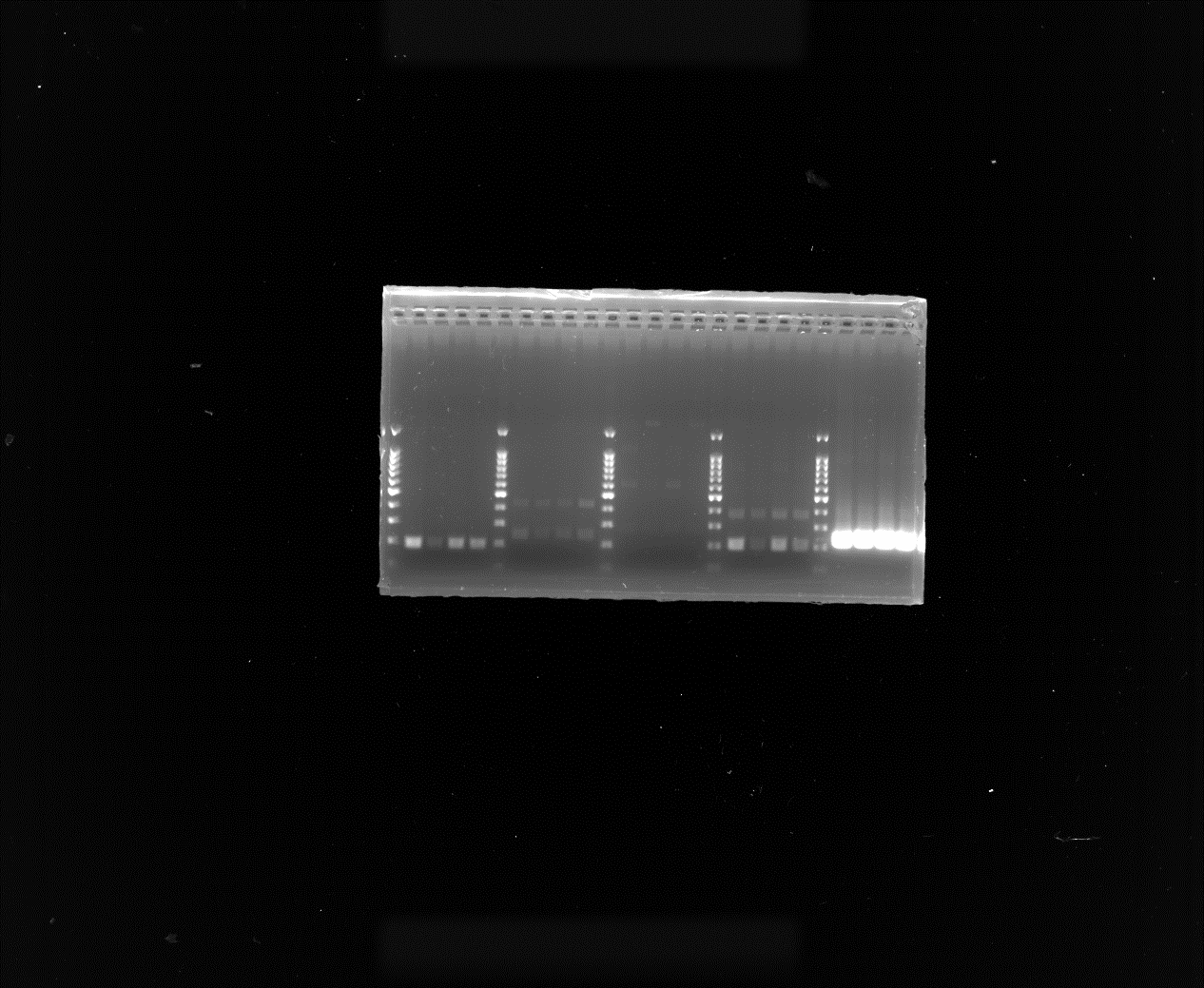


**ctrl2**



**iko2**

**ctrl1**

**iko1**

**Scn5a**

Figure 5C-gapdh for Scn5a





**GAPDH**

**ctrl2**



**iko2**

**ctrl1**

**iko1**

Figure 5C lrp8 -3rd pair for quantification, not shown in figure





**iKO3**

**Ctrl3**

**Lrp8**

Figure 5C palb2 -3rd pair for quantification, not shown in figure

**iKO3**

**Ctrl3**


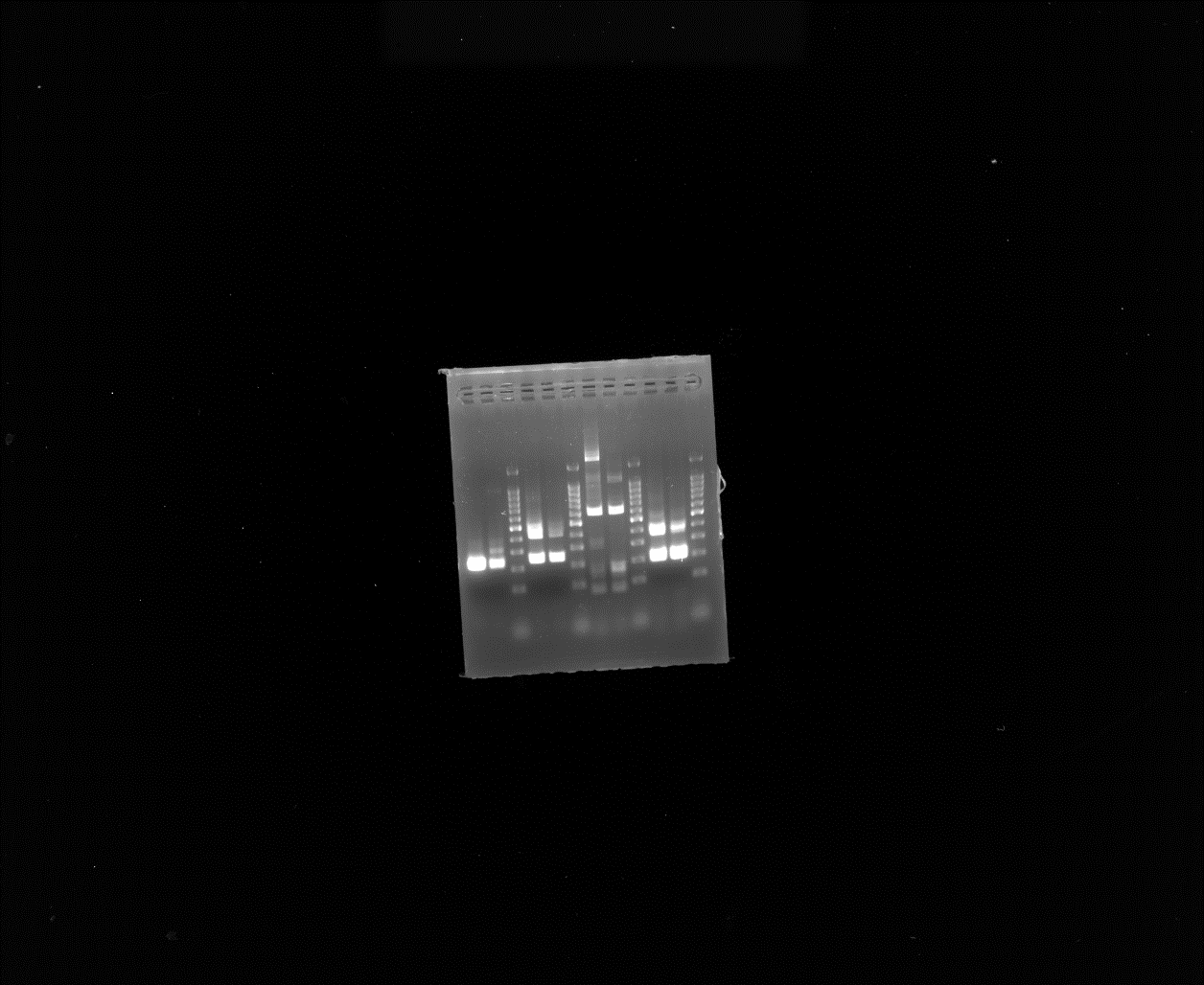


**Palb2 1688bp**

**Palb2 293bp**

**iKO3**

**Ctrl3**

Figure 5C scn5a -3rd pair for quantification, not shown in figure





**iKO3**

**Ctrl3**

**Scn5a**
