# Supplementary material for: Nuclear m6A reader YTHDC1 promotes muscle stem cell activation/proliferation by regulating mRNA splicing and nuclear export
Source: eLife. 2023 Mar 9;12:e82703. doi: 10.7554/eLife.82703 (PMC10089659; doi:10.7554/eLife.82703)
Supplement: Figure 5—source data 2. [file elife-82703-fig5-data2.zip › Figure 5 source data2/Figure 5E-with all relevant bands labelled.docx]

Figure 5E- Itgb3bp

**iko1**

**iko2**

**ctrl2**



**ctrl1**

**Itgb3bp**

Figure 5E- Nek1-exon19





**ctrl2**



**iko2**

**ctrl1**

**iko1**

**Nek1-exn19**

Figure 5E-GAPDH for itgb3bp and Nek1-exon19





**ctrl2**



**iko2**

**ctrl1**

**iko1**

**GAPDH**



Figure 5E-Itgb3bp 3rd pair for quantification, not shown in figure

**iKO3**

**Ctrl3**

**Itgb3bp**

Figure 5E-Itgb3bp 3rd pair for quantification, not shown in figure





**iKO3**

**Ctrl3**

**Nek1-exon19**
