# Supplementary material for: Nuclear m6A reader YTHDC1 promotes muscle stem cell activation/proliferation by regulating mRNA splicing and nuclear export
Source: eLife. 2023 Mar 9;12:e82703. doi: 10.7554/eLife.82703 (PMC10089659; doi:10.7554/eLife.82703)
Supplement: Figure 7—source data 1. [file elife-82703-fig7-data1.zip › Figure 7 source data1/Figure 7B-with all relevant bands labelled.docx]

Figure 7B-Anti-Flag





**pRK5-Flag-YTHDC1**

**pRK5**

**Anti-Flag**

Merged with marker


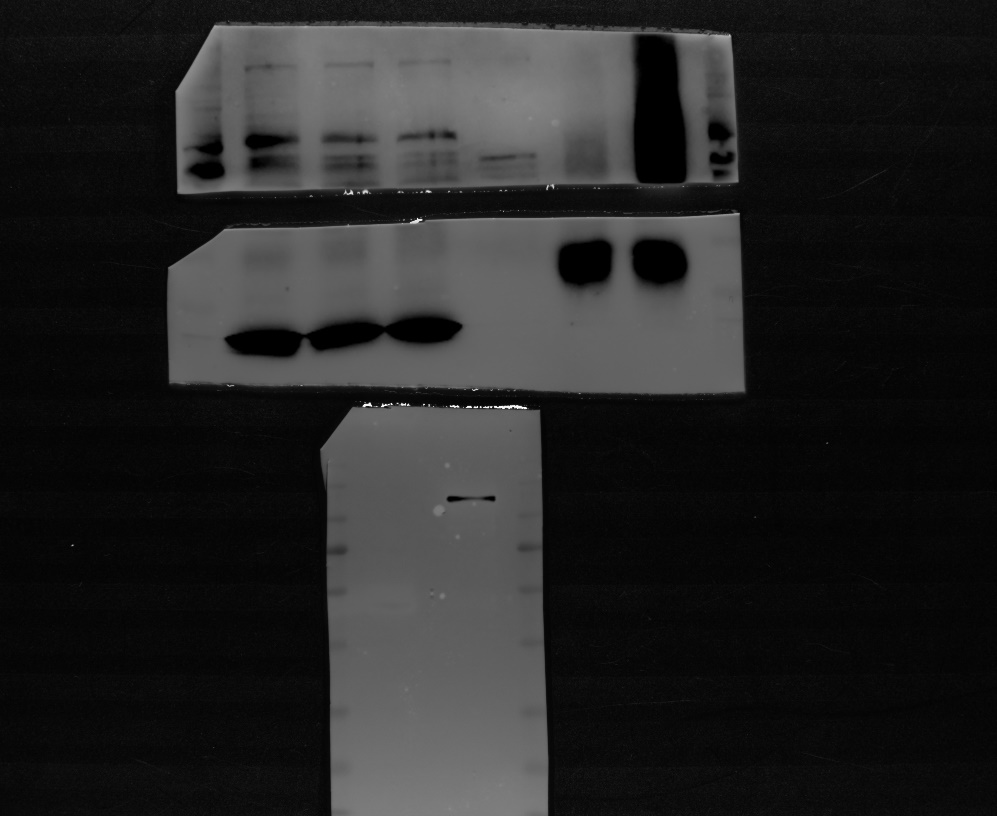


**pRK5-Flag-YTHDC1**

**100kDa**

**Anti-Flag**

**pRK5**
