# Supplementary material for: Nuclear m6A reader YTHDC1 promotes muscle stem cell activation/proliferation by regulating mRNA splicing and nuclear export
Source: eLife. 2023 Mar 9;12:e82703. doi: 10.7554/eLife.82703 (PMC10089659; doi:10.7554/eLife.82703)
Supplement: Figure 7—source data 1. [file elife-82703-fig7-data1.zip › Figure 7 source data1/Figure 7H-with all relevant bands labelled.docx]

Figure 7H-IP with flag-beads

**IP:pRK5-flagYTHDC1**

**Input: pRK5-flagYTHDC1**

**Input:pRK5-vector**

**Anti-YTHDC1**

**Anti-hnRNPG**

**IP:pRK5-vector**





**IP:pRK5-flagYTHDC1**

**Input: pRK5-flagYTHDC1**

**IP:pRK5-vector**

**Input:pRK5-vector**

**Anti-YTHDC1**

**Anti-hnRNPG**

Merged with marker


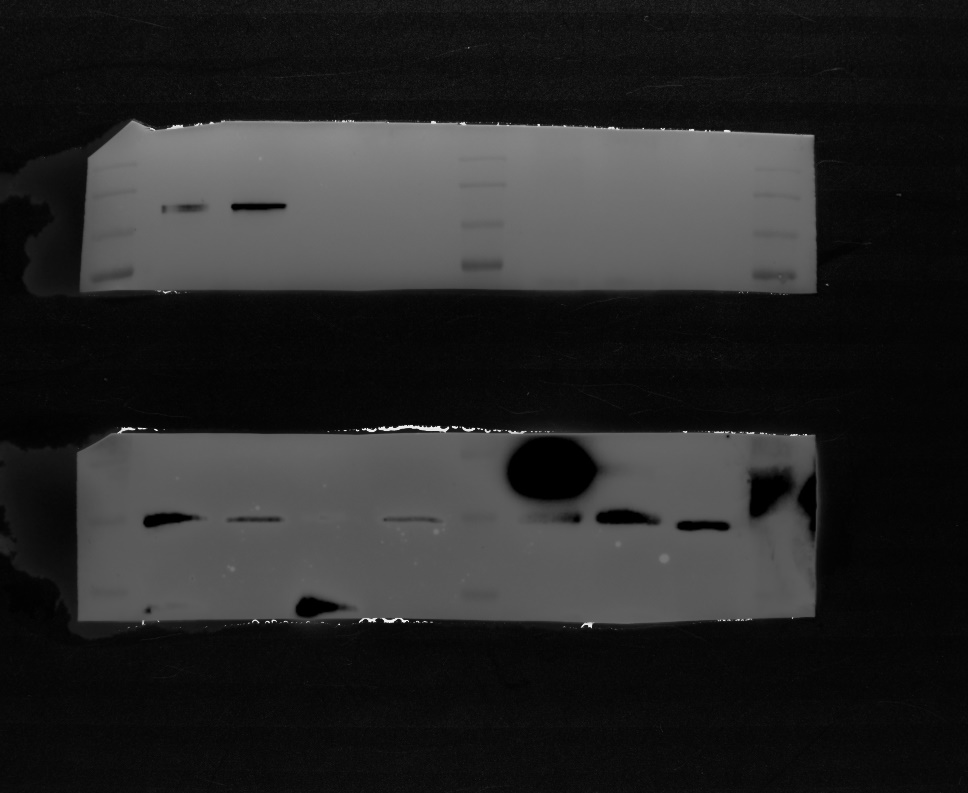


**45kDa**

**100kDa**
