# Supplementary material for: Nuclear m6A reader YTHDC1 promotes muscle stem cell activation/proliferation by regulating mRNA splicing and nuclear export
Source: eLife. 2023 Mar 9;12:e82703. doi: 10.7554/eLife.82703 (PMC10089659; doi:10.7554/eLife.82703)
Supplement: Figure 7—source data 2. [file elife-82703-fig7-data2.zip › Figure 7 source data2/Figure 7I-with all relevant bands labelled.docx]

**Figure 7I-C2C12 endogenous IP-anti YTHDC1**

**Input**

**IP:YTHDC1**

**IP:IgG**

**100kDa**


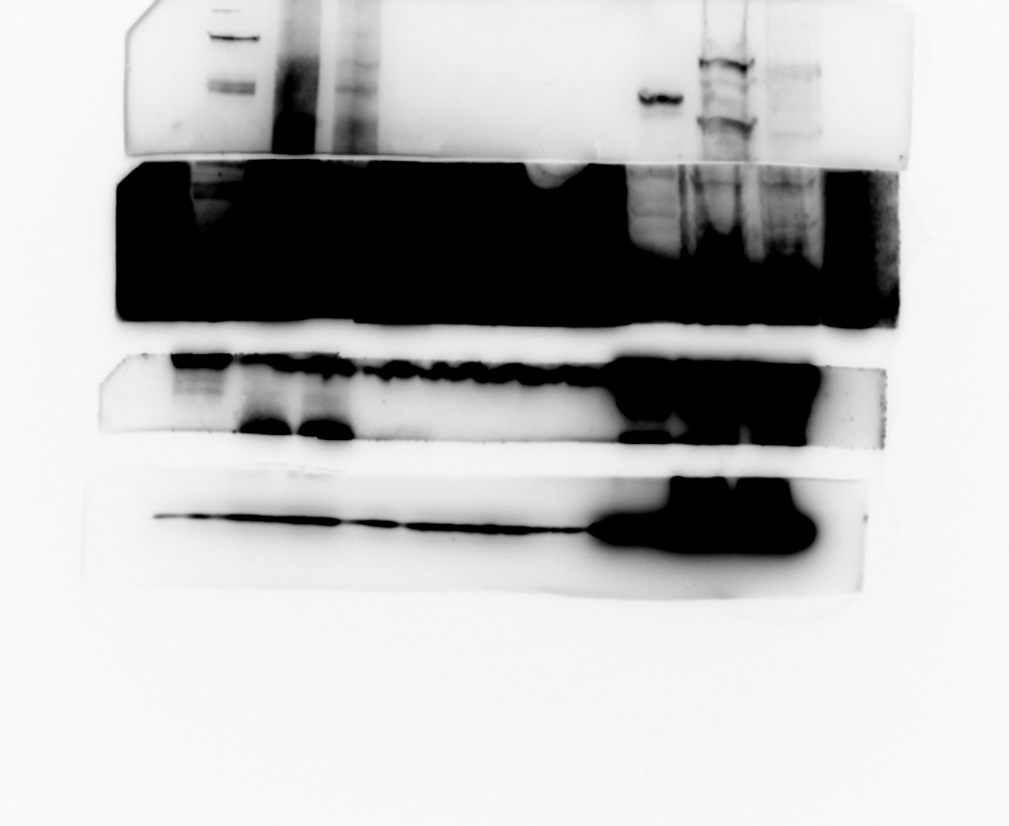


**Anti-YTHDC1**

Figure 7I-C2C12 endogenous IP-anti YTHDC1





**Arrow indicates Rabbit IgG**

**45kDa**

**Anti-hnRNPG**

**IP:IgG**

**IP:YTHDC1**

**Input**
